# Supplementary material for: Lineage commitment of dermal fibroblast progenitors is controlled by Kdm6b‐mediated chromatin demethylation
Source: EMBO J. 2023 Aug 21;42(19):e113880. doi: 10.15252/embj.2023113880 (PMC10548174; doi:10.15252/embj.2023113880)
Supplement: Supplementary file 1 — Expanded View Figures PDF [file EMBJ-42-e113880-s002.pdf]

Expanded View Figures

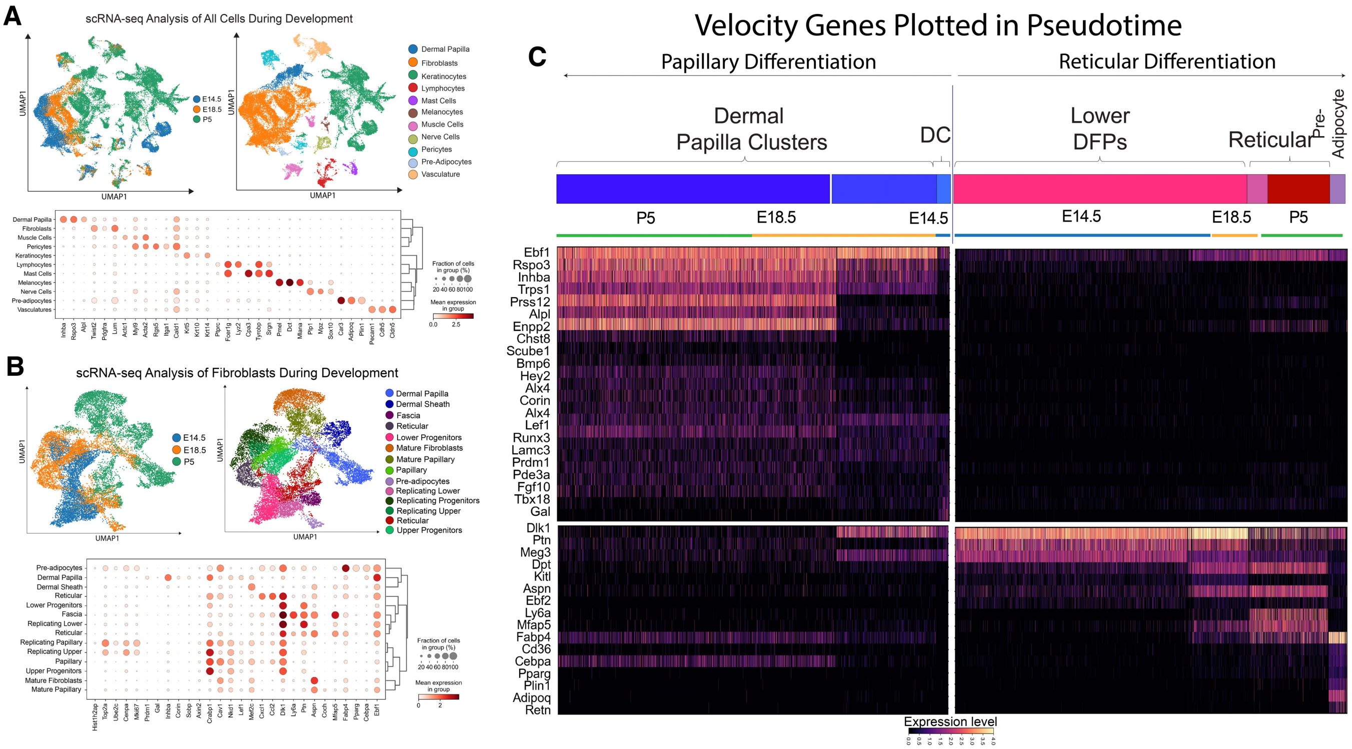

Figure EV1. scRNA-seq analysis.

A scRNA-seq analysis of all cells from E14.5, E18.5, and P5 skin.

B scRNA-seq analysis of fibroblast subset re-clustered from E14.5, E18.5, and P5 skin.

C Pseudotime-heatmap depicting the differentiation projections of Upper Progenitors to Dermal Papilla and Lower Progenitors to Preadipocyte.

© 2023 The Authors

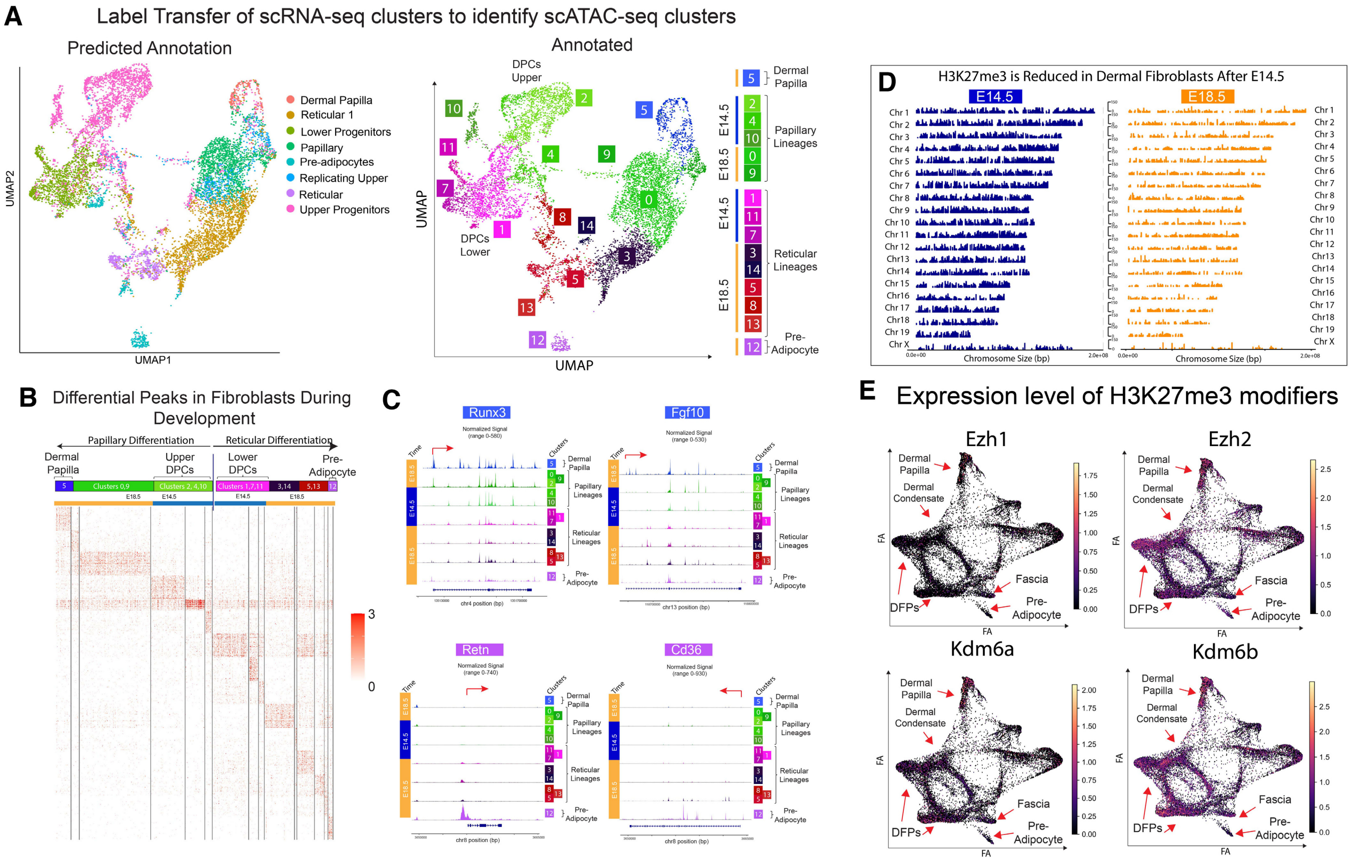

Figure EV2. scATAC-seq analysis and expression of epigenetic H3K27-me3 modifiers in E14.5, E18.5, and P5 fibroblast populations.

A Label transfer experiment of scRNA-seq cluster to identify scATAC-seq cluster in E14.5 and E18.5 skin.

B Differential peak analysis of scATAC-seq clusters arranged in pseudotime.

C Trackplot of fibroblast clusters arranged in pseudotime for Runx3, Fgf10, Retn, and CD36.

D Coverage Plots highlighting the reduction of H3K27me3 from E14.5 to E18.5 dermal fibroblasts.

E Expression of epigenetic modifiers overlayed in scRNA-seq UMAPs of fibroblasts during murine skin development.

 © 2023 The Authors

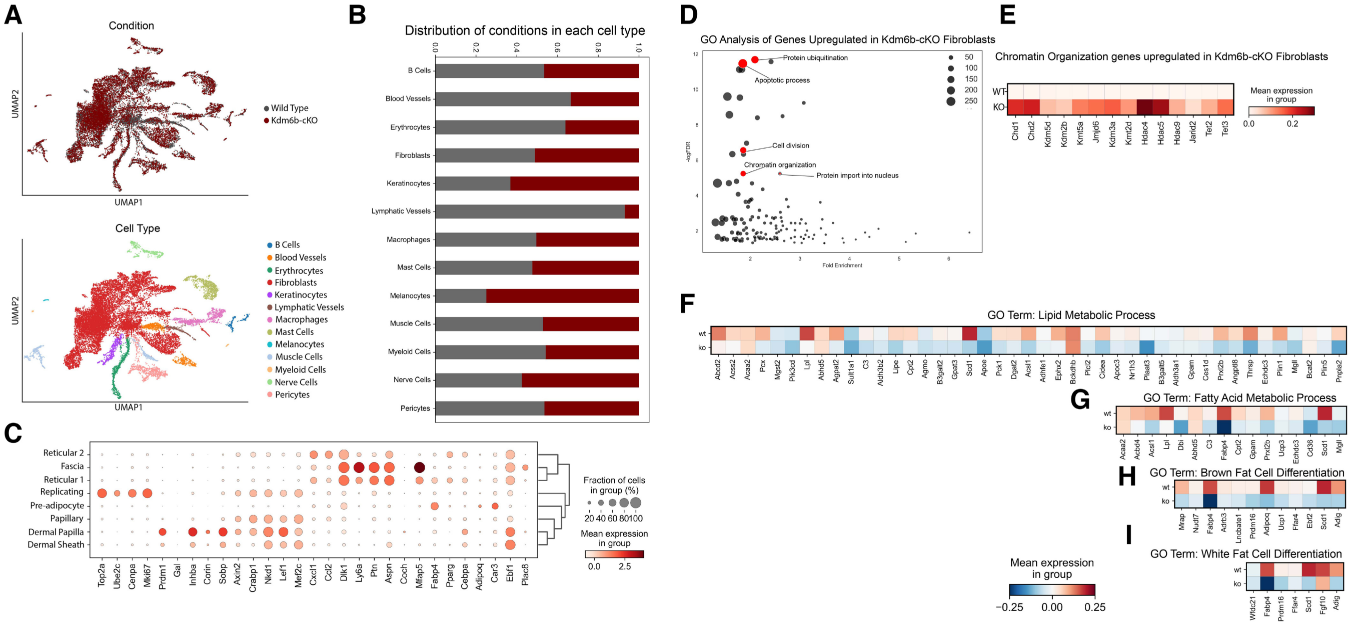

Figure EV3. scRNA-seq analysis of all cells in WT and Dermo1Cre-Kdm6b$^{fl/fl}$ skin and differential expression analysis of genes from GO Analysis of WT and Kdm6b-cKO.

A UMAP projections of WT and Dermo1Cre-Kdm6b$^{fl/fl}$ skin.

B Quantification of the number of cells within each cluster type based on genotype represented as a percentage in the cluster.

C Dotplot of gene utilized to identify cell clusters.

D GO analysis of differentially regulated genes comparing WT and Dermo1Cre-Kdm6b$^{fl/fl}$ skin.

E Gene expression of Chromatin Organization genes from GO Analysis.

F–I Differential expression of GO-Terms genes from (F) Lipid Metabolic Process (G) Fatty Acid Metabolic Process (H) Brown Fat Cell Differentiation and (I) White Fat Cell Differentiation between WT and Kdm6b-cKO fibroblasts.

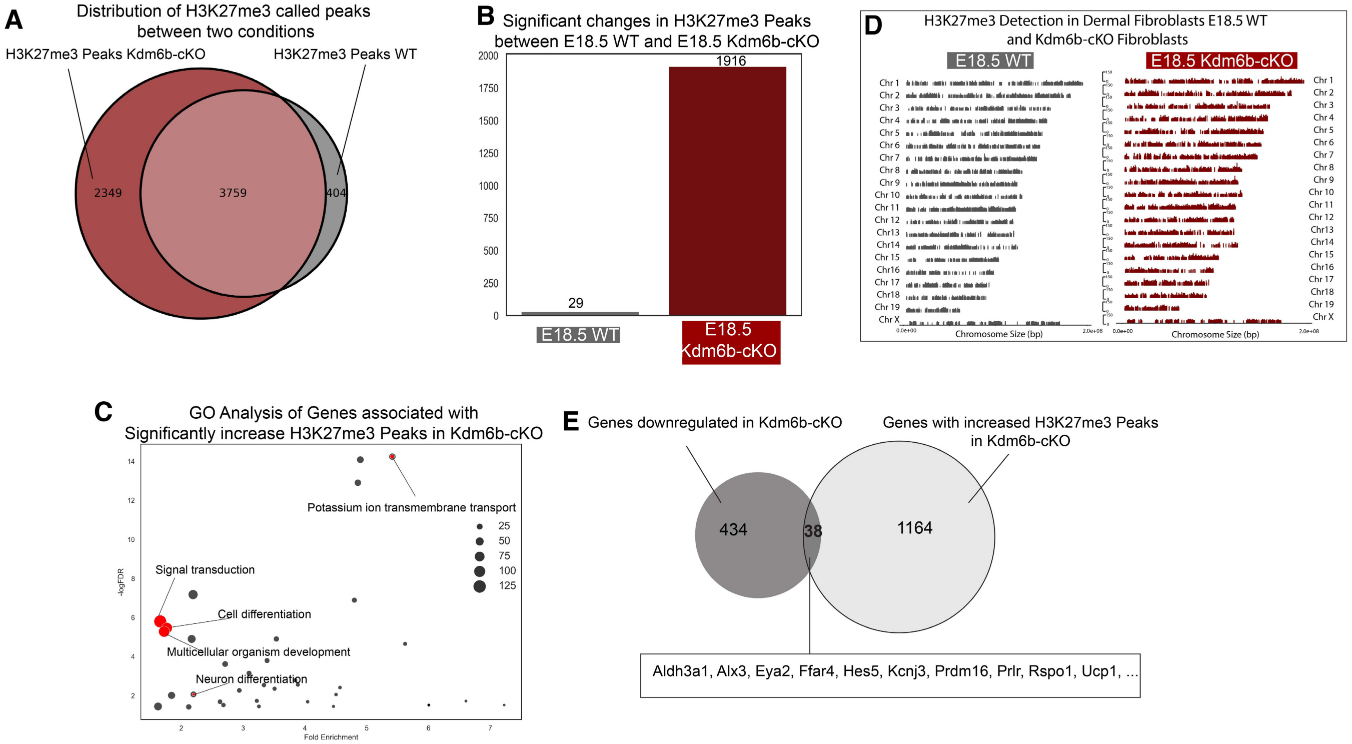

Figure EV4. Integrative analysis of H3K27-me3 peaks between WT and Dermo1Cre-Kdm6b$^{fl/fl}$ fibroblasts with integrated motif analysis in scATAC data and scRNA-seq data.

A Venn diagram comparing H3K27me3 peaks between WT and Dermo1Cre-Kdm6b$^{fl/fl}$ fibroblasts.
B Quantitation of significantly called peaks from WT and Dermo1Cre-Kdm6b$^{fl/fl}$ fibroblasts.
C GO Analysis of peaks significantly increased in Dermo1Cre-Kdm6b$^{fl/fl}$ fibroblasts
D Coverage plots highlighting the increase in H3K27me3 peaks in Kdm6b-cKO.
E Venn diagram comparing downregulated genes in scRNA-seq analysis and peaks in ChIPseq analysis.

 © 2023 The Authors

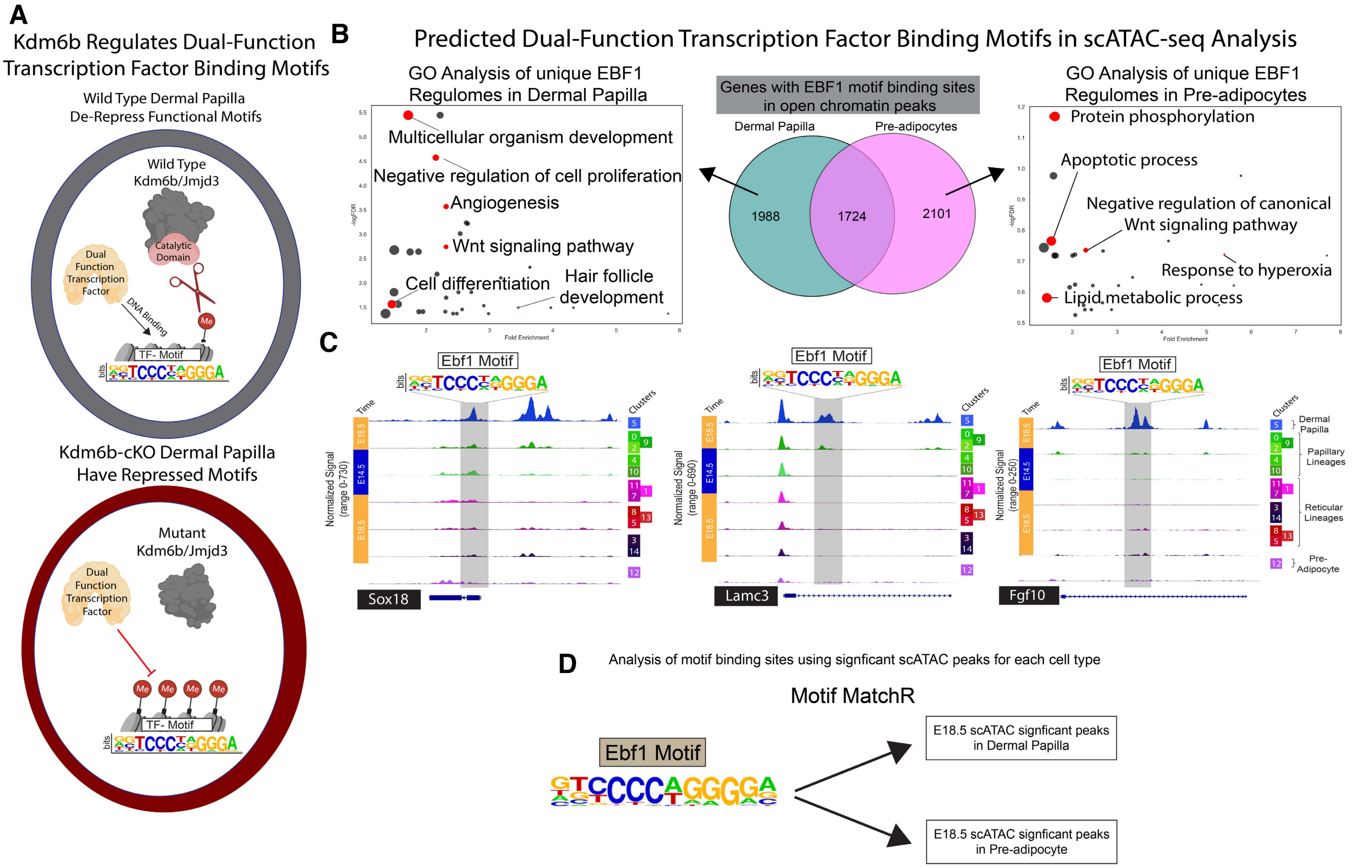

Figure EV5. Kdm6b regulates dual function transcription factor bindings sites.

A Model for Dual-Function Transcription Factor regulation of Dermal Papilla activity regulated by Kdm6b/Jmjd3 de-repression.

B Dual-Function Transcription Factor, Ebf1, binding activities represented by a GO-analysis of Ebf1 motifs in scATAC-seq peaks from Dermal Papilla and Preadipocytes fibroblast populations.

C scATAC-seq track-plot of Dermal Papilla genes, Sox18, Lamc3, and Fgf10. Predicted Ebf1 motifs are highlighted in a gray box on top of the peak that is predicted to have the motif.

D Motif analysis of scATAC peaks for GO analysis.
