# Supplementary material for: Lineage commitment of dermal fibroblast progenitors is controlled by Kdm6b‐mediated chromatin demethylation
Source: EMBO J. 2023 Aug 21;42(19):e113880. doi: 10.15252/embj.2023113880 (PMC10548174; doi:10.15252/embj.2023113880)
Supplement: Supplementary file 2 — PDF+ [file EMBJ-42-e113880-s001.pdf]

Article

SOURCE DATA | TRANSPARENT PROCESS | OPEN ACCESS

THE EMBO JOURNAL

Lineage commitment of dermal fibroblast progenitors is controlled by Kdm6b-mediated chromatin demethylation

Quan M Phan[1], Lucia Salz[2], Sam S Kindl[1], Jayden S Lopez[1], Sean M Thompson[1], Jasson Makkar[1], Iwona M Driskell[1] & Ryan R Driskell[1,3,*]

Abstract

Dermal Fibroblast Progenitors (DFPs) differentiate into distinct fibroblast lineages during skin development. However, the epigenetic mechanisms that regulate DFP differentiation are not known. Our objective was to use multimodal single-cell approaches, epigenetic assays, and allografting techniques to define a DFP state and the mechanism that governs its differentiation potential. Our initial results indicated that the overall transcription profile of DFPs is repressed by H3K27me3 and has inaccessible chromatin at lineage-specific genes. Surprisingly, the repressive chromatin profile of DFPs renders them unable to reform the skin in allograft assays despite their multipotent potential. We hypothesized that chromatin derepression was modulated by the H3K27me3 demethylase, Kdm6b/Jmjd3. Dermal fibroblast–specific deletion of Kdm6b/Jmjd3 in mice resulted in adipocyte compartment ablation and inhibition of mature dermal papilla functions, confirmed by additional single-cell RNA-seq, ChIP-seq, and allografting assays. We conclude that DFPs are functionally derepressed during murine skin development by Kdm6b/Jmjd3. Our studies therefore reveal a multimodal understanding of how DFPs differentiate into distinct fibroblast lineages and provide a novel publicly available multiomics search tool.

Keywords dermal papilla; epigenetics; fibroblast progenitor; Kdm6b; multiomics
Subject Categories Chromatin, Transcription & Genomics; Development; Skin
DOI 10.15252/embj.2023113880 | Received 2 March 2023 | Revised 26 July 2023 | Accepted 1 August 2023 | Published online 21 August 2023
The EMBO Journal (2023) 42: e113880

Introduction

Fibroblasts are mesenchymal cells that create and maintain the extracellular matrix of the connective tissues supporting the functions of various organs (Watt & Fujiwara, 2011; Driskell & Watt, 2015; Plikus *et al*, 2021). They are distinct across different organs but are also diverse within the same tissue (Dhouailly & Sengel, 1975; Fernandes *et al*, 2004; Muhl *et al*, 2020; Buechler *et al*, 2021). Mammalian skin serves as a critical model to study development, homeostasis, and the role of fibroblast heterogeneity in tissue-resident stem cell niche, tissue regeneration, fibrosis-associated diseases, and the effects of chronic aging (Singer & Clark, 1999; Gurtner *et al*, 2008; Chen *et al*, 2013; Cole *et al*, 2018; Griffin *et al*, 2020; Gruber *et al*, 2020). Recent studies have demonstrated the distinct roles of dermal fibroblast subpopulations during embryonic development and skin wound healing. These studies have revealed that manipulations of specific fibroblast subpopulations lead to hair follicles and adipocytes regeneration in different wounding scenarios (Rinkevich *et al*, 2015; Plikus *et al*, 2017; Jiang *et al*, 2018; Correa-Gallegos *et al*, 2019; Guerrero-Juarez *et al*, 2019; Abbasi *et al*, 2020; Phan *et al*, 2020; Shook *et al*, 2020; Mascharak *et al*, 2021; Sinha *et al*, 2022). However, the establishment of fibroblast heterogeneity from a common progenitor during embryonic development remains poorly characterized.

During early embryonic development, mesenchymal stem cells migrate from different embryonic origins to subepidermal regions of the skin, where the dorsal dermis arises from the somite, and the facial dermal fibroblasts derive from the neural crest (Dhouailly, 1973; Fernandes *et al*, 2004; Atit *et al*, 2006; Wong *et al*, 2006; Tran *et al*, 2010; Thulabandu *et al*, 2018). Approximately E13.5–14.5 in murine skin, reciprocal signaling between the overlying epidermis and the Dermal Fibroblast Progenitors (DFPs) leads to condensation of upper fibroblasts to form the placodes (Kishimoto *et al*, 2000; Millar, 2002; Chen *et al*, 2012; Fu & Hsu, 2013). Between E14.5 and E16.5, the DFPs then differentiate and commit to distinct lineages of upper papillary and lower reticular fibroblasts (Driskell *et al*, 2013). Upper papillary fibroblasts differentiate into the dermal papilla, neonatal papillary fibroblast, and the arrector pili muscles postnatal. Lower reticular fibroblasts differentiate into collagen-secreting reticular and the dermal white adipocyte (DWAT; Festa *et al*, 2011; Wojciechowicz *et al*, 2013). Moreover, classic chimeric skin grafting

1 School of Molecular Biosciences, Washington State University, Pullman, WA, USA
2 North Rhine-Westphalia Technical University of Aachen, Aachen, Germany
3 Center for Reproductive Biology, Washington State University, Pullman, WA, USA
*Corresponding author. Tel: +1 509 335 3807; E-mail: ryan.driskell@wsu.edu

© 2023 The Authors. Published under the terms of the CC BY 4.0 license

studies between avian and murine skin have shown that DFPs need to differentiate before responding to epidermal signals, and that fibroblast identity is also region-specific (Dhouailly, 1973). Altogether, during embryonic development, we propose that DFPs must undergo an intrinsic reprogramming process to commit to fibroblast lineages and differentiate toward the terminal fates.

Epigenetic regulators have been extensively studied in the context of cellular differentiation (Boyer *et al*, 2006; Lee *et al*, 2006; Kang *et al*, 2019; Flora & Ezhkova, 2020; Plikus *et al*, 2021). A proposed mechanism of chromatin regulation is through the addition of methyl groups to Histone 3 Lysine 27 (H3K27), which is associated with condensed and silenced chromatin while the removal of methyl group is associated with accessible and active chromatin (Cao *et al*, 2002; Agger *et al*, 2007; Xiang *et al*, 2007; Kang *et al*, 2019). The most widely known regulators of methylation on H3K27 are part of the Polycomb repressive complex 2 (PRC2), EZH1 and EZH2. In the epidermis, the addition and removal of the trimethyl group (me3) on H3K27me3 were shown to be critical in controlling the differentiation process of epidermal stem cells during development (Sen *et al*, 2008; Ezhkova *et al*, 2009). Demethylases, such as KDM6A/UTX and KDM6B/JMJD3, specifically remove methyl group and derepress chromatin (Agger *et al*, 2007; Xiang *et al*, 2007). In dermal fibroblasts, EZH2 has been shown to act as an epigenetic rheostat to control the Wnt and Retinoic Acid signaling in DFPs (Thulabandu *et al*, 2021). However, a full understanding of how the methylation status of H3K27 regulates fibroblast differentiation has not been shown. In addition, other studies have also investigated the roles of different epigenetic marks in regulating and maintaining fibroblast lineages in developed the skin. Acetylation of H3K27 and HDAC2 activities are required for the maintenance of Papillary Fibroblast lineages postnatally (Kim *et al*, 2022). H3K4me3, H3K9me3, and H3K27me3 all display reduced methylation levels in hair follicle stem cells prior to hair growth, and inhibition of hypomethylation delays skin wound healing (Lee *et al*, 2016; Kang *et al*, 2020). Nonetheless, an epigenetic regulation that programs dermal fibroblast lineage commitment and differentiation during embryonic development remains unknown.

The recent emergence of single-cell technology has advanced the study of fibroblast heterogeneity drastically (Plikus *et al*, 2017). The transcriptomic changes in restricted fibroblast lineages, specifically in the hair follicle–associated fibroblasts, have been characterized at different timepoints that regulate Dermal Papilla establishment (Biggs *et al*, 2018; Gupta *et al*, 2019; Mok *et al*, 2019). In our recent study of transcriptomic and chromatin landscapes at the single-cell level, we showed that the transcriptomic profiles of fibroblast lineages could only reflect the cellular states, while the accessible chromatin profiles can predict the lineage restriction and terminal fate commitment (Thompson *et al*, 2022). Overall, we hypothesize that the differentiation of dermal fibroblast progenitors could be defined by incorporating chromatin accessibility profiles and epigenetic regulation.

To establish the multimodal reference point for dermal fibroblast progenitors, we compared the molecular states of dermal fibroblast progenitors (DFP) at E14.5 to late embryonic fibroblasts at E18.5 using single-cell transcriptomics, single-cell ATAC, ChIP-seq, genetic ablation of epigenetic regulator Kdm6b/Jmjd3, and functional chamber allografting assays. We found that DFPs at E14.5 are predicted to differentiate into hair follicle–associated fibroblast, but they are not intrinsically programmed as they failed to support hair follicle formation *ex vivo*. E14.5 DFPs possess a closed-off chromatin profile, marked by a high level of H3K27me3, and they require epigenetic programming mediated by Kdm6b/Jmjd3 to commit to specific fibroblast lineages. Finally, we share our processed data for all our experiments on our website skinregeneration.org.

Results

DFPs differentiate into distinct fibroblast lineages *in vivo* but fail to regenerate functional skin in grafting assays

Using lineage tracing assays, we have previously shown that DFPs are present throughout the dermis at E14.5 and that a DFP has the potential to contribute to the formation of all layers and fibroblast subtypes (Driskell *et al*, 2013; Rognoni *et al*, 2016). We hypothesized that E14.5 fibroblasts are multipotent DFP progenitors in developing murine skin. We performed scRNA-seq from whole skin samples of E14.5, E18.5, and P5 mice (Fig 1A–E). Altogether, we captured and sequenced a total of 43,740 cells, of which 17,356 cells were classified as dermal fibroblasts (Fig EV1A and B). To evaluate DFP differentiation and developmental potential, we integrated all three time points together and embedded the single-cell clusters as a PAGA map to improve interpretation but also to preserve the global topology of cells transition (Fig 1D–E; Materials and Methods) (Hie *et al*, 2019; Wolf *et al*, 2019). DFPs at E14.5 were classified into two distinct subsets based on their horizontal anatomical positions as previously published (Gupta *et al*, 2019; preprint: Jacob *et al*, 2022). Upper DFPs (clusters 0, 2, 11) expressing Crabp1, Cav1, Nkd1, and Lef1 resided closer to the epidermis, while lower DFPs expressed Thbs1, Ptn, and Mfap5 (clusters 1, 5, 6) (Figs 1A and D–F, and EV1A and B). Furthermore, fibroblast subsets in E18.5 and P5 populations were identified based on gene expression profiles that have been previously defined (Fig EV1A and B) (Guerrero-Juarez *et al*, 2019; Phan *et al*, 2020; Thompson *et al*, 2022). We identified clusters of Dermal Papilla, Papillary Fibroblasts, Reticular Fibroblasts, Fascia, and Preadipocytes in both E18.5 and P5 skin. In addition, clusters of mature papillary and ECM-producing fibroblasts were detected only in P5 skin, reflecting the maturation of postnatal dermal fibroblasts. Our PAGA integrated analysis revealed that most E18.5 fibroblasts were closely associated with E14.5 DFPs while P5 fibroblasts clustered distinctly based on their lineage functions, except in the lineage-committed specialized fibroblasts such as Dermal Papilla and Preadipocytes, where E18.5 and P5 clustered together (Fig 1D–E).

To further establish the continuous transition between DFPs and fibroblast lineages, we performed RNA velocity analysis on the PAGA-embedded fibroblast subsets, which accounted for the transcriptional dynamics of splicing kinetics within each cell (Bergen *et al*, 2020). Upper DFPs are shown to differentiate into papillary fibroblasts (clusters 7, 8, 16, 21), dermal papillae (clusters 4, 12, 20), dermal sheath (clusters 10), and mature fibroblasts (clusters 3, 17), while lower DFPs are projected to become upper DFPs and reticular (clusters 9, 13, 15, 18; Fig 1D–E) (Driskell *et al*, 2014; Correa-Gallegos *et al*, 2019; Guerrero-Juarez *et al*, 2019; Joost *et al*, 2020; Shook *et al*, 2020). Unexpectedly, fascia and preadipocytes showed inverted RNA velocity trajectories toward replicating

 © 2023 The Authors

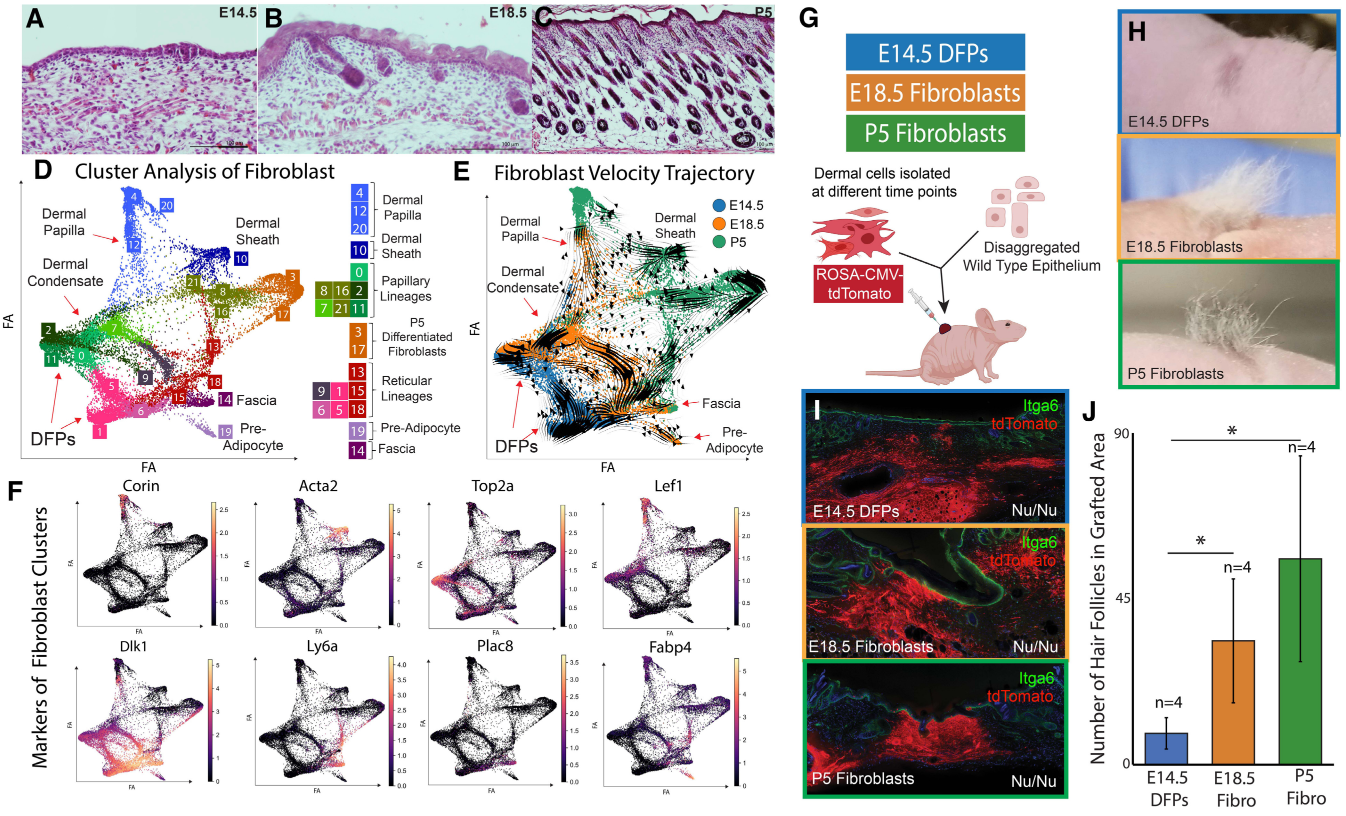

Figure 1. DFPs are not intrinsically programmed to support skin reformation.

A–C H&E histology of murine skin sections at Embryonic Day E14.5 (A), E18.5 (B), and Post-natal day 5 (P5) (C).

D–E PAGA-initialized single-cell embedding of fibroblasts of 3 timepoints integrated. (F) Expression of Fibroblast Marker Genes.

G–J (G) *Ex vivo* chamber grafting assay testing the ability of fibroblast populations at E14.5, E18.5, and P5 to support hair follicle formation. (H) Photographs of area of reconstituted skin. (I) Histology of reconstituted skin in chamber grafting assay. (J) Quantification of the number of hair formations in grafted area.

Source data are available online for this figure.

reticular fibroblasts. This observation could be due to the multiple rate kinetics genes (MURK) (Barile *et al*, 2021), which are associated with assumptions and caveats for velocity analysis in scRNA-seq. For example, dynamical MURK genes can violate the assumptions that velocity analysis is based on (Barile *et al*, 2021). In addition, it has been proposed that current batch correction implementations may not be accurate (Bergen *et al*, 2021). Consequently, it is important to test in-silico transcriptome-based results through robust independent systems. Moreover, we have noticed that a large portion of fascia and preadipocytes is actively replicating and clustered closely with replicating lower reticular (cluster 15). The newly generated unspliced mRNA is higher in the replicating population, contributing to the inversion of RNA velocity trajectory. RNA velocity analysis also provided us with a list of putative driver genes that need to be activated for cells to differentiate toward a specific cluster. Using these genes, we generated a heatmap depicting the pseudotime transitions of upper and lower DFPs into two distinct lineages: Dermal Papilla and Preadipocytes (Fig EV1C). We found that multiple velocity driver genes of E18.5 and P5 Dermal Papilla were expressed as early as E14.5 Dermal Condensate (Fig EV1C). Overall, our results suggested that at E14.5, DPFs have the potential to differentiate into specialized cell types to support and maintain the different dermal niches in postnatal skin.

Grafting assays, in which cells are transplanted into host tissues and allowed to interact with the surrounding microenvironment, provide a powerful tool to assess the differentiation potential and lineage restrictions of stem cells *ex vivo* (Jensen *et al*, 2010; Driskell *et al*, 2013). To functionally challenge the potential of E14.5 DFPs to support the formation of fully functional skin, we performed a skin reconstitution experiment utilizing the chamber grafting technique. We collected whole, unsorted DFPs from E14.5 skin, and compared their ability to reform hair follicles and skin from fibroblasts from E18.5 and P5 ROSA-CMV-tdTomato-positive mice to trace their contribution within grafted tissue. A combination of dermal cells and epidermal cells was injected into a silicone chamber grafted on top of an immunocompromised Foxn1$^{-/-}$ mouse (Fig 1G). Skin from areas of the graft was collected at 28 days postsurgery to assess the reformation and development of the skin. The analysis and quantification of the grafting assay revealed that E18.5 and P5 differentiated fibroblast populations were able to support the reformation of fully functional hair follicles and hair fibers while E14.5 DFPs failed to regenerate and develop hair follicles despite their contribution to

© 2023 The Authors

skin reformation (Fig 1H–J). This result suggests that, despite the potential trajectory and presence of early dermal papilla cells, DFPs from E14.5 skin are not intrinsically programmed to differentiate ex vivo to support fully functional skin reformation.

DFP programming occurs by specifically increasing the accessibility of chromatin architecture in distinct lineages

The chromatin accessibility landscape regulates cellular gene expression and is highly informative of its states and fates of cells (Buenrostro *et al*, 2013, 2015; Cusanovich *et al*, 2015, 2018; Trapnell, 2015; Cao *et al*, 2017; Thompson *et al*, 2022). We hypothesized that the intrinsic differentiation program within E14.5 DFPs could be reflected in its chromatin accessibility profiles. We performed single-cell ATAC-seq assays on murine DFPs at E14.5 and differentiating fibroblasts at E18.5. After quality control, filtering, and preprocessing, 10,913 fibroblasts from both time points were used for downstream analyses including dimension reduction and unsupervised clustering (4,187 from E14.5 and 6,006 from E18.5) (Fig 2A and B). Standard quantification of the ATAC peak number per cell comparing E14.5 and E18.5 fibroblasts revealed a large increase in open chromatin regions during the differentiation process (Fig 2C). Differential peak analysis was performed using Wilcoxon testing between time identified 6,200 peaks in E14.5 and 13,566 peaks in E18.5 fibroblasts. Coverage plots depicting the significant changes in open chromatin regions between E14.5 and E18.5 highlighted the increase of accessible peaks during the differentiation process of DFPs (Fig 2D). Our results indicate that chromatin reprogramming of DFPs during differentiation is associated with a selective, derepression of the genome.

To investigate the relationship between chromatin depression and the establishment of fibroblast heterogeneity from DFPs, unsupervised clustering of scATAC-seq data was performed resulting in 15 distinct clusters from both time points (Figs 2E and EV2A). Cross-modality integration and label transfer were used to integrate scATAC-seq with scRNA-seq data for cluster identification (Materials and Methods; Fig EV2A) (Stuart *et al*, 2021). Consistent with our transcriptomic analysis, DFPs at E14 were split into Upper DFPs and Lower DFPs, while E18 fibroblasts contained Dermal Papilla, Papillary, Reticular, and Preadipocytes clusters (Fig 2E). Interestingly, even though fascia fibroblasts were detected in our scRNA analysis, we did not detect them in our scATAC-seq data (Fig 2E). Differential peak analysis revealed distinct chromatin landscapes among fibroblasts at E18.5, while the E14.5 Upper and Lower DFPs possessed less unique accessible peaks profiles, which was consistent with the opening of chromatin during DFPs differentiation processes (Fig EV2C). Similar to P0 fibroblasts (Thompson *et al*, 2022), E18.5 fibroblasts also shared a gradient of significant peaks within their respective lineages, with the differentiated Dermal Papilla and Preadipocytes fates possessing the most distinct chromatin profiles (Figs 2F and G and EV2B and C). Considering the predicted RNA velocity trajectories of DFPs to differentiate into Dermal Papilla and Preadipocyte, we hypothesized that the chromatin accessibility profiles of the equivalent cell types will also reflect a similar differentiation trajectory. We constructed a pseudotime heatmap based on our scATAC-seq data but defined by RNA velocity driver genes for each fibroblast cell type from DFPs to Papillary to Dermal Papilla and DFPs to Reticular to Preadipocyte (Materials and Methods; Fig 2F). We found that as Upper DFPs differentiated toward Papillary and Dermal Papilla, the velocity driver genes of Papillary and Dermal Papilla became more accessible (Fig 2F). Notably, the driver genes of Upper DFPs were accessible across all fibroblast subtypes. Dermal Papilla had accessible chromatin regions for Papillary driver genes, but DP-specific genes were not accessible in Papillary, indicating the differentiated fate of Dermal Papilla as previously reported (Fig 2F; REF). This finding confirmed the continuous transition of Upper DFPs to Papillary to Dermal Papilla, where Upper DFPs require making their chromatin landscape specifically accessible during their differentiation process. The closed chromatin profiles of DFPs at E14.5 without accessible regions for Dermal Papilla driver genes might help explain the inability of DFPs to reform hair in chamber grafting assays. Similarly, the Lower DFPs differentiation trajectory was also observed using Lower DFPs, Reticular, and Preadipocytes RNA velocity driver genes (Fig 2F). Overall, our analysis revealed that during differentiation, DFPs specifically make their chromatin accessible in regions associated with the specific driver genes at E18.5 in a stepwise manner (Fig 2F). We also concluded that the differentiated fibroblast fates of Dermal Papilla and Preadipocytes required unique reprogramming of dermal fibroblasts with specifically accessible regions such as Sox18, Stmn2 for Dermal Papilla and Fabp4, AdipoQ for Preadipocytes (Fig 2G).

Chromatin programming of DFPs is associated by the removal of the repressive histone modification H3K27me3

Posttranslational modifications of histones can regulate gene accessibility and expression during the differentiation process in various tissue models (Boyer *et al*, 2006; Lee *et al*, 2006; Heintzman *et al*, 2009; Karlić *et al*, 2010). Specifically, the Histone 3 Lysine 27 trimethylation mark (H3K27me3), which is associated with the repression of gene transcription, plays a key role in the differentiation process of epidermal keratinocytes (Sen *et al*, 2008; Ezhkova *et al*, 2009; Kang *et al*, 2019). Our scATAC-seq data suggested that the opening of chromatin regions at lineage-specific genes was critical for fibroblast differentiation. To study the role of H3K27me3 in regulating open chromatin regions of DFPs during embryonic differentiation, we performed H3K27me3 ChIP-seq comparing E14.5 and E18.5 Fibroblasts (Fig 3A). In conformity with the increase in open chromatin regions between E14.5 and E18.5 fibroblasts, we found a sharp decrease of 52% in the number of H3K27me3 peaks (from 19,060 peaks at E14.5 to 8,768 peaks at E18.5, $n = 2$) by visualizing the heatmaps and coverage plots of peaks called at each time point (Figs 3B–D and EV2D). In addition, we also confirmed that the majority of the genes associated with H3K27me3 peaks at E18.5 were present at E14.5 (95%), indicating that there was a significant reduction in H3K27me3-associated genes and not a dynamic change where undifferentiated genes were repressed and differentiated genes were derepressed (Fig 3D). We next utilized DiffBind to identify the significant changes between E14.5 and E18.5 H3K27me3 peaks in DFs, which resulted in 4699 significant peaks that decreased from E14.5 to E18.5 and only 29 peaks that increased (Fig 3E). To investigate their functions, Gene Ontology analysis of genes associated with the significantly derepressed peaks from E14.5 revealed the biological processes associated with skin differentiation and development such as multicellular organism development, cell differentiation, neuron differentiation, Canonical Wnt signaling pathway, hair follicle development, and glucose homeostasis (Fig 3F). Within this list, we

 © 2023 The Authors

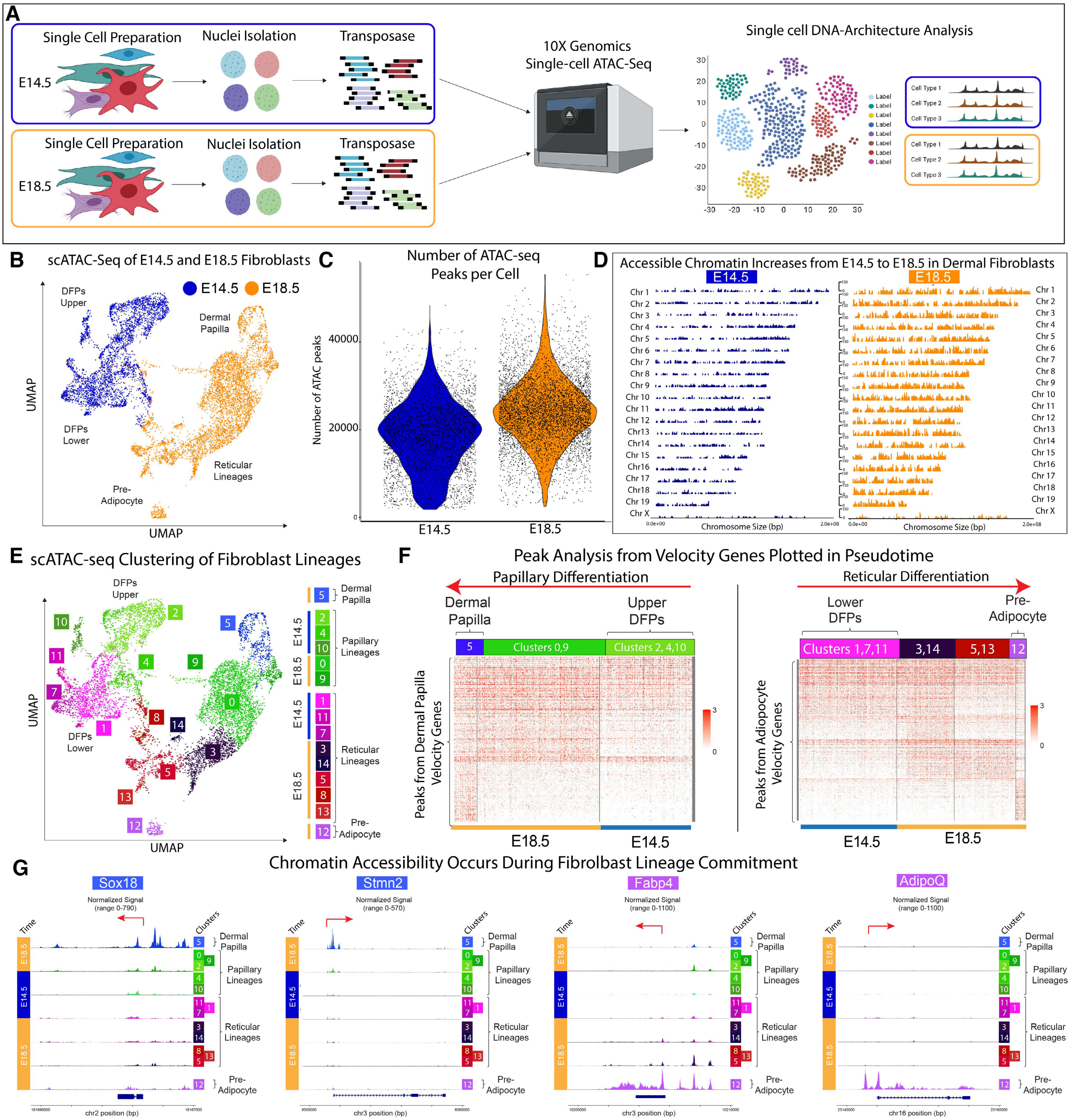

Figure 2. DFP differentiation is defined by inaccessible chromatin architecture.

A Experimental design of scATAC-seq experiments comparing E14.5 and E18.5 dermal fibroblasts.
B UMAP plot showing the distinct clustering of E14.5 and E18.5 fibroblasts.
C Coverage Plots highlighting the significant increase in open accessible chromatin regions from E14.5 to E18.5 fibroblasts.
D Quantification of Total ATAC Peaks per cell.
E UMAP plot showing the classification of fibroblast cell types in scATAC-seq data.
F Pseudotime-heatmap of scATAC-seq peaks associated with RNA velocity genes required for Dermal Papilla and Preadipocyte differentiation. Dermal Papilla and Preadipocyte populations are flanking the E14.5 DFPs which are located in the centrally.
G Tracks-plot arranged in pseudotime showing specific accessibility regions of Sox18 and Stmn2 in Dermal Papilla, Fabp4 and AdipoQ in Preadipocytes.

Source data are available online for this figure.

© 2023 The Authors

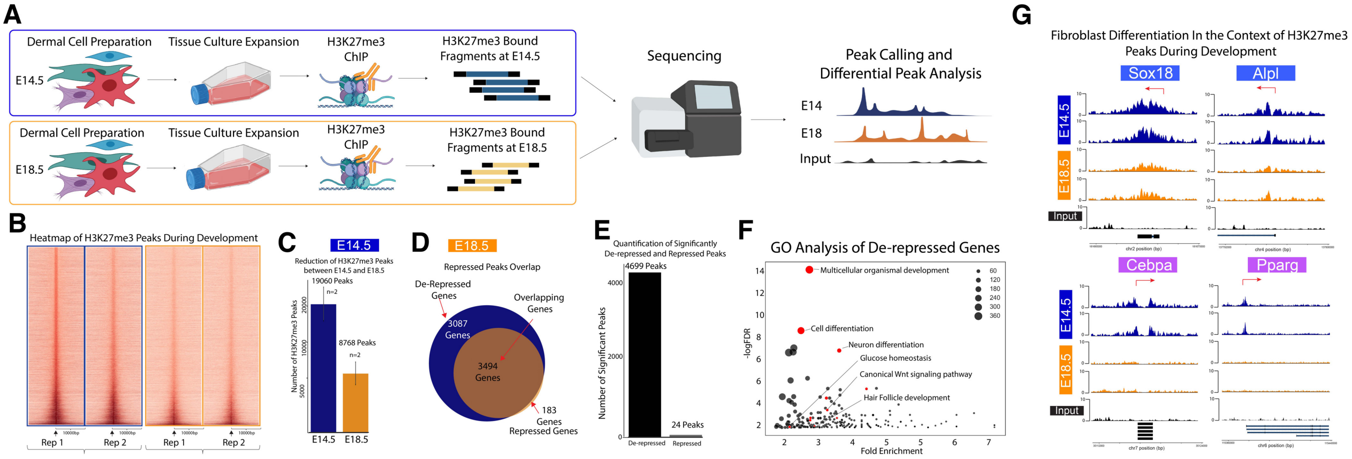

Figure 3. Differentiation of DFPs during embryonic development is coupled with demethylation of H3K27me3.

A Experimental design of H3K27me3 ChIP-seq comparing E14.5 and E18.5 Fibroblasts.
B Heatmaps of H3K27me23 Peaks comparing E14.5 and E18.5 Fibroblasts.
C Quantification showing the decrease in H3K27me3 peaks (19,060 peaks at E14.5 and 8,768 peaks at E18.5, $n = 2$).
D Venn diagram revealing the distribution of genes associated with H3K27me3 peaks changes between fibroblasts from E14.5 to E18.5.
E Quantification of significantly differentiate H3K27me3 binding between E14.5 and E18.5 fibroblasts (4,699 peaks at E14.5 and 24 peaks at E18.5).
F GO Analysis of Significantly De-repressed Genes revealed important Fibroblast differentiation processes.
G Track plot of important de-repressed genes associated with fibroblast differentiation (Sox18 and Alpl for Dermal Papilla, Cebpa and Pparg for Preadipocyte).

Source data are available online for this figure.

specifically found genes that played important roles in the establishment and differentiation of dermal niches such as Sox18 and Alpl for Dermal Papilla and Cebpa and Pparg for Preadipocytes (Fig 3G). Overall, our data revealed that the changes in chromatin landscapes in DFPs between E14 and E18 coincided with the demethylase of H3K27me3 markers within genes required for DFP differentiation. Furthermore, we also found an active, high level of expression for the H3K27me3 specific demethylase—Kdm6b/Jmjd3—in both E14 and E18 fibroblasts (Fig EV2E).

Genetic ablation of histone demethylase Kdm6b/Jmjd3 inhibits DFP differentiation

Previous studies have identified the roles of Ezh2 and PRC2 complex in controlling epidermal differentiation by adding the H3K27me3 marker at specific genes to maintain the stemness of the progenitor cells. In contrast, the H3K27me3 demethylase Kdm6b/Jmjd3 has also been shown to be critical in epidermal stem cell differentiation, where ablation of Jmjd3 leads to inhibited differentiation of this cell type (Sen *et al*, 2008; Ezhkova *et al*, 2009). Based on the significant decrease in H3K27me3 markers of DFPs from E14 to E18, we hypothesize that Kdm6b/Jmjd3 demethylase activity is required for the differentiation of dermal fibroblasts. To test the role of Kdm6b/Jmdj3 in dermal differentiation, we generated a transgenic dermal-specific knockout of Jmjd3 mouse model driven by activation of Dermo1 in dermal fibroblasts (Fig 4A). The Dermo1Cre-Kdm6b$^{fl/fl}$ (Kdm6b-cKO) mice are neonatally lethal. Consequently, we focused our studies on the changes that occur between E14.5 and E18.5. Histological analysis of E14.5 skin showed similar stages of skin development between Kdm6b-cKO skin and the WT control where a thin layer of the dermis was found in conjunction with the formation of dermal condensates (Fig 4B). However, at E18.5, we observed drastic changes as Kdm6b-cKO skin appeared to be underdeveloped with smaller hair follicles and significantly thinner dermal layers (Fig 4C and D). We also found that the development of one differentiated cell type—Dermal Papilla—was also inhibited, with fewer Dermal Papilla per hair follicle (Fig 4E). Flow cytometry staining for CD133/Prom1, a surface marker of Dermal Papilla, confirmed a significant 20% decrease in their number from whole dermal preparation (Fig 4F).

To extensively characterize the changes induced by Jmjd3 ablation, we performed a scRNA-seq experiment comparing the E18.5 dermis of Kdm6b-cKO mice to the littermate genetic control (Fig 5A). After preprocessing and filtering steps, 12,947 fibroblasts were retrieved for downstream analysis (Fig 5B). PAGA embedding of single-cell clusters was performed to visualize the trajectory and continuous transition among different fibroblast cell types. In addition, we performed cluster classification as in Fig 1D–F (Figs 5B and EV3A–C). Interestingly, we did not observe a significant change in the number of Dermal Papilla between WT (Kdm6b$^{fl/fl}$) and Kdm6b-cKO. Instead, we found that there was a dramatic reduction in number of preadipocytes detected in Kdm6b-cKO skin compared to the WT control (1,081 in WT, 238 in Kdm6b-cKO), along with a large decrease in the number of lymphatic vasculatures (Figs 5C and D, and EV3A and B). This observation coincides with previous studies reporting the relationship between dermal fibroblasts, dermal preadipocytes, and endothelial vasculature niche (François *et al*, 2008; Tang *et al*, 2008; Gupta *et al*, 2012; Jiang *et al*, 2014; Martinez-Corral *et al*, 2015; Pichol-Thievend *et al*, 2018; Stone & Stainier, 2019; Oliver *et al*, 2020). We next performed a differential gene expression analysis comparing the two conditions using Diffxpy, a more optimized package for statistical testing of single-cell

 © 2023 The Authors

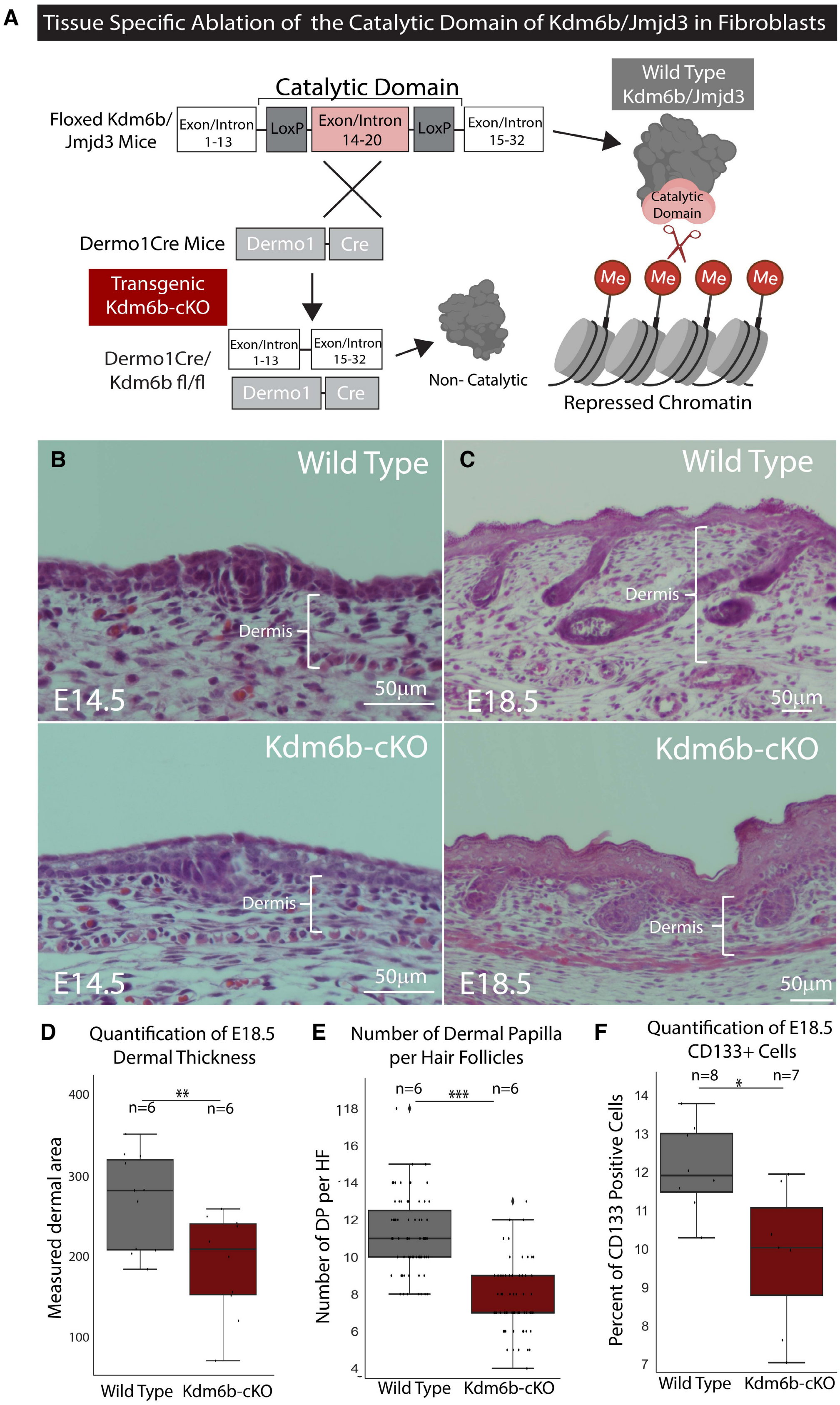
A
Tissue Specific Ablation of the Catalytic Domain of Kdm6b/Jmjd3 in Fibroblasts
Catalytic Domain
Floxed Kdm6b/ Jmjd3 Mice
Exon/Intron 1-13
LoxP
Exon/Intron 14-20
LoxP
Exon/Intron 15-32
Wild Type Kdm6b/Jmjd3
Catalytic Domain
Dermo1Cre Mice
Dermo1
Cre
Transgenic Kdm6b-cKO
Me
Dermo1Cre/ Kdm6b fl/fl
Exon/Intron 1-13
Exon/Intron 15-32
Dermo1
Cre
Non- Catalytic
Repressed Chromatin
B
Wild Type
Dermis
E14.5
50μm
C
Wild Type
Dermis
E18.5
50μm
Kdm6b-cKO
Dermis
E14.5
50μm
Kdm6b-cKO
Dermis
E18.5
50μm
D
Quantification of E18.5 Dermal Thickness
Measured dermal area
n=6
n=6
**
Wild Type
Kdm6b-cKO
E
Number of Dermal Papilla per Hair Follicles
Number of DP per HF
n=6
***
n=6
Wild Type
Kdm6b-cKO
F
Quantification of E18.5 CD133+ Cells
Percent of CD133 Positive Cells
n=8
*
n=7
Wild Type
Kdm6b-cKO

Figure 4.

Figure 4. Kdm6b/Jmjd3 regulates DFP differentiation.

A Transgenic approach to specifically knock-out Jmjd3 in murine model.

B, C H&E staining for histological analysis of E14.5 and E18.5 WT and Kdm6b-cKO skin.

D Quantification of E18.5 thickness comparing E18.5 WT and Kdm6b-cKO skin (*P*-value < 0.01).

E Changes in number of Dermal Papilla per Hair Follicle between WT and Kdm6b-cKO.

F Quantification of flow cytometry comparing the percentages of CD133 positive cells between E18.5 WT and Kdm6b-cKO dermal prep.

Source data are available online for this figure.

data (Materials and Methods). We found that there are 472 genes downregulated in Kdm6b-cKO fibroblasts compared to WT. GO analysis of this list revealed that the differences in gene expression were related to the formation and differentiation of the Preadipocytes population, which revealed that biological processes such as Lipid metabolic process, Brown fat cell differentiation, Fatty acid metabolic process, and White fat cell differentiation were significantly enriched (Figs 5E and EV3D–I). These differences were driven by the significant changes in the number of Preadipocytes between the two conditions, so genes such as Fabp4, Adipoq are significantly downregulated in Kdm6b-cKO fibroblasts (Fig 5F and G). In summary, our histological and scRNA-seq analyses revealed that the ablation of Kdm6b/Jmdj3 demethylase activity in the dermis inhibited the differentiation of DFPs to specific fibroblasts cell fates, while the transient states of Papillary and Reticular were less impacted.

Kdm6b regulates the epigenetic programming of DFP differentiation into functional dermal papilla

Despite the significant impact on the development of the dermis and Dermal Papilla, scRNA-seq data of Kdm6b-cKO skin did not capture large changes in genes associated with dermal papilla function. We hypothesize that the dermal papilla transcriptomic profile reflects the specialized niche environment and epidermal signals, and that Kdm6b/Jmjd3 ablation will alter the intrinsic H3K27me3 epigenetic programs of Kdm6b-cKO fibroblasts. To investigate the altered epigenetic profile, we performed H3K27me3 ChIP-seq comparing E18.5 WT and Kdm6b-cKO fibroblasts (Fig 6A). We found that E18.5 Kdm6b-cKO fibroblasts have an increase in the number and level of H3K27me3 peaks (Fig 6B and C). Compared to the average of 26,228 peaks called in E18.5 WT, Kdm6b-cKO fibroblasts had a large increase of peaks to 42,331 H3K27me3 peaks (Fig 6C). Visualizations of H3K27me3 peaks using heatmaps (Fig 6B) and coverage plots across the whole genome (Fig EV4D) highlighted the apparent increase in H3K27me3 repressive peaks of the same regions in Kdm6b-cKO fibroblasts, since the majority of called peaks overlapped between WT and Kdm6b-cKO (Fig EV4A). We next performed differential peaks analysis using DiffBind to identify significant changes between the two conditions, which resulted in 1916 Kdm6b-cKO peaks compared to 29 peaks in WT fibroblasts (Fig EV4B). GO analysis of the Kdm6b-cKO revealed significantly increased peaks for enrichment of biological processes such as Potassium ion transmembrane transport, Signal

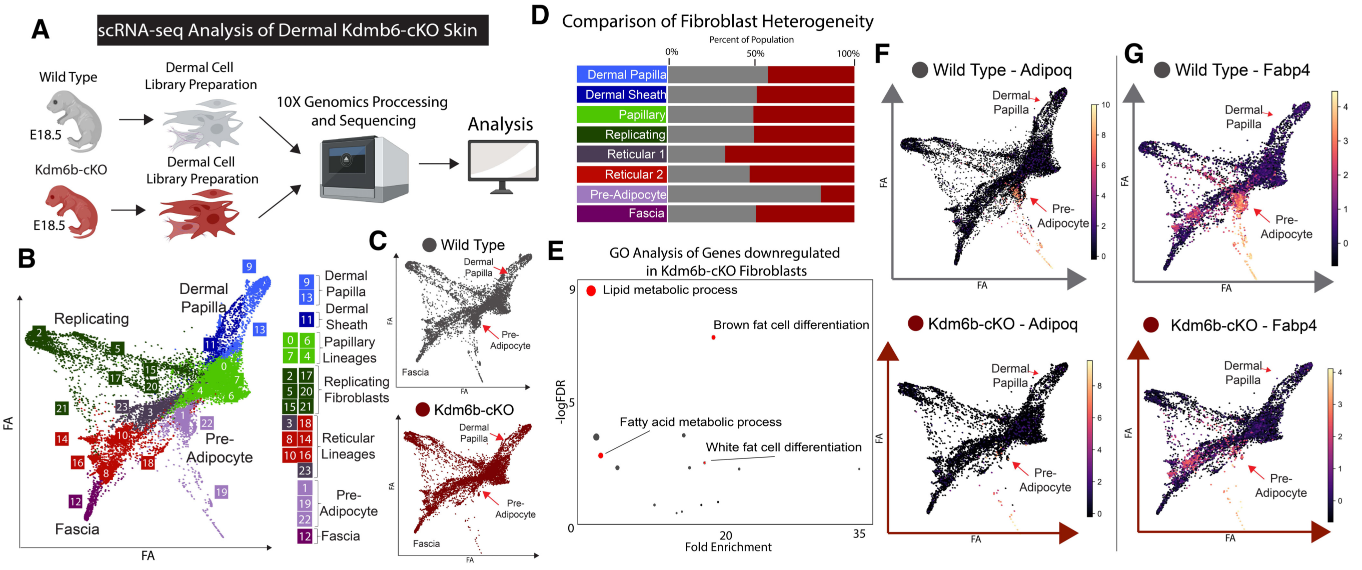

Figure 5. scRNA-seq analysis of Wild Type and Kdmb6b-cKO mouse fibroblasts.

A Experimental strategy to generate single-cell RNA-seq data from E18.5 WT and Kdm6b-cKO mice.

B, C PAGA embedded plots showing cell clusters of integrated E18.5 WT and Kdm6b-cKO fibroblasts.

D Bar plots showing the contribution of each condition (WT and Kdm6b-cKO) to each fibroblast sub-cell types.

E GO analysis of Genes significantly downregulated in Kdm6b-cKO fibroblasts.

F, G PAGA plots showing the expression levels of Adipogenic genes AdipoQ and Fabp4 between WT and Kdm6b-cKO.

Source data are available online for this figure.

© 2023 The Authors

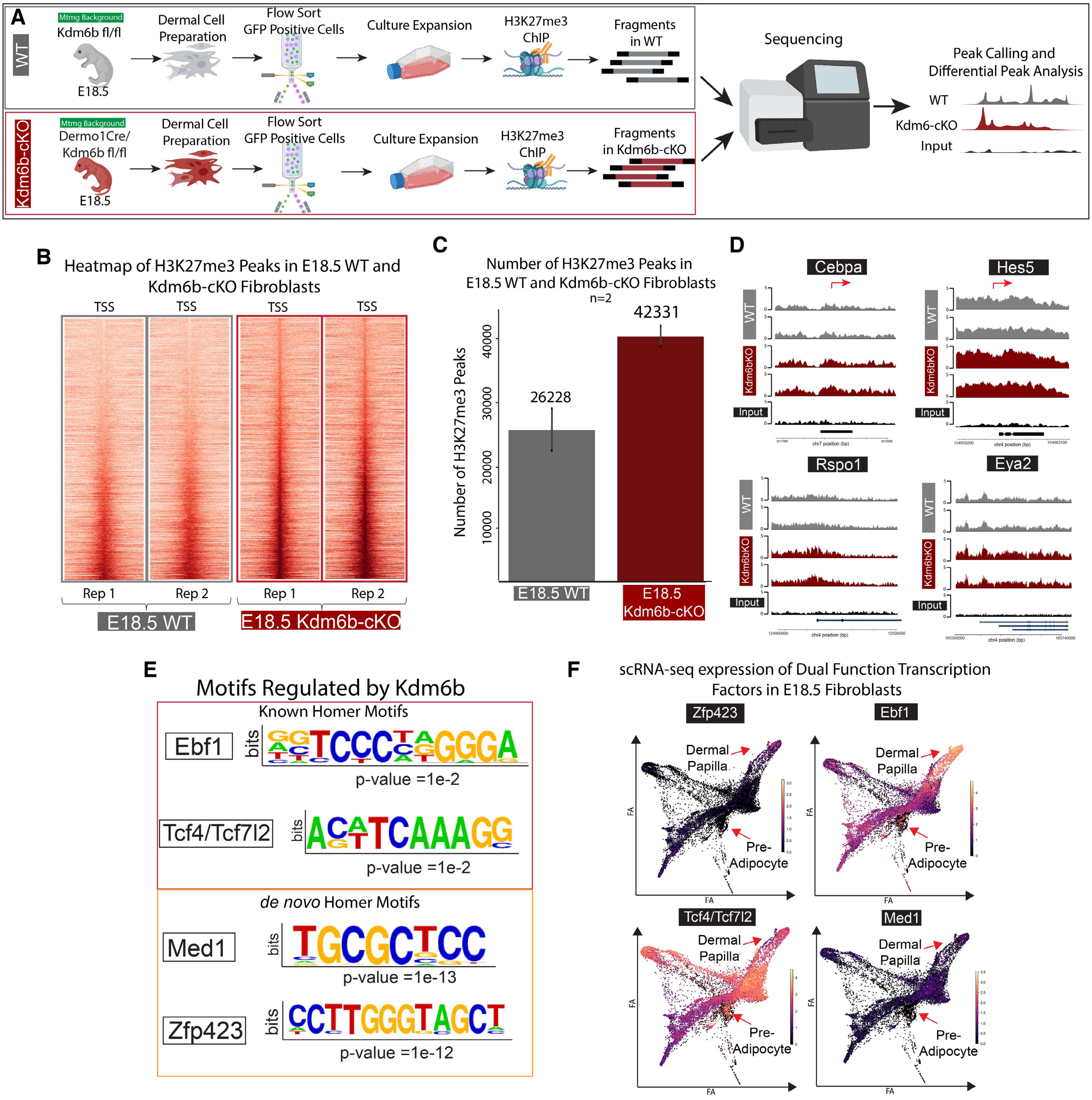

Figure 6. Kdm6b regulates epigenetic de-repression of dual-function Adipogenic and Dermal Papilla transcription factors.

A Experimental design for H3K27me3 ChIP-seq comparing E18.5 WT and Kdm6b-cKO.

B Heatmaps depicting the changes in H3K27me3 peaks in Kdm6b-cKO fibroblasts.

C Bar plots showing the number of H3K27me3 peaks called in E18.5 WT and Kdm6b-cKO Fibroblasts.

D Track plots of relevant transcription factors loci for Dermal Papilla and Preadipocyte lineage commitment (Cebpa, Rspo1, Eya2, and Hes5).

E Homer motif analysis of significantly repressed peaks in Kdm6b-cKO fibroblasts.

F scRNA-seq expression levels of Motifs regulated by Kdm6b activity.

Source data are available online for this figure.

transduction, Cell differentiation, Multicellular organism development, Cell–cell signaling, and Neuron differentiation (Fig EV4C). We did not observe processes directly related to the skin, hair follicle, and dermal white adipocyte differentiation being enriched, even in the present of important genes for Dermal Papilla and Preadipocyte such as Cebpa, Hes5, Rspo1, and Eya2 (Fig 6E; Sennett *et al*, 2015; Mok

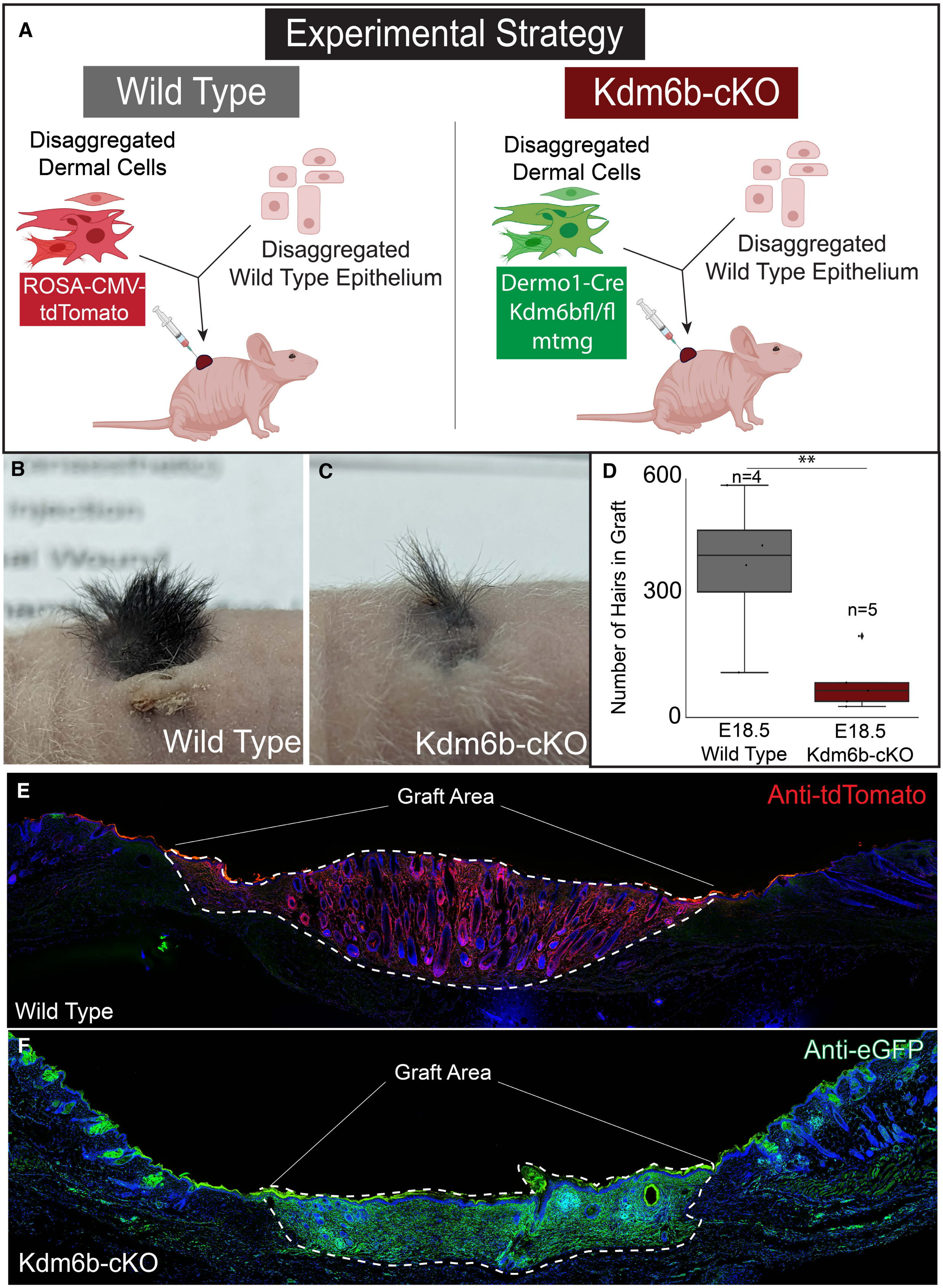

Figure 7.

© 2023 The Authors

Figure 7. Kdm6b regulates DFP differentiation into fully functional Dermal Papilla.

A Experimental strategy for skin reconstitution assay (chamber grafting assay) to analyze the hair forming potential of E18.5 Fibroblast of WT and Kdm6ab-cKO dermis.
B, C Images of hair follicle formations within grafted area comparing WT and Kdm6b-cKO Fibroblasts.
D Quantification of hair follicles reformed in chamber grafting assays.
E, F Immunofluorescence analysis of WT and Kdm6b-cKO grafted area to verify contribution to skin reconstitution.

Source data are available online for this figure.

et al, 2019). When comparing with the transcriptomic changes induced by Kdm6b ablation, we found an overlap of 38 genes between genes downregulated in Kdm6b-cKO scRNA-seq and increase in ChIP-seq data, with many of them have been shown to be important in the development of Dermal Papilla (Fig EV4E). Our results confirmed that genes associated with dermal papilla function are regulated by Kdm6b/Jmjd3 demethylase at the epigenetic level, while other epigenetic modulators could compensate for Jmdj3 function.

To assess the role of epigenetic regulation in Dermal Papilla functions, we hypothesized that the ablation of Kdm6b/Jmjd3 in DFPs will lead to inaccessibility of transcription factor binding sites that regulate fibroblast lineage functions. To address this hypothesis, we utilized the motif finder Homer to screen for binding motif enrichment in the significant peaks regulated by Kdm6b (Fig 6F; Heinz *et al*, 2010). The known Homer motifs revealed an enrichment of Ebf1, Tcf4/Tcf7l2-binding motifs in peaks that were increased in Kdm6b-cKO fibroblasts, while the *de novo* Homer motifs detected the significant enrichment of dermal adipocyte differentiation transcription factors Med1 and Zfp423 (Fig 6F; Gupta *et al*, 2010, 2012; Harms *et al*, 2015; Shao *et al*, 2016; Biferali *et al*, 2021). Interestingly, the expression of these transcription factors was not specific to just Dermal Papilla or Preadipocyte, but these were expressed throughout fibroblasts populations (Fig 6G). This result suggested that they might have distinct roles in both Adipogenesis and Dermal Papilla functions.

Since Kdm6b-cKO mice are neonatally lethal, we performed a chamber grafting assay to evaluate the postnatal potential of the mutant dermis to forming functional dermal papilla that support hair reformation. In order to confirm the contribution of mutant dermis within the grafting assay, we generated a triple transgenic mouse line that harbors the dermal-specific Kdm6b deletion with the mTmG mouse line (Fig 7A). Wild-type cell dermal populations were tracked utilizing the mTmG mouse line. Our analysis of the chamber grafting experiment after 28 days post surgery revealed that hair follicle reformation was robust when wild-type E18.5 dermis was used to seed the dermal component of the graft (Fig 7B and D). However, when E18.5 Kdm6b-cKO dermis was used to seed the chamber grafts (Fig 7C and D) hair follicle reformation was almost ablated despite significant contribution to the graft (Fig 7E and F). Overall, we concluded that DFPs differentiation into distinct cell fates and lineages was programmed by the establishment of unique open chromatin profiles, which is mediated by Kdm6b demethylase activity removing H3K27me3 markers as specific, functional genes loci.

Discussion

Defining the epigenetic state of a dermal fibroblast

Recent studies have demonstrated that stem cells require extensive chromatin accessibility priming prior to lineage commitment (Buenrostro *et al*, 2018; Ranzoni *et al*, 2021). The accessible chromatin profiles of dermal fibroblasts and other cell types could reflect their potential to differentiate into a terminal fate (Li *et al*, 2017; Adam *et al*, 2018; Ma *et al*, 2020; Kim *et al*, 2022; Thompson *et al*, 2022). Here, we discovered that DFPs possess inaccessible chromatin profiles at differentiation specific genes required for lineage commitment. The inaccessible and unprogrammed chromatin profiles of E14.5 DFPs compared to the differentiating fibroblasts helped explain the inability of E14.5 dermis to support HF reformation in the chamber grafting assay (Figs 1 and 2D). Moreover, our scATAC-seq data confirmed the bifurcation of distinct fibroblast lineages during embryonic development (Sorrell & Caplan, 2004; Driskell *et al*, 2013). We found that Upper DFPs possessed the most stem-like chromatin profiles with the least number of accessible peaks. Papillary and Dermal Papilla are closely associated with each other, where significant differential peak analysis and integration with RNA velocity driver genes showed the large overlapping regions between them (Figs 2F and EV1D). In addition, our integrative RNA velocity and scATAC-seq analysis revealed a stepwise arrangement of accessible chromatin for DFP differentiation, where Dermal Papilla possess the most unique chromatin architecture, but genes specific for Papillary fibroblasts also remain accessible in the Dermal Papilla (Fig 2F). This result confirmed our previous finding of fibroblasts states and fates, with the Dermal Papilla and Preadipocytes being two defined terminal fates for DFP differentiation (Thompson *et al*, 2022).

Our investigation of the regulation of chromatin architecture by epigenetic histone modifications revealed that DFP differentiation occurs through specific derepression of chromatin to support fibroblast lineage function. We found that DFPs required a significant decrease in H3K27me3 levels in genes associated with differentiating fibroblasts functions such as Sox18, Alpl, Cebpa, and Pparg. Notably, during the differentiation process, we only observed a small increase in H3K27me3 markers (24 peaks) (Fig 3B–F). Our result suggested that the opening of chromatin accessibility profiles is correlated with the demethylation of H3K27me3 from E14.5 and during fibroblast lineage commitment. This finding confirms the epigenetic regulation of stem cell differentiation where the PRC2 complex is needed to trimethylate H3K27 and repress genes required for cellular differentiation (Boyer *et al*, 2006; Lee *et al*, 2006; Ezhkova *et al*, 2009; Wang *et al*, 2010; Thulabandu *et al*, 2018). Our model suggests that after Ezh2 establishes an epigenetic profile of early dermal fibroblast progenitors that requires Kdm6b/Jmjd3 activity to initiate chromatin derepression at differentiation genes.

Mechanisms regulating adipogenesis and dermal papilla lineage commitment

Lineage tracing and single-cell studies have demonstrated the importance of fibroblast lineages commitment of DFPs in skin development

© 2023 The Authors

and homeostasis (Driskell & Watt, 2015; Griffin *et al*, 2020; Plikus *et al*, 2021). Genetic ablation of Jmdj3/Kmd6b in dermal fibroblasts revealed an inhibition of DFPs to differentiate into terminal fates such as functional Dermal Papilla and Adipocytes. Interestingly, the fibroblast states of papillary and reticular lineages appeared to be unaffected. We found that even though hair follicle morphogenesis was initiated and that Dermal Papilla were observed, Kdm6b-cKO hair follicles appeared stunted and underdeveloped (Fig 4C). Our single-cell RNA-seq experiment comparing WT and Kdm6b-cKO skin revealed a major decrease of Preadipocytes in Kdm6b-cKObut not Dermal Papilla (Fig 4I). It is possible that the existence of other epigenetic regulators and histone marks such as bivalency with H3K4me3, or the acetylation of H3K27me3, may regulate the formation but not the function of Dermal Papilla. Our scRNA-seq comparing WT and Kdm6b-cKO skin also detected the significant upregulation of Chromatin Modification GO process, indicating a compensation mechanism to remodel and program the chromatin profile bypassing H3K27me3 demethylation (Fig EV4D and E). Our H3K27me3 ChIP-seq evaluating E18.5 WT and Kdm6b-cKOfibroblasts validated the ablation of Kdm6b/Jmdj3 which led to the increase in H3K27me3 levels across the genome. Motif analysis of significantly increased H3K27me3 peaks in Kdm6b-cKOfibroblasts revealed an array of transcription factors that might possess dual-functions. These transcription factors such as Ebf1, Zfp423, Tcf7l2 are not distinctly expressed across fibroblast lineages, even though they have been shown to play important roles in adipogenesis and Dermal Papilla function (Fig EV5A–D; Gupta *et al*, 2010; Festa *et al*, 2011; Lien *et al*, 2014). After the integration of scATAC-seq data with our ChIPseq data, we discovered that these transcription factors might regulate different sets of downstream genes in different cell types (Fig EV5B). Overall, our data suggested that the chromatin accessibility profiles can dictate the potential of fibroblast commitment to the terminal fates, and that Kdm6b/Jmdj3 demethylation regulates this process. The differential activity of Kdm6b/Jmjd3 in diverse fibroblast lineages acts as a mechanism to regulate the dual-function of non-unique transcription factors. Finally, we have also reconstructed a high-resolution molecular blueprint of dermal fibroblast differentiation processes across multiple modalities including transcriptomic, accessible chromatin profiles, and epigenetic markers to define DFPs and distinct fibroblast fates, freely accessible at our webtool.

scATAC-seq provides insights into the regulation of progenitor cell differentiation and adult tissue homeostasis

Recent studies have characterized the single-cell chromatin profiles of fibroblasts in murine skin wound healing. In regular scarring wounds, Foster *et al* (2021) found different populations of fibroblasts with distinct putative roles (Foster *et al*, 2021). While comparing large WIHN and small scarring wounds, another study defined the distinct chromatin landscapes of regenerative and scarring fibroblasts (Abbasi *et al*, 2020), although the competent regenerative fibroblasts might arise from the periphery regenerative fibroblasts (Phan *et al*, 2021). Biernaskie and colleagues have also discovered the inaccessible inflammatory regulome in reindeer fibroblasts which could lead to the regenerative states found in reindeer's velvet (Sinha *et al*, 2022). Altogether, these studies indicated that the dynamic cellular state of dermal fibroblasts is defined by accessible chromatin profiles, and that manipulation of distinct regulatory mechanisms to achieve a regenerative fibroblast state might hold the key to promoting scarless wound healing. Alternatively, there may not be an advantage for achieving a 'stem-like' fibroblast state but to utilize and achieve optimal populations of different committed fibroblast populations that arise from their distinct lineages. Our current study provides an in-depth analysis looking at the changes in chromatin architecture happening during embryonic development while establishing the ground state of accessible chromatin, transcriptomic, and epigenetic profiles for DFPs and differentiating fibroblasts.

In the context of aging, dermal fibroblasts have been shown to have lost their cellular identity at transcriptomic and epigenetic levels (López-Otín *et al*, 2013; Salzer *et al*, 2018; Yang *et al*, 2023). Future studies will require the identification of the homeostatic chromatin states in different fibroblast populations to activate fibroblasts to resist the effect of chronic aging. Our molecular blueprint will provide the reference points of differentiating fibroblasts in embryogenesis and postnatal dermis to help determine the chromatin profiles of how fibroblast heterogeneity was established.

Materials and Methods

Reagent Tools table

Reagent/Resource	Reference or Source	Identifier or Catalog Number
**Experimental models**		
C57BL/6J (M. musculus)	Jackson Lab	RRID:IMSR_JAX:000664
Twist2-Cre C67BL/6J (M. musculus)	Jackson Lab	B6.129X1; RRID: IMSR_JAX:008712
Kdm6bf/f (M. musculus)	Jackson Lab	B6.Cg; RRID: IMSR_JAX:029615
**Antibodies**		
Rabbit anti RFP	Rockland/ThermoFisher	600-401-379
Rat anti Itga6	BD Biosciences	555735
Rabbit anti H3K27me3	Active Motif	AB_2561020
Rat anti CD133	ThermoFisher	H34477
Rabbit anti Lef1	Cell Signaling	#2230
Sheep anti MKi67	R&D systems	AF7649
Goat anti Sox2	R&D systems	AF2018

© 2023 The Authors

Reagents and Tools table (continued)

Reagent/Resource	Reference or Source	Identifier or Catalog Number
Rabbit anti CEBPa	Cell Signaling	#8178
Chicken anti GFP	Rockland/ThermoFisher	600-901-215
**Sequencing reagents**		
Chromium Next GEM Single Cell ATAC kit v2	10× Genomics	1000406
Chromium Next GEM Single Cell 3' Kit v3.1	10× Genomics	1000269
SimpleChIP Enzymatic Chromatin IP Kit (Magnetic Beads)	Cell Signaling	9003
**Chemicals, enzymes and other reagents**		
Dispase	Corning	CB-40235
Trypsin	Gibco	25200114
**Software**		
scanpy 1.9.1	https://scanpy.readthedocs.io/en/stable/	
scVelo 0.2.5	https://scvelo.readthedocs.io/en/stable/	
Seurat 4.1.1	https://satijalab.org/seurat/	
Signac 1.8.0	https://stuartlab.org/signac/	
Scanorama	https://github.com/brianhie/scanorama	
DiffBind 3.6.3	https://bioconductor.org/packages/release/bioc/html/DiffBind.html	
ChIPSeeker 1.32.0	https://guangchuangyu.github.io/software/ChIPseeker/	
HOMER 4.11	http://homer.ucsd.edu/homer/	
cellranger 6.0.0	https://www.10xgenomics.com/support	
cellranger-atac 2.1.0	https://www.10xgenomics.com/support	
MACS2 2.2.7.1	https://pypi.org/project/MACS2/	
bowtie2	https://github.com/BenLangmead/bowtie2	
scikit-learn 1.2.2	https://scikit-learn.org/stable/	
**Other**		
Illumina NovaSeq 600	Illumina	
Illumina HiSeq 4000	Illumina	

Methods and Protocols

Mouse models

All animal procedures in this study were in accordance with protocols approved by Washington State University Institutional Animal Care and Use Committee ASAF #6723, #6724, #6726. Mice were derived from C57BL/6 background. The following transgenic mouse lines were used in this study: Twist2-Cre (RRID:MGI:3840442), Kdm6b$^{fl/fl}$ (RRID:IMSR_JAX:029615), ROSA26$^{mT/mG}$ (RRID: IMSR_JAX:037456).

Single-cell suspension digestion

Tissues were collected from embryonic E14.5, E18.5, and P5 mice skins. Cells isolation procedure described previously was followed to obtain single-cell suspension from tissues (Jensen *et al*, 2010).

Histological analysis

Tissues were collected from mice back skins. For formalin-fixed paraffin-embedded, tissues were fixed with 4% paraformaldehyde in 1× PBS overnight. Hematoxylin and Eosin staining were done with 5 μm-thick sections. Colored images were taken with Nikon E600 and Nikon DS-Fi3 camera. Horizontal wholemount preparation was done as previously published (Salz & Driskell, 2017). Immunofluorescent images were taken with Leica SP8 confocal microscope at 20× objective. Analysis and quantification of H&E images were given unique ID and scored by a blinded researcher.

Chamber allografting assay

This procedure was performed as previously described (Jensen *et al*, 2010). A 8 × 10^6 WT neonatal epidermal cells were combined with 10^6 dermal cells from different timepoints or from different transgenic lines. Combination of cells were injected into silicone chambers grafted on 6 mm circular dorsal wounds in Foxn1$^{-/-}$ mice. Collection of grafted areas were done 28 days post-surgery.

Single-cell gene expression

Single-cell suspensions from E14.5, E18.5, P5 were processed separately for 10× Genomics Single Cell 3' Gene Expression (v2) PN-120237, targeting 10,000 cells per library. Libraries were sequenced on Illumina HiSeq 4000 (100 bp Paired-End). Fastq files were aligned to mm10 reference genome using CellRanger version 6.0.0.

Single-cell suspensions of E18.5 WT and Kdm6b-cKO dermal preparations were processed separately for 10× Genomics Single Cell 3' Gene Expression kit (V3.1) PN-1000128, targeting 10,000 cells per

library (3 mice each, pooled into 1 library for each condition). Libraries were sequenced on Illumina NovaSeq6000 (100 Paired-End). Fastq files were aligned to mm10 reference genome using CellRanger version 6.0.0. Loom files were generated from CellRanger alignment outputs via velocyto packages for RNA velocity analysis (La Manno *et al*, 2018). Analysis of scRNA-seq data were done using Scanpy and ScVelo (Wolf *et al*, 2019; Bergen *et al*, 2020).

Low-quality and doublet cells were filtered by larger than 5,000 genes or less than 700 genes, less than 10 percent mitochondrial genes. Data were normalized to 10,000 reads per cells, log transformed, then scaled to maximum standard deviation 10. Integration and batch-correction by Time were done using Scanorama (Hie *et al*, 2019). Differential gene expression was calculated using Diffxpy (https://github.com/theislab/diffxpy).

Partition-based graph abstraction (PAGA) was used to infer the trajectory of continuous cell transitions while preserving the global topology of data (Wolf *et al*, 2019). RNA velocity analysis was done using dynamical model to retrieve latent time (Bergen *et al*, 2020).

For the time points scRNA-seq in Fig 1, we aimed to investigate the differentiation trajectories of dermal progenitor fibroblasts across three different time points that were sequenced separately. To establish the continuous transition states of fibroblasts among these development time points, Scanorama was used to integrate and correct for batch effect. We next utilized PAGA to provide.

All codes used to analyze this data is available on our github page.

Single-cell assay for transposase-accessible chromatin

Dermal cells were collected and digested into the single cell suspension from 3 individual mouse embryos pooled together for each sample. Nuclei isolation from single cell suspension were prepared targeting 10,000 nuclei per library. Isolated nuclei were processed using 10× Genomics Single Cell ATAC v2 (CG000496). Libraries were sequenced on Illumina NovaSeq 6000 (100 bp Paired-end). Fastq files were aligned to mm10 reference genome using Cellranger-atac version 2.1.0. Cellranger outputs including Peaks called, Fragments, and metadata were used for downstream analysis with Signac in R (Stuart *et al*, 2021). Basic filtering and preprocessing were done screening for Peak region fragment < 100,000 and Percent reads in peaks > 40%. Clustering analysis was done using latent semantic indexing (LSI) (Cusanovich *et al*, 2015), dimension reduction was carried out by UMAP (McInnes *et al*, 2018). scRNA-seq integration with scATAC-seq data for cell type classification was done by Signac Label Transferred methods using scRNA-seq fibroblasts object and scATAC-seq fibroblast object (Stuart *et al*, 2021).To improve the peaks calling sensitivity and accuracy, we performed Peak Call within Signac using MACS2 function (Zhang *et al*, 2008). Differential accessible peaks between clusters were calculated using FindMarkers() function within Signac. Motif binding sites analysis of specific cell type was done using MotifMatchR (https://greenleaflab.github.io/motifmatchr/).

To generate the peaks heatmap in Fig 2, we performed the differential peaks analysis to isolate the list of peaks associated with distinct fibroblast clusters in scATAC-seq data in Signac. Within each cluster, we filtered and retained the list of peaks that are associated with genes found in the velocity driver genes of the same cell type from scRNA-seq analysis.

Codes used to analyze this data is available on our github page.

Chromatin immunoprecipitation sequencing (ChIP-Seq)

Dermal preparation of E14.5, E18.5 WT; E18.5 WT and Kdm6b-cKO murine skins (n = 2 for each sample) were expanded in culture to reach 2×10^7 cells in AmnioMAX complete medium. ChIP assay was done using SimpleChIP Plus Enzymatic Chromatin IP kit (magnetic beads) #9005 from CellSignalling and H3K27me3 antibody (Active Motif RRID:AB_2561020). ChIP samples were prepared with Kapa Hyper prep kit. Libraries were sequenced on Illumina NovaSeq 6000 (100 bp Paired-End). Fastq ChIP-seq files were aligned using bowtie2 and converted to sorted bam files and BED files. E18.5 WT and Kdm6b-cKO samples were subsampled to equivalent read depths as other samples (6×10^7 reads per sample). Peak called was done using MACS2 with --broad option and –broad-cutoff q-value = 0.01. Visualization of ChIP-seq data was done using ChIPseeker for CoveragePlot (Wang *et al*, 2022), Easeq for Tracksplot (Lerdrup & Hansen, 2020). Differential ChIP-seq calculation was done using DiffBind (Ross-Innes *et al*, 2012; Stark & Brown, 2016).

Motif Analysis was done using HOMER findMotifs function (Heinz *et al*, 2010).

Flow cytometry

E18.5 single-cell suspension from WT and Kdm6bcKO mice were stained with primary antibody and secondary antibody for 1 h each with 3× PBS washes in between and after. Flow sorting of cells were done on Sony SH800 Flow Sorter. Flow analysis were done on ThermoFisher Attune Flow Cytometer.

Statistical analysis

Histology data comparing two groups were tested for normality using Shapiro–Wilk, equal distribution using Levene, and t-test for independent samples if assumptions are met, otherwise we used Wilcoxon Rank Sum test. Single-cell RNA and ATAC data statistical tests were done using nonparametric Wilcoxon Rank Sum test.

Data availability

We have uploaded the sequencing data to NCBI GEO-datasets. The files can be found according to the following GSE numbers: GSE227257 (http://www.ncbi.nlm.nih.gov/geo/query/acc.cgi?acc=GSE227257) – scRNA-seq of E14.5, E18.5, and P5 murine skin; GSE227262 (http://www.ncbi.nlm.nih.gov/geo/query/acc.cgi?acc=GSE227262) – H3K27me3 ChIP-seq of E14.5 and E18.5 murine dermis; GSE233161 (http://www.ncbi.nlm.nih.gov/geo/query/acc.cgi?acc=GSE233161) – scATAC-seq of E14.5 and E18.5 murine skin; GSE227256 (http://www.ncbi.nlm.nih.gov/geo/query/acc.cgi?acc=GSE227256) – scRNA-seq of E18.5 WT and Kdm6b-cKO murine skin; GSE227262 (https://www.ncbi.nlm.nih.gov/geo/query/acc.cgi?acc=GSE226567) – H3K27me3 ChIP-seq of E18.5 WT and Kdm6b-cKO murine skin.

Expanded View for this article is available online.

Acknowledgements

This work was supported by NIH National Institute of Arthritis and Musculoskeletal and Skin Diseases (NIAMS) R01 AR078743. We would also like to acknowledge the Franceschi Microscopy & Imaging Center at Washington

© 2023 The Authors

State University; Center for Reproductive Biology (Melissa Oatley); Gene Editing Reagent Core at Washington State University (Lisette Maddison, Deqiang Miao); Washington State University Functional Genomics Initiative; This publication includes data generated at the UC San Diego IGM Genomics Center utilizing an Illumina NovaSeq 6000 that was purchased with funding from a National Institutes of Health SIG grant (#S10 OD026929); Genomics & Cell Characterization Core Facility (GC3F) at University of Oregon; Laboratory for Biotechnology and Bioanalysis at Washington State University (Derek Pouchnik, Mark Wildung, and Weiwei Du), QMP, JM was supported by a National Institute of General Medicine Sciences training grant (T32GM008336), QMP was supported by Poncin Fellowship.

Author contributions

Quan M Phan: Conceptualization; resources; software; supervision; funding acquisition; validation; methodology; writing – original draft; writing – review and editing. **Lucia Salz:** Conceptualization; methodology. **Sam S Kindl:** Resources; software; validation. **Jayden S Lopez:** Resources. **Sean M Thompson:** Writing – review and editing. **Jasson Makkar:** Writing – review and editing. **Iwona M Driskell:** Conceptualization; supervision; validation; methodology; writing – review and editing. **Ryan R Driskell:** Conceptualization; resources; software; supervision; funding acquisition; validation; methodology; writing – original draft.

Disclosure and competing interests statement

The authors declare that they have no conflict of interest.

References

Abbasi S, Sinha S, Labit E, Rosin NL, Yoon G, Rahmani W, Jaffer A, Sharma N, Hagner A, Shah P *et al* (2020) Distinct regulatory programs control the latent regenerative potential of dermal fibroblasts during wound healing. *Cell Stem Cell* 27: 396 – 412.e6

Adam RC, Yang H, Ge Y, Lien W-H, Wang P, Zhao Y, Polak L, Levorse J, Baksh SC, Zheng D *et al* (2018) Temporal layering of signaling effectors drives chromatin remodeling during hair follicle stem cell lineage progression. *Cell Stem Cell* 22: 398 – 413.e7

Agger K, Cloos PAC, Christensen J, Pasini D, Rose S, Rappsilber J, Issaeva I, Canaani E, Salcini AE, Helin K (2007) UTX and JMJD3 are histone H3K27 demethylases involved in HOX gene regulation and development. *Nature* 449: 731 – 734

Atit R, Sgaier SK, Mohamed OA, Taketo MM, Dufort D, Joyner AL, Niswander L, Conlon RA (2006) β-catenin activation is necessary and sufficient to specify the dorsal dermal fate in the mouse. *Dev Biol* 296: 164 – 176

Barile M, Imaz-Rosshandler I, Inzani I, Ghazanfar S, Nichols J, Marioni JC, Guibentif C, Göttgens B (2021) Coordinated changes in gene expression kinetics underlie both mouse and human erythroid maturation. *Genome Biol* 22: 197

Bergen V, Lange M, Peidli S, Wolf FA, Theis FJ (2020) Generalizing RNA velocity to transient cell states through dynamical modeling. *Nat Biotechnol* 38: 1408 – 1414

Bergen V, Soldatov RA, Kharchenko PV, Theis FJ (2021) RNA velocity—current challenges and future perspectives. *Mol Syst Biol* 17: e10282

Biferali B, Bianconi V, Perez DF, Kronawitter SP, Marullo F, Maggio R, Santini T, Polverino F, Biagioni S, Summa V *et al* (2021) Prdm16-mediated H3K9 methylation controls fibro-adipogenic progenitors identity during skeletal muscle repair. *Sci Adv* 7: eabd9371

Biggs LC, Mäkelä OJ, Myllymäki S-M, Das Roy R, Närhi K, Pispa J, Mustonen T, Mikkola ML (2018) Hair follicle dermal condensation forms via Fgf20 primed cell cycle exit, cell motility, and aggregation. *eLife* 7: e36468

Boyer LA, Plath K, Zeitlinger J, Brambrink T, Medeiros LA, Lee TI, Levine SS, Wernig M, Tajonar A, Ray MK *et al* (2006) Polycomb complexes repress developmental regulators in murine embryonic stem cells. *Nature* 441: 349 – 353

Buechler MB, Pradhan RN, Krishnamurty AT, Cox C, Calviello AK, Wang AW, Yang YA, Tam L, Caothien R, Roose-Girma M *et al* (2021) Cross-tissue organization of the fibroblast lineage. *Nature* 593: 575 – 579

Buenrostro JD, Giresi PG, Zaba LC, Chang HY, Greenleaf WJ (2013) Transposition of native chromatin for fast and sensitive epigenomic profiling of open chromatin, DNA-binding proteins and nucleosome position. *Nat Methods* 10: 1213 – 1218

Buenrostro JD, Wu B, Litzenburger UM, Ruff D, Gonzales ML, Snyder MP, Chang HY, Greenleaf WJ (2015) Single-cell chromatin accessibility reveals principles of regulatory variation. *Nature* 523: 486 – 490

Buenrostro JD, Corces MR, Lareau CA, Wu B, Schep AN, Aryee MJ, Majeti R, Chang HY, Greenleaf WJ (2018) Integrated single-cell analysis maps the continuous regulatory landscape of human hematopoietic differentiation. *Cell* 173: 1535 – 1548.e16

Cao R, Wang L, Wang H, Xia L, Erdjument-Bromage H, Tempst P, Jones RS, Zhang Y (2002) Role of histone H3 lysine 27 methylation in polycomb-group silencing. *Science* 298: 1039 – 1043

Cao J, Packer JS, Ramani V, Cusanovich DA, Huynh C, Daza R, Qiu X, Lee C, Furlan SN, Steemers FJ *et al* (2017) Comprehensive single-cell transcriptional profiling of a multicellular organism. *Science* 357: 661 – 667

Chen D, Jarrell A, Guo C, Lang R, Atit R (2012) Dermal β-catenin activity in response to epidermal Wnt ligands is required for fibroblast proliferation and hair follicle initiation. *Development* 139: 1522 – 1533

Chen JS, Longaker MT, Gurtner GC (2013) Murine models of human wound healing. In *Wound regeneration and repair: methods and protocols, methods in molecular biology*, Gourdie RG, Myers TA (eds), pp 265 – 274. Totowa, NJ: Humana Press

Cole MA, Quan T, Voorhees JJ, Fisher GJ (2018) Extracellular matrix regulation of fibroblast function: redefining our perspective on skin aging. *J Cell Commun Signal* 12: 35 – 43

Correa-Gallegos D, Jiang D, Christ S, Ramesh P, Ye H, Wannemacher J, Kalgudde Gopal S, Yu Q, Aichler M, Walch A *et al* (2019) Patch repair of deep wounds by mobilized fascia. *Nature* 576: 287 – 292

Cusanovich DA, Daza R, Adey A, Pliner HA, Christiansen L, Gunderson KL, Steemers FJ, Trapnell C, Shendure J (2015) Multiplex single-cell profiling of chromatin accessibility by combinatorial cellular indexing. *Science* 348: 910 – 914

Cusanovich DA, Hill AJ, Aghamirzaie D, Daza RM, Pliner HA, Berletch JB, Filippova GN, Huang X, Christiansen L, DeWitt WS *et al* (2018) A single-cell atlas of *in vivo* mammalian chromatin accessibility. *Cell* 174: 1309 – 1324.e18

Dhouailly D (1973) Dermo-epidermal interactions between birds and mammals: differentiation of cutaneous appendages. *Development* 30: 587 – 603

Dhouailly D, Sengel P (1975) Feather- and hair-forming properties of dermal cells of glabrous skin from bird and mammals. *C R Acad Hebd Seances Acad Sci D* 281: 1007 – 1010

Driskell RR, Watt FM (2015) Understanding fibroblast heterogeneity in the skin. *Trends Cell Biol* 25: 92 – 99

Driskell RR, Lichtenberger BM, Hoste E, Kretzschmar K, Simons BD, Charalambous M, Ferron SR, Herault Y, Pavlovic G, Ferguson-Smith AC

et al (2013) Distinct fibroblast lineages determine dermal architecture in skin development and repair. *Nature* 504: 277 – 281

Driskell RR, Jahoda CAB, Chuong C-M, Watt FM, Horsley V (2014) Defining dermal adipose tissue. *Exp Dermatol* 23: 629 – 631

Ezhkova E, Pasolli HA, Parker JS, Stokes N, Su I, Hannon G, Tarakhovsky A, Fuchs E (2009) Ezh2 orchestrates gene expression for the stepwise differentiation of tissue-specific stem cells. *Cell* 136: 1122 – 1135

Fernandes KJL, McKenzie IA, Mill P, Smith KM, Akhavan M, Barnabé-Heider F, Biernaskie J, Junek A, Kobayashi NR, Toma JG *et al* (2004) A dermal niche for multipotent adult skin-derived precursor cells. *Nat Cell Biol* 6: 1082 – 1093

Festa E, Fretz J, Berry R, Schmidt B, Rodeheffer M, Horowitz M, Horsley V (2011) Adipocyte lineage cells contribute to the skin stem cell niche to drive hair cycling. *Cell* 146: 761 – 771

Flora P, Ezhkova E (2020) Regulatory mechanisms governing epidermal stem cell function during development and homeostasis. *Development* 147: dev194100

Foster DS, Januszyk M, Yost KE, Chinta MS, Gulati GS, Nguyen AT, Burcham AR, Salhotra A, Ransom RC, Henn D *et al* (2021) Integrated spatial multiomics reveals fibroblast fate during tissue repair. *Proc Natl Acad Sci USA* 118: e2110025118

François M, Caprini A, Hosking B, Orsenigo F, Wilhelm D, Browne C, Paavonen K, Karnezis T, Shayan R, Downes M *et al* (2008) Sox18 induces development of the lymphatic vasculature in mice. *Nature* 456: 643 – 647

Fu J, Hsu W (2013) Epidermal Wnt controls hair follicle induction by orchestrating dynamic signaling crosstalk between the epidermis and dermis. *J Invest Dermatol* 133: 890 – 898

Griffin MF, des Jardins-Park HE, Mascharak S, Borrelli MR, Longaker MT (2020) Understanding the impact of fibroblast heterogeneity on skin fibrosis. *Dis Model Mech* 13: dmm044164

Gruber F, Kremslehner C, Eckhart L, Tschachler E (2020) Cell aging and cellular senescence in skin aging—recent advances in fibroblast and keratinocyte biology. *Exp Gerontol* 130: 110780

Guerrero-Juarez CF, Dedhia PH, Jin S, Ruiz-Vega R, Ma D, Liu Y, Yamaga K, Shestova O, Gay DL, Yang Z *et al* (2019) Single-cell analysis reveals fibroblast heterogeneity and myeloid-derived adipocyte progenitors in murine skin wounds. *Nat Commun* 10: 650

Gupta RK, Arany Z, Seale P, Mepani RJ, Ye L, Conroe HM, Roby YA, Kulaga H, Reed RR, Spiegelman BM (2010) Transcriptional control of preadipocyte determination by Zfp423. *Nature* 464: 619 – 623

Gupta RK, Mepani RJ, Kleiner S, Lo JC, Khandekar MJ, Cohen P, Frontini A, Bhowmick DC, Ye L, Cinti S *et al* (2012) Zfp423 expression identifies committed preadipocytes and localizes to adipose endothelial and perivascular cells. *Cell Metab* 15: 230 – 239

Gupta K, Levinsohn J, Linderman G, Chen D, Sun TY, Dong D, Taketo MM, Bosenberg M, Kluger Y, Choate K *et al* (2019) Single-cell analysis reveals a hair follicle dermal niche molecular differentiation trajectory that begins prior to morphogenesis. *Dev Cell* 48: 17 – 31.e6

Gurtner GC, Werner S, Barrandon Y, Longaker MT (2008) Wound repair and regeneration. *Nature* 453: 314 – 321

Harms MJ, Lim H-W, Ho Y, Shapira SN, Ishibashi J, Rajakumari S, Steger DJ, Lazar MA, Won K-J, Seale P (2015) PRDM16 binds MED1 and controls chromatin architecture to determine a brown fat transcriptional program. *Genes Dev* 29: 298 – 307

Heintzman ND, Hon GC, Hawkins RD, Kheradpour P, Stark A, Harp LF, Ye Z, Lee LK, Stuart RK, Ching CW *et al* (2009) Histone modifications at human enhancers reflect global cell-type-specific gene expression. *Nature* 459: 108 – 112

Heinz S, Benner C, Spann N, Bertolino E, Lin YC, Laslo P, Cheng JX, Murre C, Singh H, Glass CK (2010) Simple combinations of lineage-determining transcription factors prime cis-regulatory elements required for macrophage and B cell identities. *Mol Cell* 38: 576 – 589

Hie B, Bryson B, Berger B (2019) Efficient integration of heterogeneous single-cell transcriptomes using Scanorama. *Nat Biotechnol* 37: 685 – 691

Jacob T, Annusver K, Czarnewski P, Dalessandri T, Kastriti ME, Levron CL, Mikkola ML, Rendl M, Lichtenberger BM, Donati G *et al* (2022) Molecular and spatial design of early skin development. *bioRxiv* https://doi.org/10.1101/2022.12.28.522081 [PREPRINT]

Jensen KB, Driskell RR, Watt FM (2010) Assaying proliferation and differentiation capacity of stem cells using disaggregated adult mouse epidermis. *Nat Protoc* 5: 898 – 911

Jiang Y, Berry DC, Tang W, Graff JM (2014) Independent stem cell lineages regulate adipose organogenesis and adipose homeostasis. *Cell Rep* 9: 1007 – 1022

Jiang D, Correa-Gallegos D, Christ S, Stefanska A, Liu J, Ramesh P, Rajendran V, De Santis MM, Wagner DE, Rinkevich Y (2018) Two succeeding fibroblastic lineages drive dermal development and the transition from regeneration to scarring. *Nat Cell Biol* 20: 422 – 431

Joost S, Annusver K, Jacob T, Sun X, Dalessandri T, Sivan U, Sequeira I, Sandberg R, Kasper M (2020) The molecular anatomy of mouse skin during hair growth and rest. *Cell Stem Cell* 26: 441 – 457.e7

Kang S, Chovatiya G, Tumbar T (2019) Epigenetic control in skin development, homeostasis and injury repair. *Exp Dermatol* 28: 453 – 463

Kang S, Long K, Wang S, Sada A, Tumbar T (2020) Histone H3 K4/9/27 trimethylation levels affect wound healing and stem cell dynamics in adult skin. *Stem Cell Reports* 14: 34 – 48

Karlić R, Chung H-R, Lasserre J, Vlahoviček K, Vingron M (2010) Histone modification levels are predictive for gene expression. *Proc Natl Acad Sci USA* 107: 2926 – 2931

Kim JY, Park M, Ohn J, Seong RH, Chung JH, Kim KH, Jo SJ, Kwon O (2022) Twist2-driven chromatin remodeling governs the postnatal maturation of dermal fibroblasts. *Cell Rep* 39: 110821

Kishimoto J, Burgeson RE, Morgan BA (2000) Wnt signaling maintains the hair-inducing activity of the dermal papilla. *Genes Dev* 14: 1181 – 1185

La Manno G, Soldatov R, Zeisel A, Braun E, Hochgerner H, Petukhov V, Lidschreiber K, Kastriti ME, Lönnerberg P, Furlan A *et al* (2018) RNA velocity of single cells. *Nature* 560: 494 – 498

Lee TI, Jenner RG, Boyer LA, Guenther MG, Levine SS, Kumar RM, Chevalier B, Johnstone SE, Cole MF, Isono K *et al* (2006) Control of developmental regulators by polycomb in human embryonic stem cells. *Cell* 125: 301 – 313

Lee J, Kang S, Lilja KC, Colletier KJ, Scheitz CJF, Zhang YV, Tumbar T (2016) Signalling couples hair follicle stem cell quiescence with reduced histone H3 K4/K9/K27me3 for proper tissue homeostasis. *Nat Commun* 7: 11278

Lerdrup M, Hansen K (2020) User-friendly and interactive analysis of ChIP-seq data using EaSeq. *Methods Mol Biol* 2117: 35 – 63

Li D, Liu J, Yang X, Zhou C, Guo J, Wu C, Qin Y, Guo L, He J, Yu S *et al* (2017) Chromatin accessibility dynamics during iPSC reprogramming. *Cell Stem Cell* 21: 819 – 833.e6

Lien W-H, Polak L, Lin M, Lay K, Zheng D, Fuchs E (2014) *In vivo* transcriptional governance of hair follicle stem cells by canonical Wnt regulators. *Nat Cell Biol* 16: 179 – 190

López-Otín C, Blasco MA, Partridge L, Serrano M, Kroemer G (2013) The hallmarks of aging. *Cell* 153: 1194 – 1217

 © 2023 The Authors

Ma S, Zhang B, LaFave LM, Earl AS, Chiang Z, Hu Y, Ding J, Brack A, Kartha VK, Tay T *et al* (2020) Chromatin potential identified by shared single-cell profiling of RNA and chromatin. *Cell* 183: 1103 – 1116.e20

Martinez-Corral I, Ulvmar MH, Stanczuk L, Tatin F, Kizhatil K, John SWM, Alitalo K, Ortega S, Makinen T (2015) Nonvenous origin of dermal lymphatic vasculature. *Circ Res* 116: 1649 – 1654

Mascharak S, des Jardins-Park HE, Davitt MF, Griffin M, Borrelli MR, Moore AL, Chen K, Duoto B, Chinta M, Foster DS *et al* (2021) Preventing engrailed-1 activation in fibroblasts yields wound regeneration without scarring. *Science* 372: eaba2374

McInnes L, Healy J, Melville J (2018) UMAP: uniform manifold approximation and projection. *J Open Source Softw* 3: 861

Millar SE (2002) Molecular mechanisms regulating hair follicle development. *J Invest Dermatol* 118: 216 – 225

Mok K-W, Saxena N, Heitman N, Grisanti L, Srivastava D, Muraro MJ, Jacob T, Sennett R, Wang Z, Su Y *et al* (2019) Dermal condensate niche fate specification occurs prior to formation and is placode progenitor dependent. *Dev Cell* 48: 32 – 48.e5

Muhl L, Genové G, Leptidis S, Liu J, He L, Mocci G, Sun Y, Gustafsson S, Buyandelger B, Chivukula IV *et al* (2020) Single-cell analysis uncovers fibroblast heterogeneity and criteria for fibroblast and mural cell identification and discrimination. *Nat Commun* 11: 3953

Oliver G, Kipnis J, Randolph GJ, Harvey NL (2020) The lymphatic vasculature in the 21st century: novel functional roles in homeostasis and disease. *Cell* 182: 270 – 296

Phan QM, Fine GM, Salz L, Herrera GG, Wildman B, Driskell IM, Driskell RR (2020) Lef1 expression in fibroblasts maintains developmental potential in adult skin to regenerate wounds. *eLife* 9: e60066

Phan QM, Sinha S, Biernaskie J, Driskell RR (2021) Single-cell transcriptomic analysis of small and large wounds reveals the distinct spatial organization of regenerative fibroblasts. *Exp Dermatol* 30: 92 – 101

Pichol-Thievend C, Betterman KL, Liu X, Ma W, Skoczylas R, Lesieur E, Bos FL, Schulte D, Schulte-Merker S, Hogan BM *et al* (2018) A blood capillary plexus-derived population of progenitor cells contributes to genesis of the dermal lymphatic vasculature during embryonic development. *Development* 145: dev160184

Plikus MV, Guerrero-Juarez CF, Ito M, Li YR, Dedhia PH, Zheng Y, Shao M, Gay DL, Ramos R, Hsi T-C *et al* (2017) Regeneration of fat cells from myofibroblasts during wound healing. *Science* 355: 748 – 752

Plikus MV, Wang X, Sinha S, Forte E, Thompson SM, Herzog EL, Driskell RR, Rosenthal N, Biernaskie J, Horsley V (2021) Fibroblasts: origins, definitions, and functions in health and disease. *Cell* 184: 3852 – 3872

Ranzoni AM, Tangherloni A, Berest I, Riva SG, Myers B, Strzelecka PM, Xu J, Panada E, Mohorianu I, Zaugg JB *et al* (2021) Integrative single-cell RNA-seq and ATAC-seq analysis of human developmental hematopoiesis. *Cell Stem Cell* 28: 472 – 487.e7

Rinkevich Y, Walmsley GG, Hu MS, Maan ZN, Newman AM, Drukker M, Januszyk M, Krampitz GW, Gurtner GC, Lorenz HP *et al* (2015) Identification and isolation of a dermal lineage with intrinsic fibrogenic potential. *Science* 348: aaa2151

Rognoni E, Gomez C, Pisco AO, Rawlins EL, Simons BD, Watt FM, Driskell RR (2016) Inhibition of β-catenin signalling in dermal fibroblasts enhances hair follicle regeneration during wound healing. *Development* 143: 2522 – 2535

Ross-Innes CS, Stark R, Teschendorff AE, Holmes KA, Ali HR, Dunning MJ, Brown GD, Gojis O, Ellis IO, Green AR *et al* (2012) Differential oestrogen receptor binding is associated with clinical outcome in breast cancer. *Nature* 481: 389 – 393

Salz L, Driskell RR (2017) Horizontal whole mount: a novel processing and imaging protocol for thick, three-dimensional tissue cross-sections of skin. *J Vis Exp* e56106

Salzer MC, Lafzi A, Berenguer-Llergo A, Youssif C, Castellanos A, Solanas G, Peixoto FO, Stephan-Otto Attolini C, Prats N, Aguilera M *et al* (2018) Identity noise and adipogenic traits characterize dermal fibroblast aging. *Cell* 175: 1575 – 1590.e22

Sen GL, Webster DE, Barragan DI, Chang HY, Khavari PA (2008) Control of differentiation in a self-renewing mammalian tissue by the histone demethylase JMJD3. *Genes Dev* 22: 1865 – 1870

Sennett R, Wang Z, Rezza A, Grisanti L, Roitershtein N, Sicchio C, Mok KW, Heitman NJ, Clavel C, Ma'ayan A *et al* (2015) An integrated transcriptome atlas of embryonic hair follicle progenitors, their niche, and the developing skin. *Dev Cell* 34: 577 – 591

Shao M, Ishibashi J, Kusminski CM, Wang QA, Hepler C, Vishvanath L, MacPherson KA, Spurgin SB, Sun K, Holland WL *et al* (2016) Zfp423 maintains white adipocyte identity through suppression of the beige cell thermogenic gene program. *Cell Metab* 23: 1167 – 1184

Shook BA, Wasko RR, Mano O, Rutenberg-Schoenberg M, Rudolph MC, Zirak B, Rivera-Gonzalez GC, López-Giráldez F, Zarini S, Rezza A *et al* (2020) Dermal adipocyte lipolysis and myofibroblast conversion are required for efficient skin repair. *Cell Stem Cell* 26: 880 – 895.e6

Singer AJ, Clark RAF (1999) Cutaneous wound healing. *N Engl J Med* 341: 738 – 746

Sinha S, Sparks HD, Labit E, Robbins HN, Gowing K, Jaffer A, Kutluberk E, Arora R, Raredon MSB, Cao L *et al* (2022) Fibroblast inflammatory priming determines regenerative versus fibrotic skin repair in reindeer. *Cell* 185: 4717 – 4736.e25

Sorrell JM, Caplan AI (2004) Fibroblast heterogeneity: more than skin deep. *J Cell Sci* 117: 667 – 675

Stark R, Brown G (2016) *DiffBind: differential binding analysis of ChIP-Seq peak data*. Cambridge, UK: University of Cambridge

Stone OA, Stainier DYR (2019) Paraxial mesoderm is the major source of lymphatic endothelium. *Dev Cell* 50: 247 – 255.e3

Stuart T, Srivastava A, Madad S, Lareau CA, Satija R (2021) Single-cell chromatin state analysis with Signac. *Nat Methods* 18: 1333 – 1341

Tang W, Zeve D, Suh JM, Bosnakovski D, Kyba M, Hammer RE, Tallquist MD, Graff JM (2008) White fat progenitor cells reside in the adipose vasculature. *Science* 322: 583 – 586

Thompson SM, Phan QM, Winuthayanon S, Driskell IM, Driskell RR (2022) Parallel single-cell multiomics analysis of neonatal skin reveals the transitional fibroblast states that restrict differentiation into distinct fates. *J Invest Dermatol* 142: 1812 – 1823.e3

Thulabandu V, Chen D, Atit RP (2018) Dermal fibroblast in cutaneous development and healing. *Wiley Interdiscip Rev Dev Biol* 7: e307

Thulabandu V, Nehila T, Ferguson JW, Atit RP (2021) Dermal EZH2 orchestrates dermal differentiation and epidermal proliferation during murine skin development. *Dev Biol* 478: 25 – 40

Tran TH, Jarrell A, Zentner GE, Welsh A, Brownell I, Scacheri PC, Atit R (2010) Role of canonical Wnt signaling/β-catenin via Dermo1 in cranial dermal cell development. *Development* 137: 3973 – 3984

Trapnell C (2015) Defining cell types and states with single-cell genomics. *Genome Res* 25: 1491 – 1498

Wang L, Jin Q, Lee J-E, Su I, Ge K (2010) Histone H3K27 methyltransferase Ezh2 represses Wnt genes to facilitate adipogenesis. *Proc Natl Acad Sci USA* 107: 7317 – 7322

Wang Q, Li M, Wu T, Zhan L, Li L, Chen M, Xie W, Xie Z, Hu E, Xu S *et al* (2022) Exploring epigenomic datasets by ChIPseeker. *Curr Protoc* 2: e585

© 2023 The Authors

Watt FM, Fujiwara H (2011) Cell-extracellular matrix interactions in normal and diseased skin. *Cold Spring Harb Perspect Biol* 3: a005124

Wojciechowicz K, Gledhill K, Ambler CA, Manning CB, Jahoda CAB (2013) Development of the mouse dermal adipose layer occurs independently of subcutaneous adipose tissue and is marked by restricted early expression of FABP4. *PloS One* 8: e59811

Wolf FA, Hamey FK, Plass M, Solana J, Dahlin JS, Göttgens B, Rajewsky N, Simon L, Theis FJ (2019) PAGA: graph abstraction reconciles clustering with trajectory inference through a topology preserving map of single cells. *Genome Biol* 20: 59

Wong CE, Paratore C, Dours-Zimmermann MT, Rochat A, Pietri T, Suter U, Zimmermann DR, Dufour S, Thiery JP, Meijer D *et al* (2006) Neural crest–derived cells with stem cell features can be traced back to multiple lineages in the adult skin. *J Cell Biol* 175: 1005–1015

Xiang Y, Zhu Z, Han G, Lin H, Xu L, Chen CD (2007) JMJD3 is a histone H3K27 demethylase. *Cell Res* 17: 850–857

Yang J-H, Hayano M, Griffin PT, Amorim JA, Bonkowski MS, Apostolides JK, Salfati EL, Blanchette M, Munding EM, Bhakta M *et al* (2023) Loss of epigenetic information as a cause of mammalian aging. *Cell* 186: 305–326.e27

Zhang Y, Liu T, Meyer CA, Eeckhoute J, Johnson DS, Bernstein BE, Nusbaum C, Myers RM, Brown M, Li W *et al* (2008) Model-based analysis of ChIP-Seq (MACS). *Genome Biol* 9: R137

License: This is an open access article under the terms of the Creative Commons Attribution License, which permits use, distribution and reproduction in any medium, provided the original work is properly cited.

Expanded View Figures

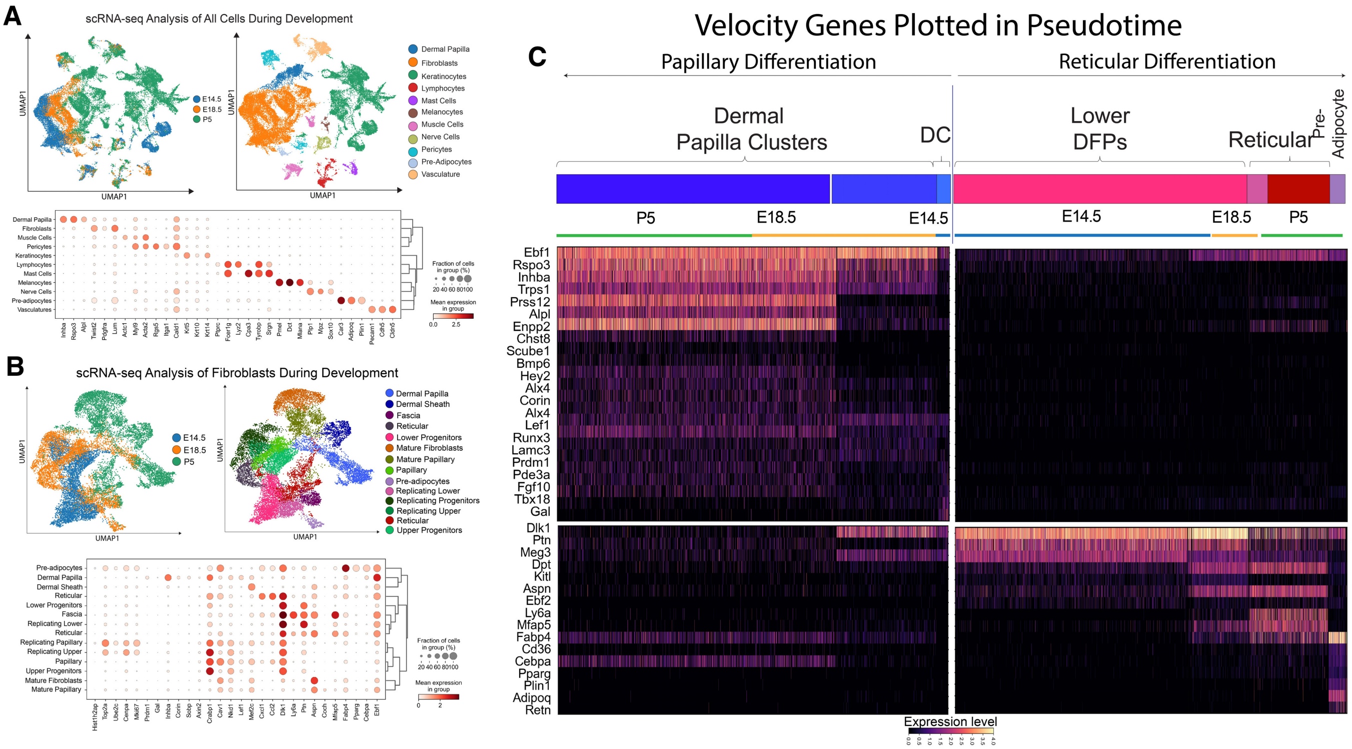

Figure EV1. scRNA-seq analysis.

A scRNA-seq analysis of all cells from E14.5, E18.5, and P5 skin.

B scRNA-seq analysis of fibroblast subset re-clustered from E14.5, E18.5, and P5 skin.

C Pseudotime-heatmap depicting the differentiation projections of Upper Progenitors to Dermal Papilla and Lower Progenitors to Preadipocyte.

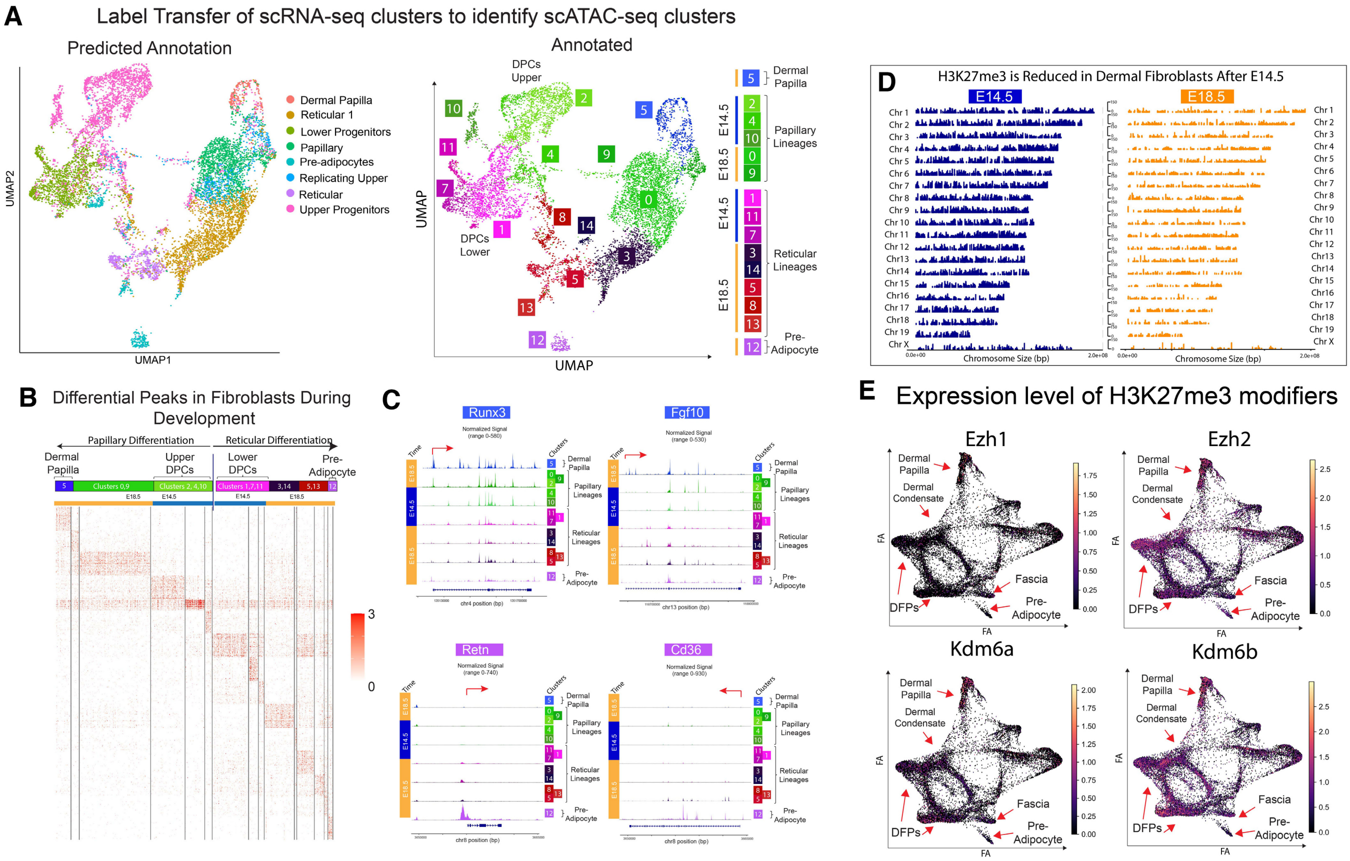

Figure EV2. scATAC-seq analysis and expression of epigenetic H3K27-me3 modifiers in E14.5, E18.5, and P5 fibroblast populations.

A Label transfer experiment of scRNA-seq cluster to identify scATAC-seq cluster in E14.5 and E18.5 skin.
B Differential peak analysis of scATAC-seq clusters arranged in pseudotime.
C Trackplot of fibroblast clusters arranged in pseudotime for Runx3, Fgf10, Retn, and CD36.
D Coverage Plots highlighting the reduction of H3K27me3 from E14.5 to E18.5 dermal fibroblasts.
E Expression of epigenetic modifiers overlayed in scRNA-seq UMAPs of fibroblasts during murine skin development.

© 2023 The Authors

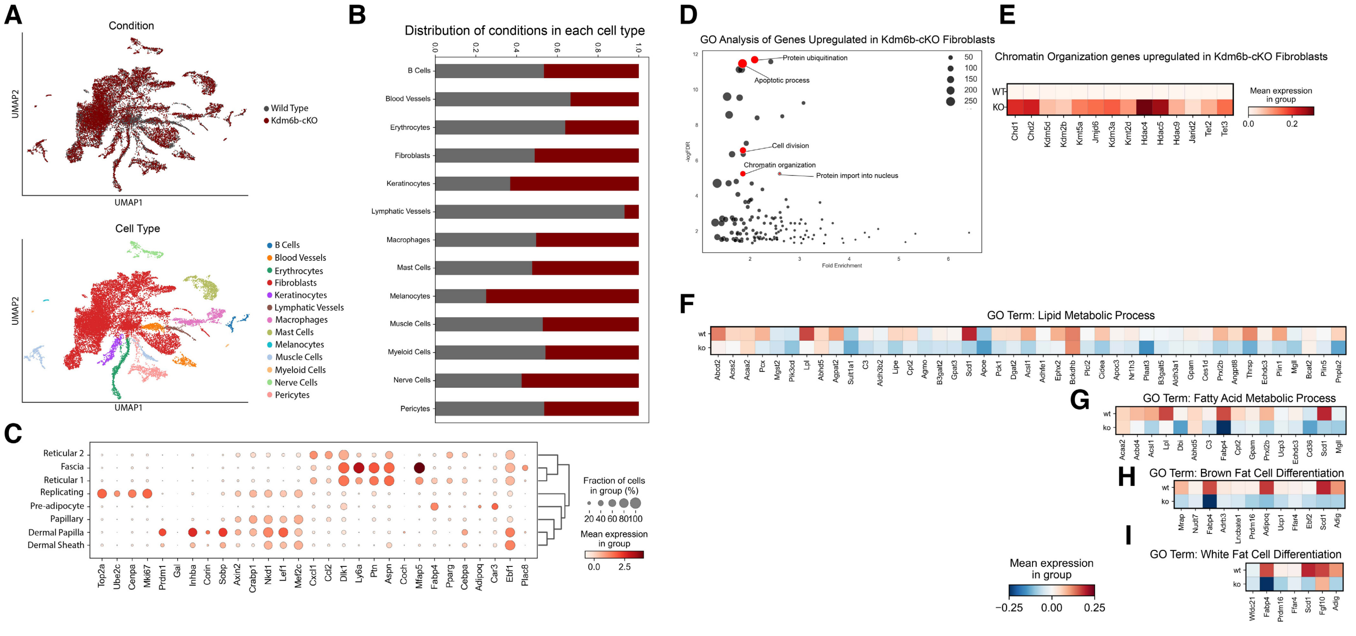

Figure EV3. scRNA-seq analysis of all cells in WT and Dermo1Cre-Kdm6b$^{fl/fl}$ skin and differential expression analysis of genes from GO Analysis of WT and Kdm6b-cKO.

A UMAP projections of WT and Dermo1Cre-Kdm6b$^{fl/fl}$ skin.

B Quantification of the number of cells within each cluster type based on genotype represented as a percentage in the cluster.

C Dotplot of gene utilized to identify cell clusters.

D GO analysis of differentially regulated genes comparing WT and Dermo1Cre-Kdm6b$^{fl/fl}$ skin.

E Gene expression of Chromatin Organization genes from GO Analysis.

F–I Differential expression of GO-Terms genes from (F) Lipid Metabolic Process (G) Fatty Acid Metabolic Process (H) Brown Fat Cell Differentiation and (I) White Fat Cell Differentiation between WT and Kdm6b-cKO fibroblasts.

© 2023 The Authors

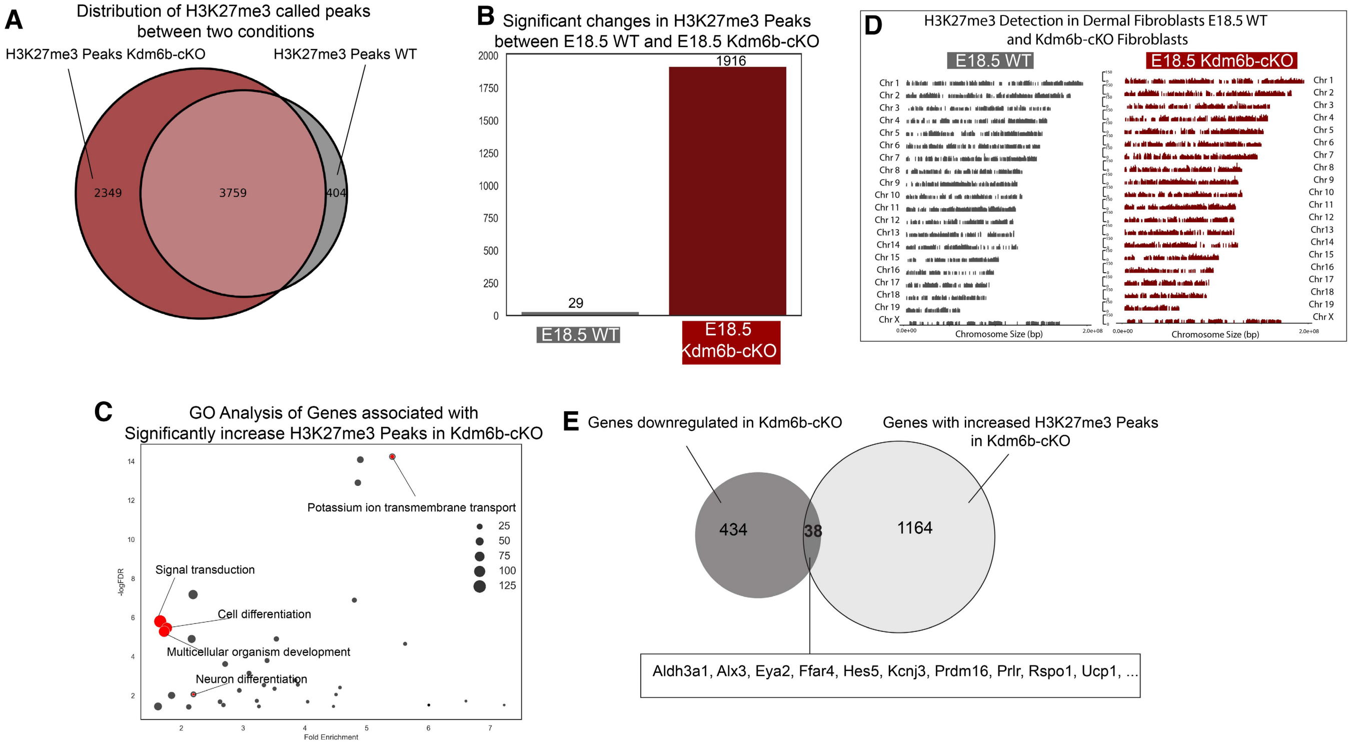

Figure EV4. Integrative analysis of H3K27-me3 peaks between WT and Dermo1Cre-Kdm6b$^{fl/fl}$ fibroblasts with integrated motif analysis in scATAC data and scRNA-seq data.

A Venn diagram comparing H3K27me3 peaks between WT and Dermo1Cre-Kdm6b$^{fl/fl}$ fibroblasts.
B Quantitation of significantly called peaks from WT and Dermo1Cre-Kdm6b$^{fl/fl}$ fibroblasts.
C GO Analysis of peaks significantly increased in Dermo1Cre-Kdm6b$^{fl/fl}$ fibroblasts
D Coverage plots highlighting the increase in H3K27me3 peaks in Kdm6b-cKO.
E Venn diagram comparing downregulated genes in scRNA-seq analysis and peaks in ChIPseq analysis.

 © 2023 The Authors

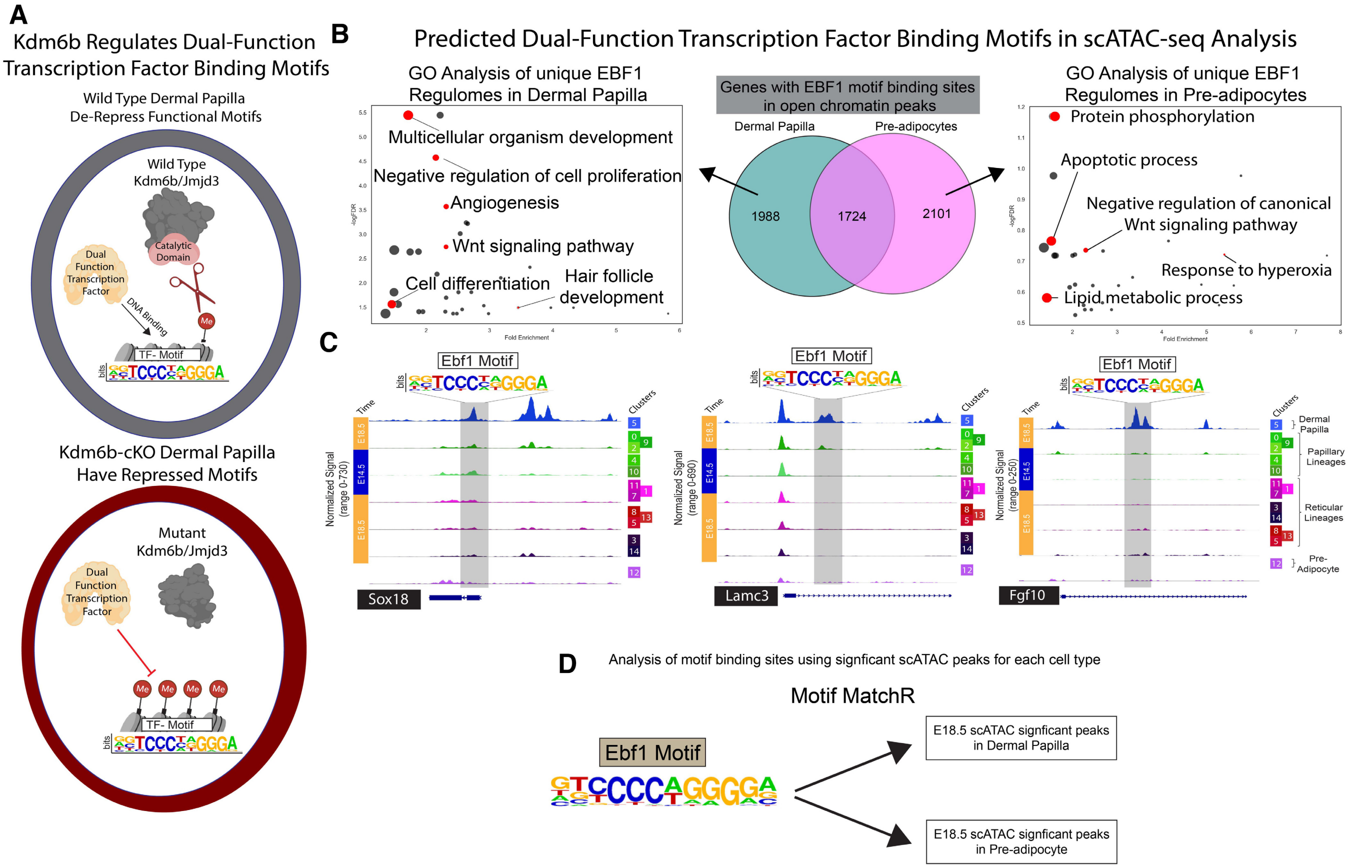

Figure EV5. Kdm6b regulates dual function transcription factor bindings sites.

A Model for Dual-Function Transcription Factor regulation of Dermal Papilla activity regulated by Kdm6b/Jmjd3 de-repression.

B Dual-Function Transcription Factor, Ebf1, binding activities represented by a GO-analysis of Ebf1 motifs in scATAC-seq peaks from Dermal Papilla and Preadipocytes fibroblast populations.

C scATAC-seq track-plot of Dermal Papilla genes, Sox18, Lamc3, and Fgf10. Predicted Ebf1 motifs are highlighted in a gray box on top of the peak that is predicted to have the motif.

D Motif analysis of scATAC peaks for GO analysis.

© 2023 The Authors
