# Supplementary figures and images for: Lineage commitment of dermal fibroblast progenitors is controlled by Kdm6b‐mediated chromatin demethylation
Source: EMBO J. 2023 Aug 21;42(19):e113880. doi: 10.15252/embj.2023113880 (PMC10548174; doi:10.15252/embj.2023113880)

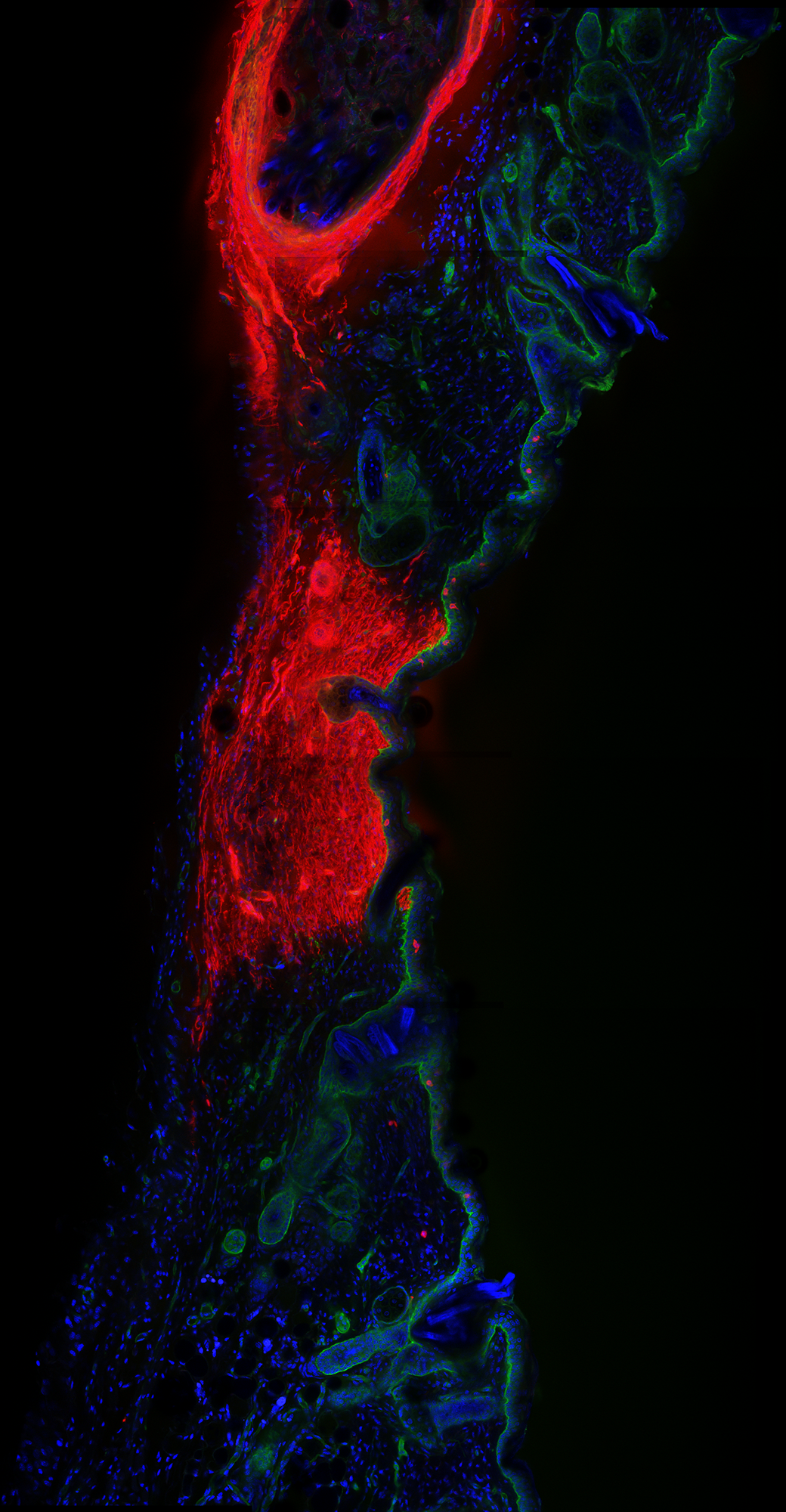

Supplement: Supplementary file 3 — Source Data for Figure 1 [file EMBJ-42-e113880-s009.zip › Fig1/1H/Fig1H_P5.tif]

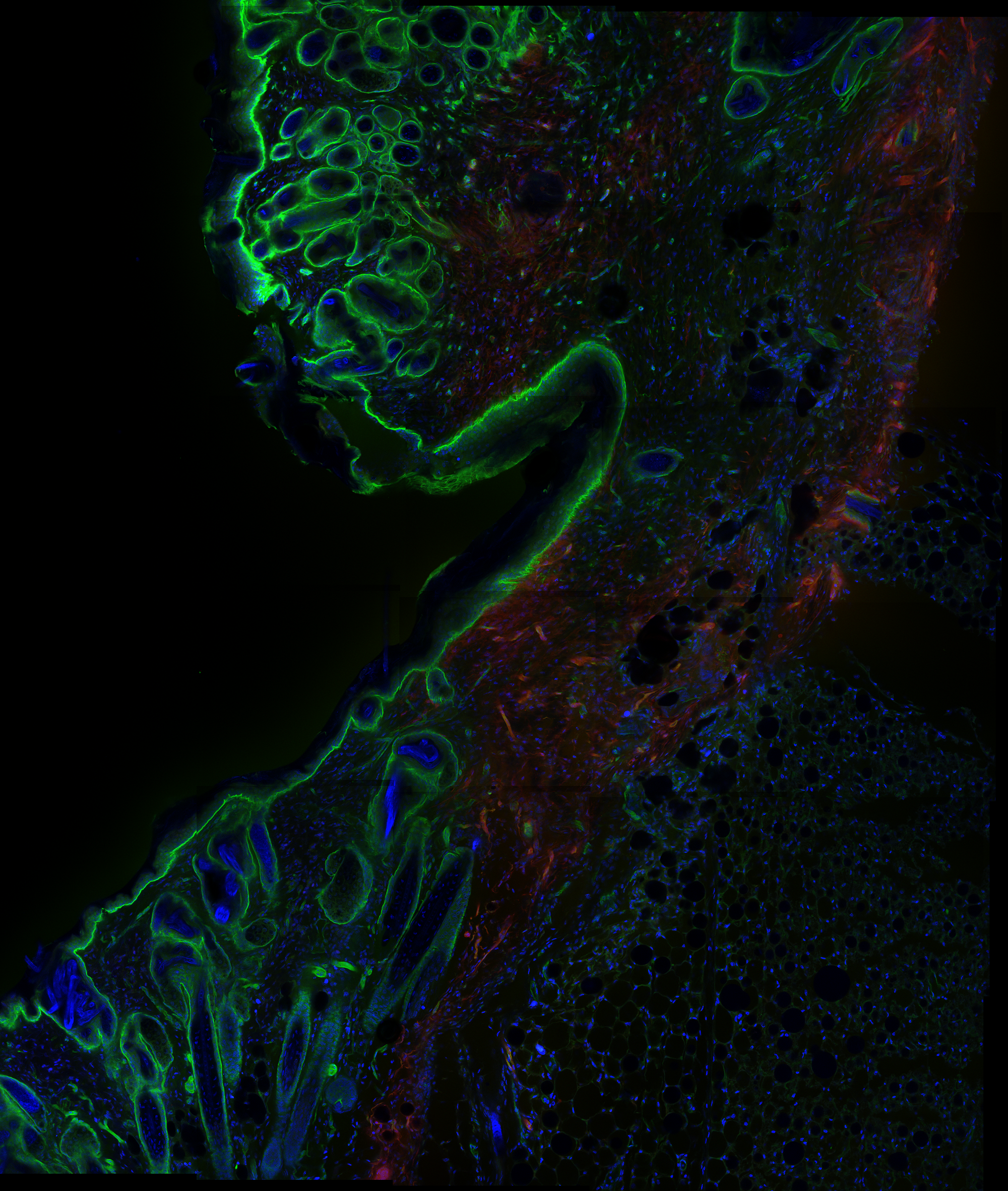

Supplement: Supplementary file 3 — Source Data for Figure 1 [file EMBJ-42-e113880-s009.zip › Fig1/1H/Fig1H_E18.tif]

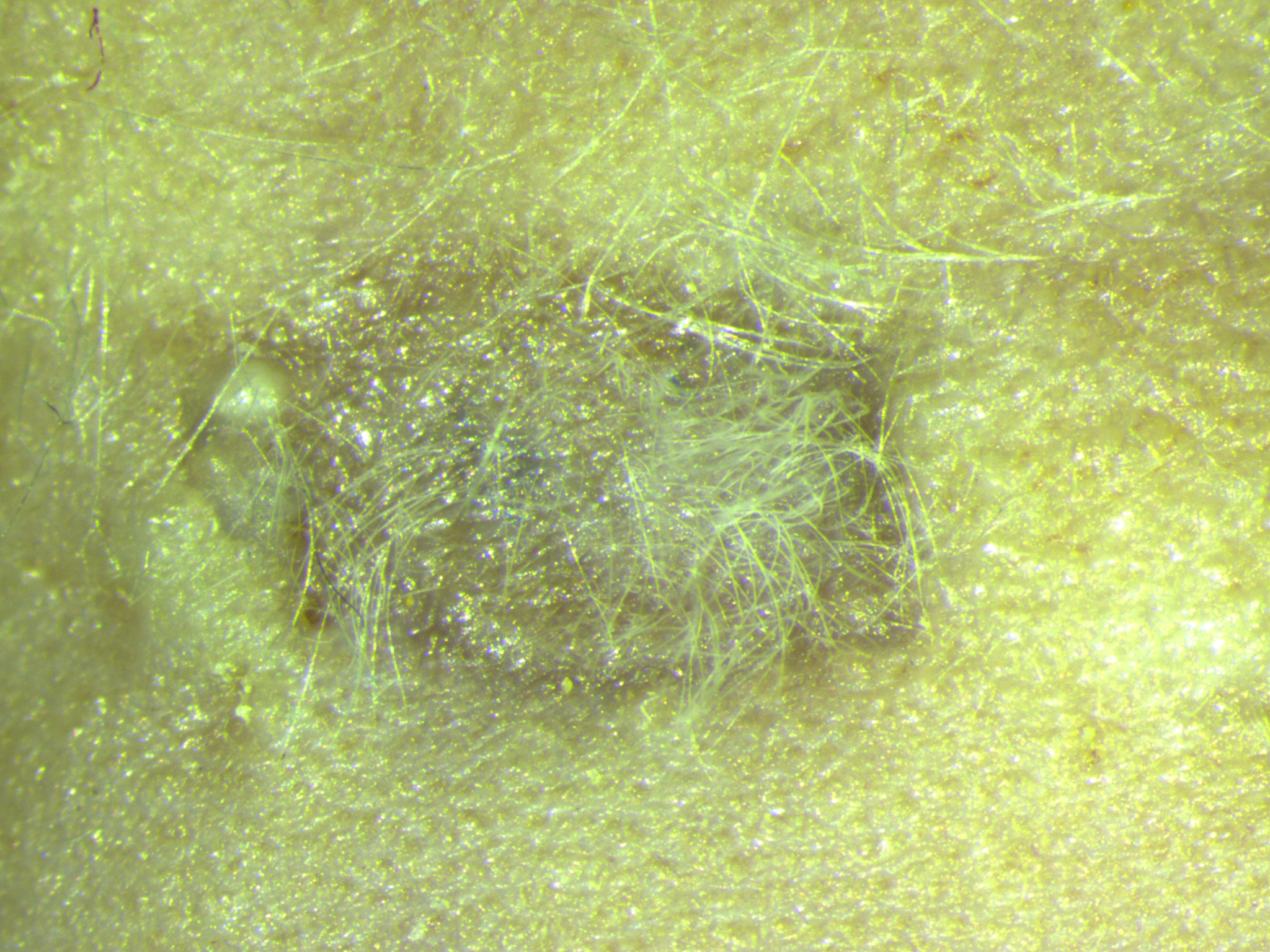

Supplement: Supplementary file 3 — Source Data for Figure 1 [file EMBJ-42-e113880-s009.zip › Fig1/1H/Project_E18.5 n2 b.tif]

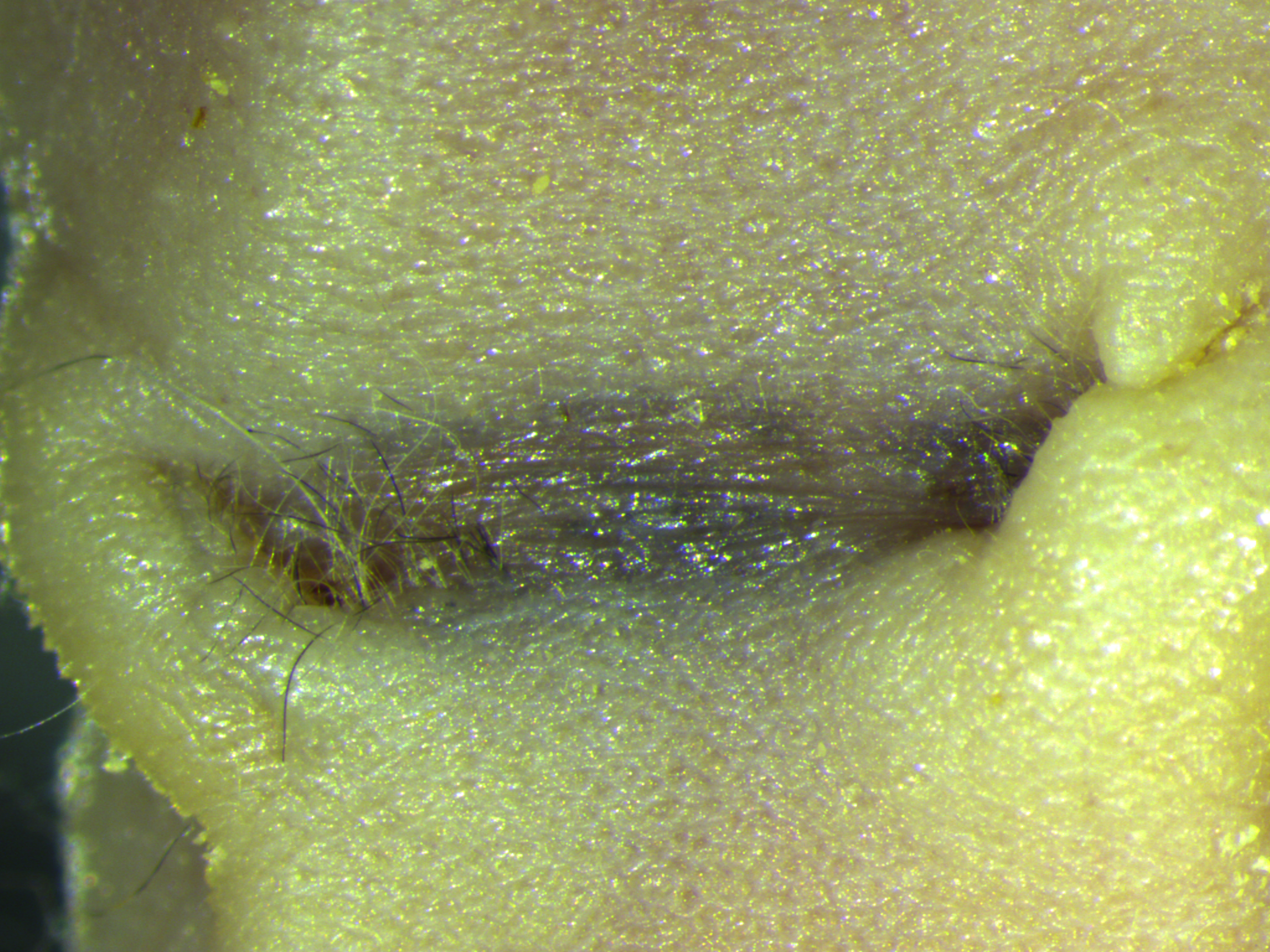

Supplement: Supplementary file 3 — Source Data for Figure 1 [file EMBJ-42-e113880-s009.zip › Fig1/1H/Project_P5 n2 b.tif]

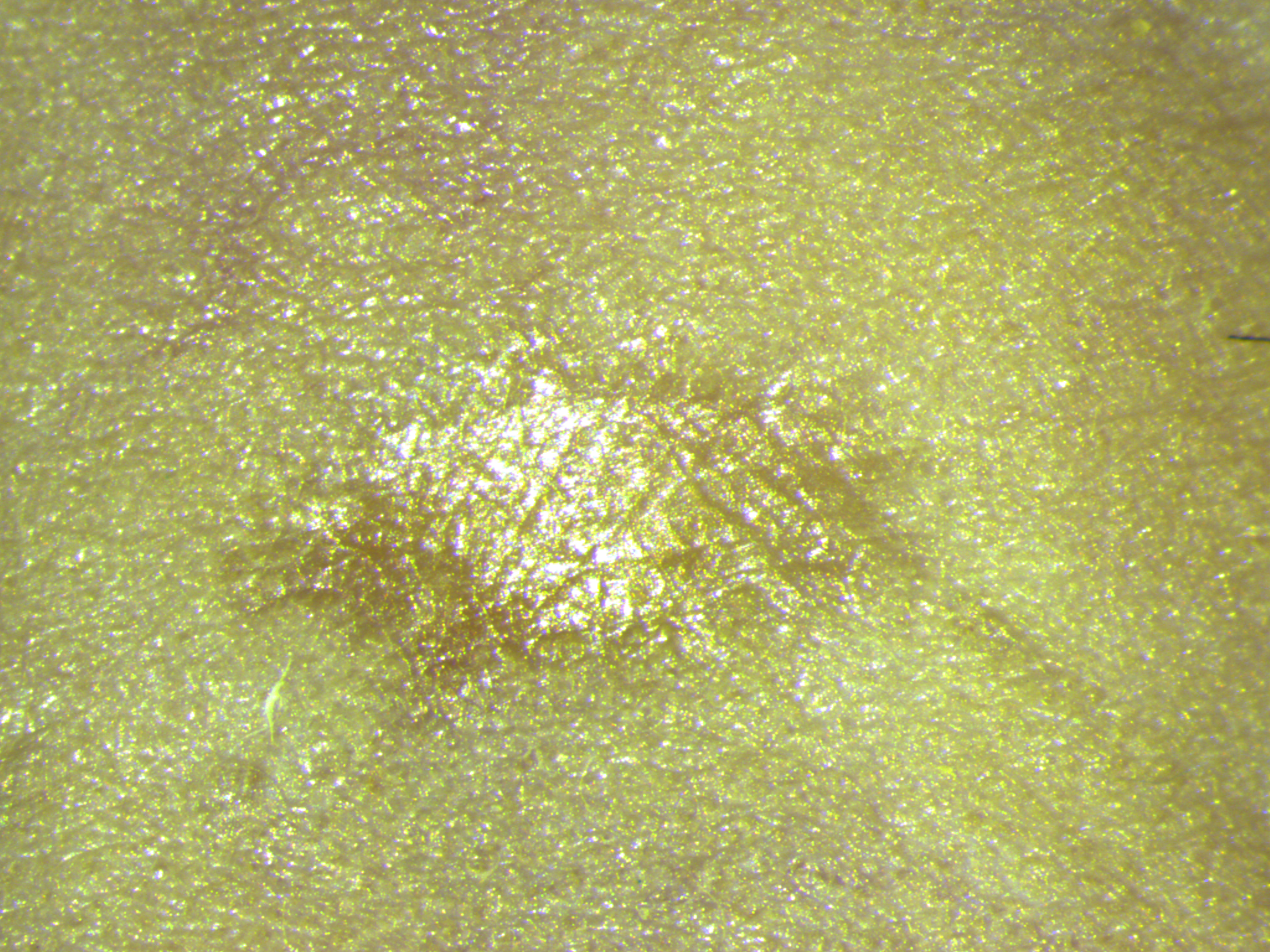

Supplement: Supplementary file 3 — Source Data for Figure 1 [file EMBJ-42-e113880-s009.zip › Fig1/1H/Project_E14.5 n3.tif]

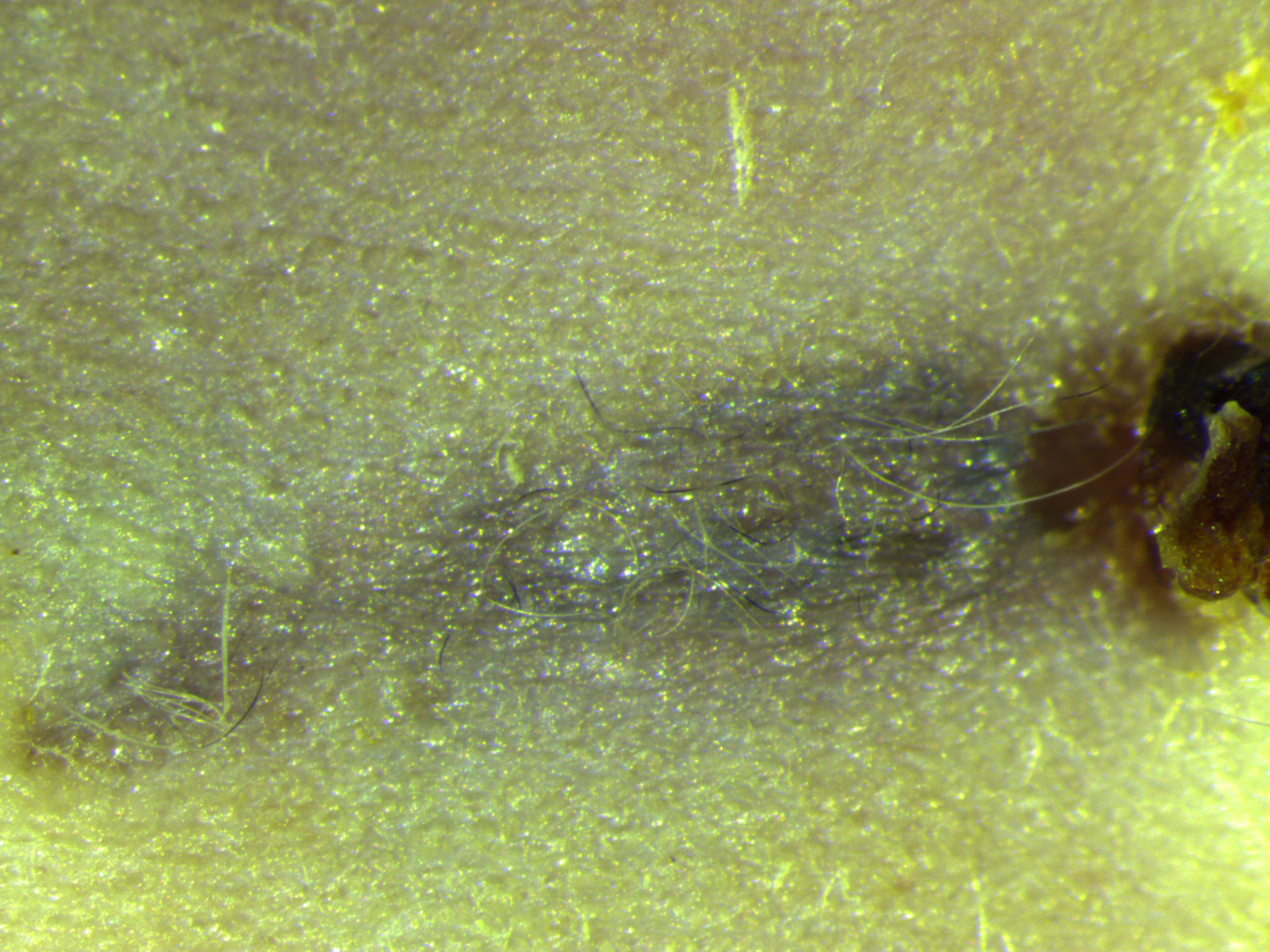

Supplement: Supplementary file 3 — Source Data for Figure 1 [file EMBJ-42-e113880-s009.zip › Fig1/1H/Project_P5 n4.tif]

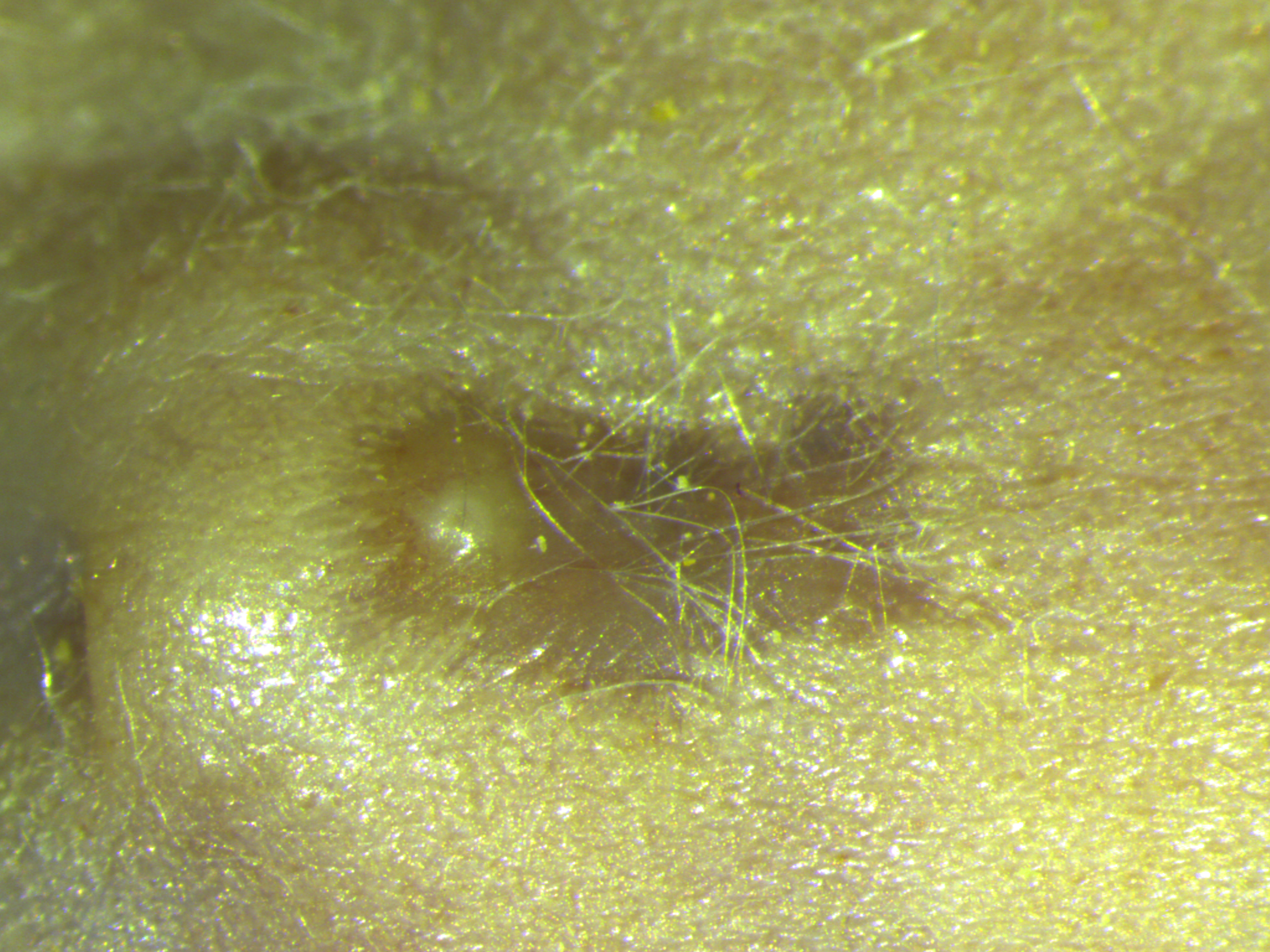

Supplement: Supplementary file 3 — Source Data for Figure 1 [file EMBJ-42-e113880-s009.zip › Fig1/1H/Project_E14.5 n2.tif]

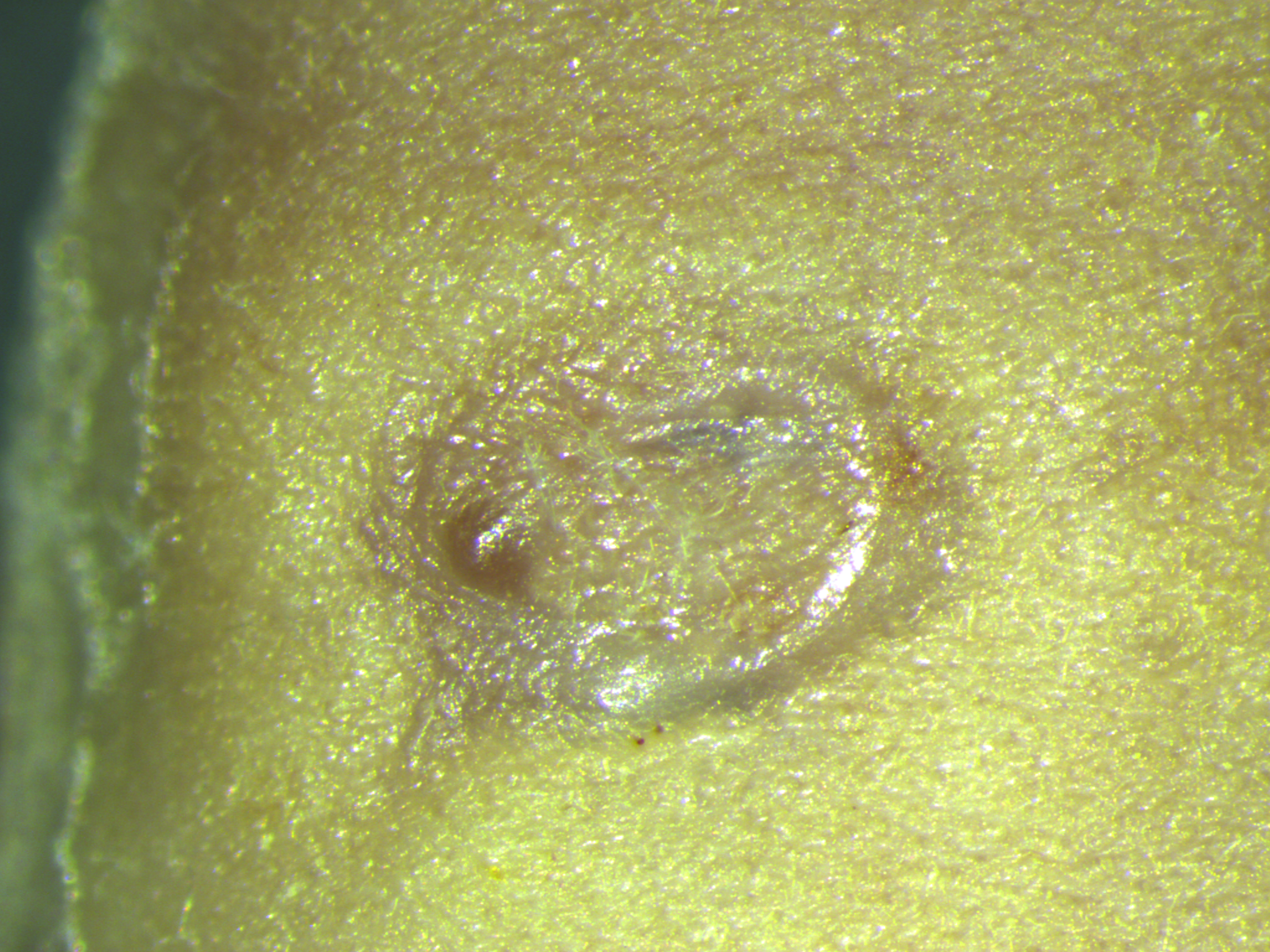

Supplement: Supplementary file 3 — Source Data for Figure 1 [file EMBJ-42-e113880-s009.zip › Fig1/1H/Project_E14.5 n1.tif]

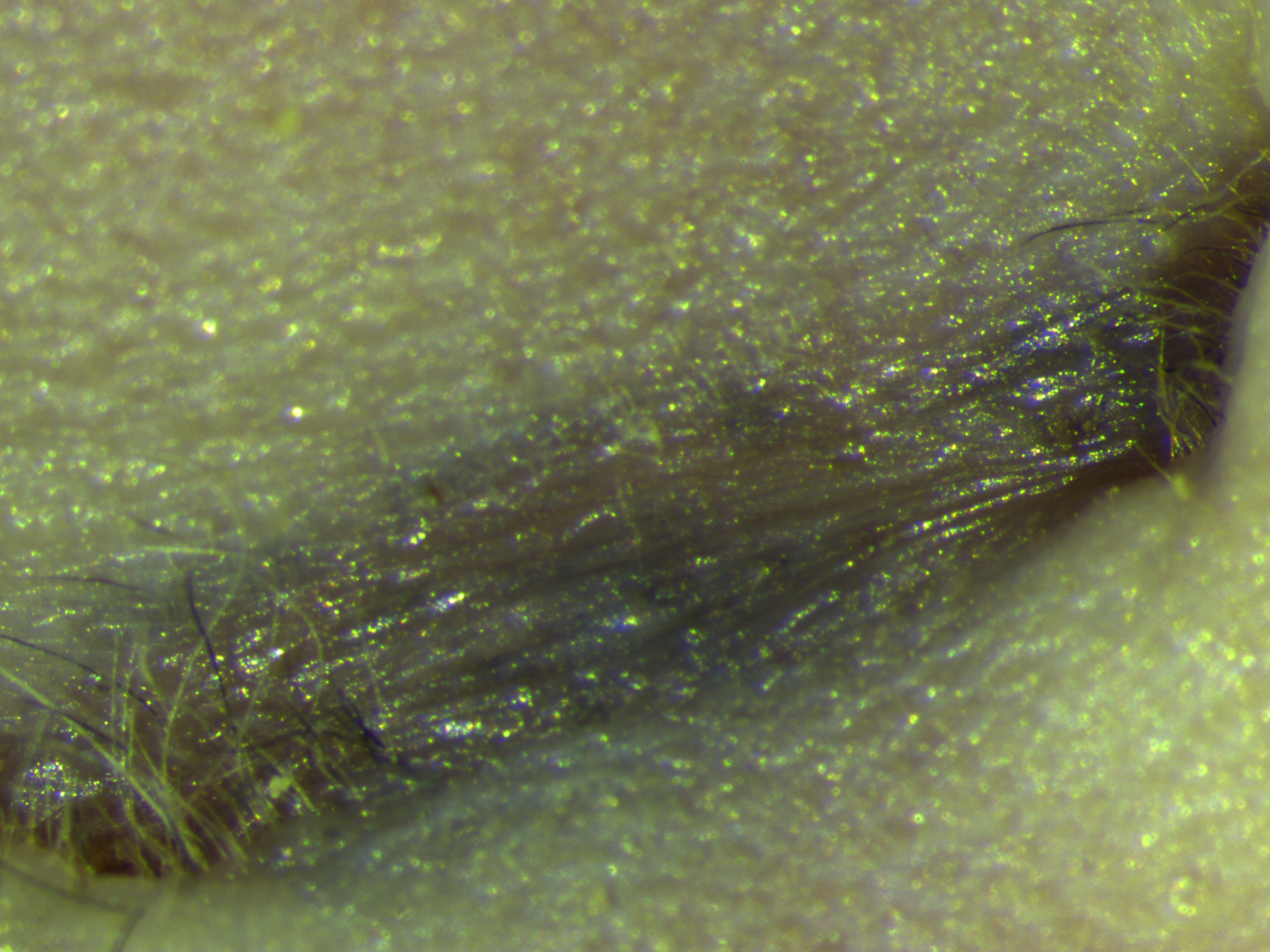

Supplement: Supplementary file 3 — Source Data for Figure 1 [file EMBJ-42-e113880-s009.zip › Fig1/1H/Project_P5 n2.tif]

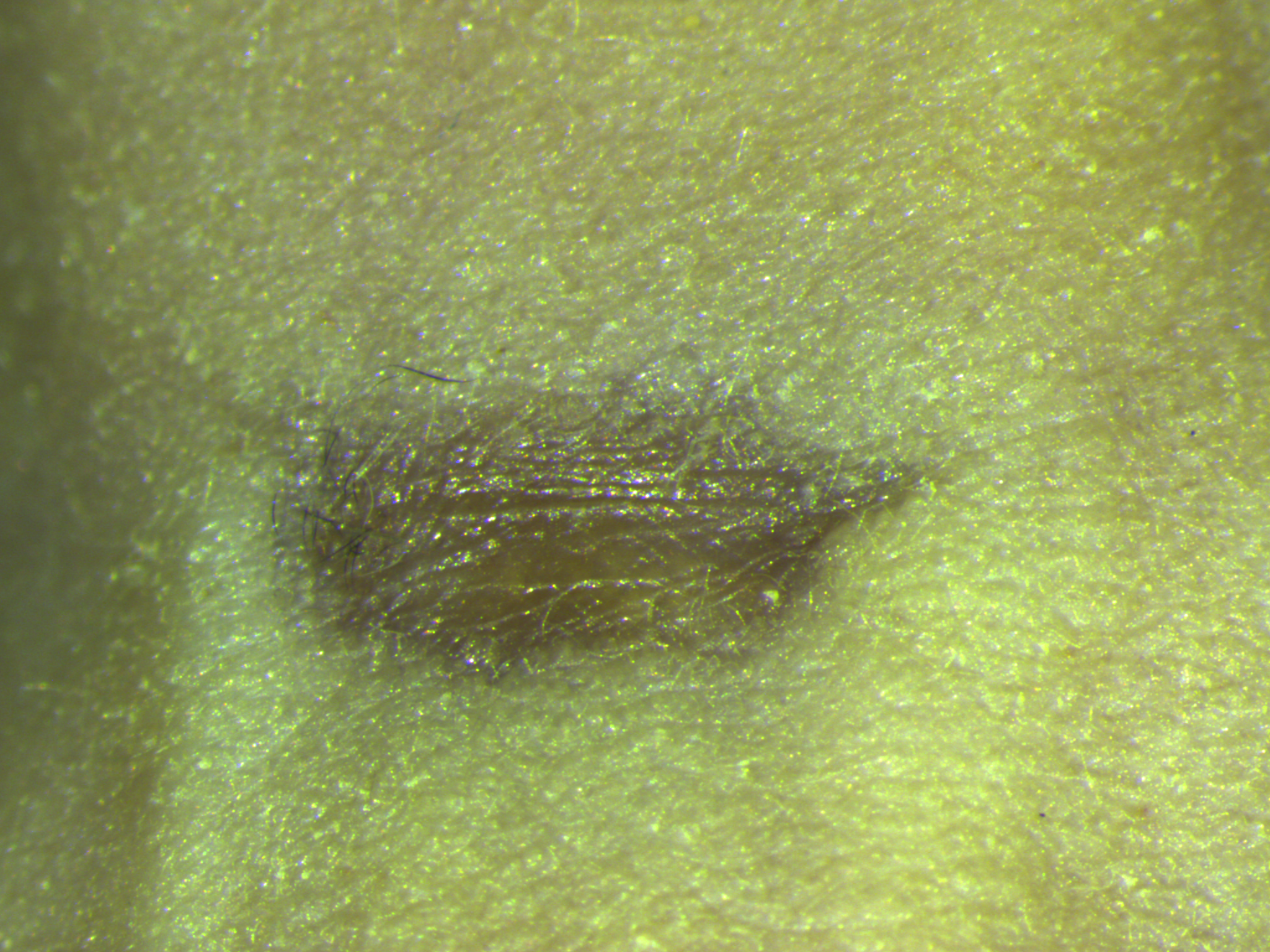

Supplement: Supplementary file 3 — Source Data for Figure 1 [file EMBJ-42-e113880-s009.zip › Fig1/1H/Project_P5 n3.tif]

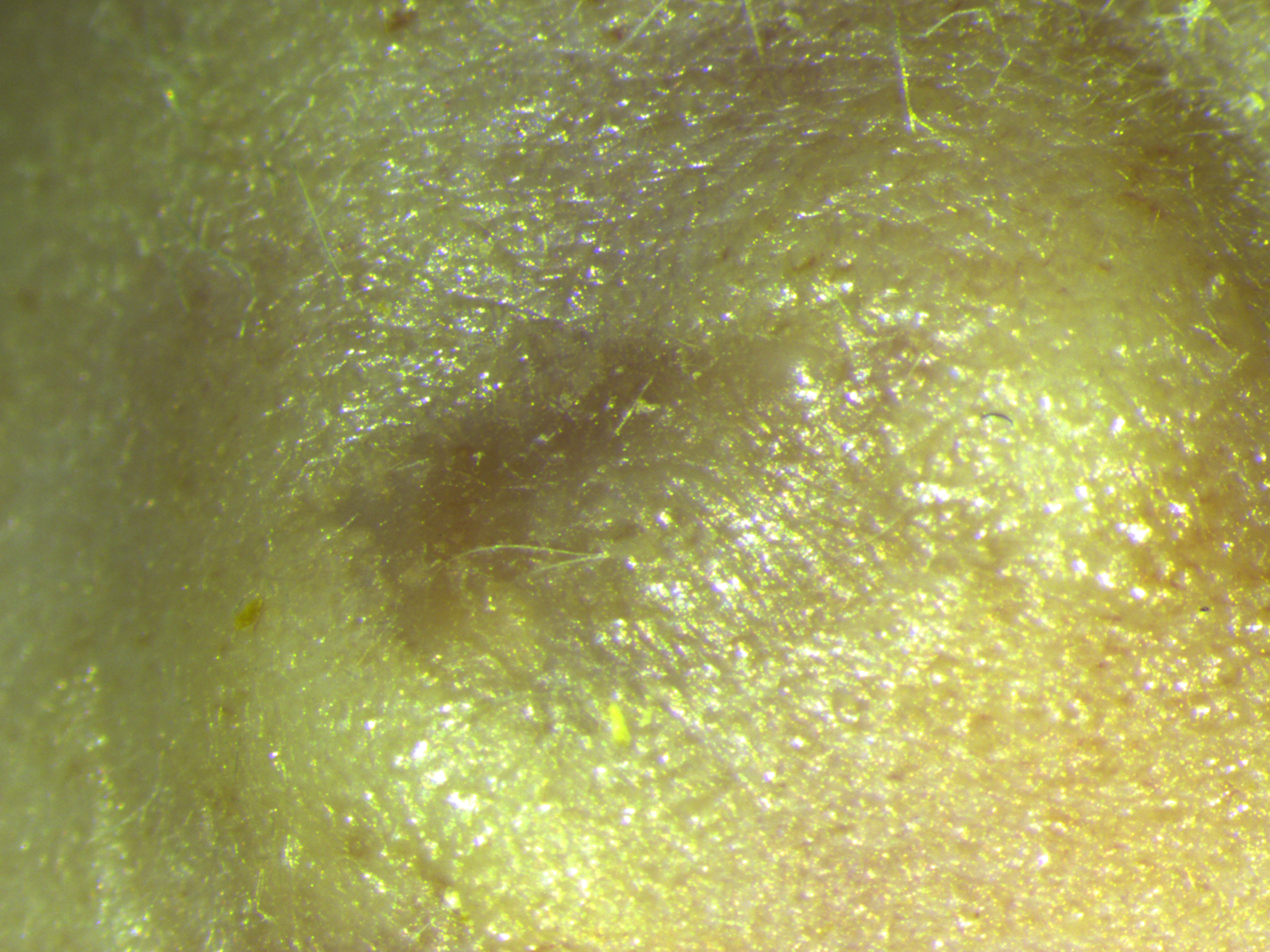

Supplement: Supplementary file 3 — Source Data for Figure 1 [file EMBJ-42-e113880-s009.zip › Fig1/1H/Project_E14.5 n4.tif]

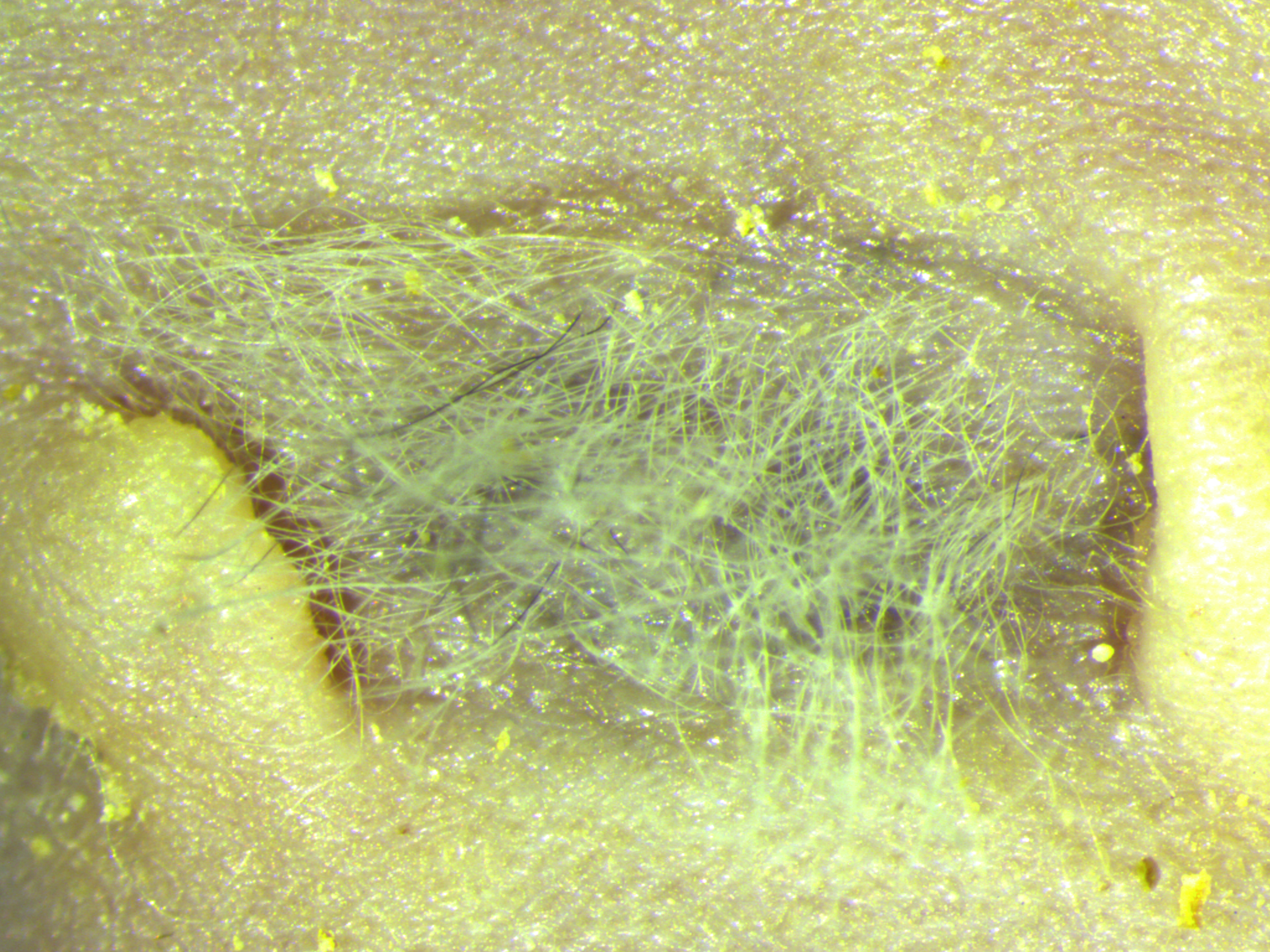

Supplement: Supplementary file 3 — Source Data for Figure 1 [file EMBJ-42-e113880-s009.zip › Fig1/1H/Project_E 18.5 n1 b.tif]

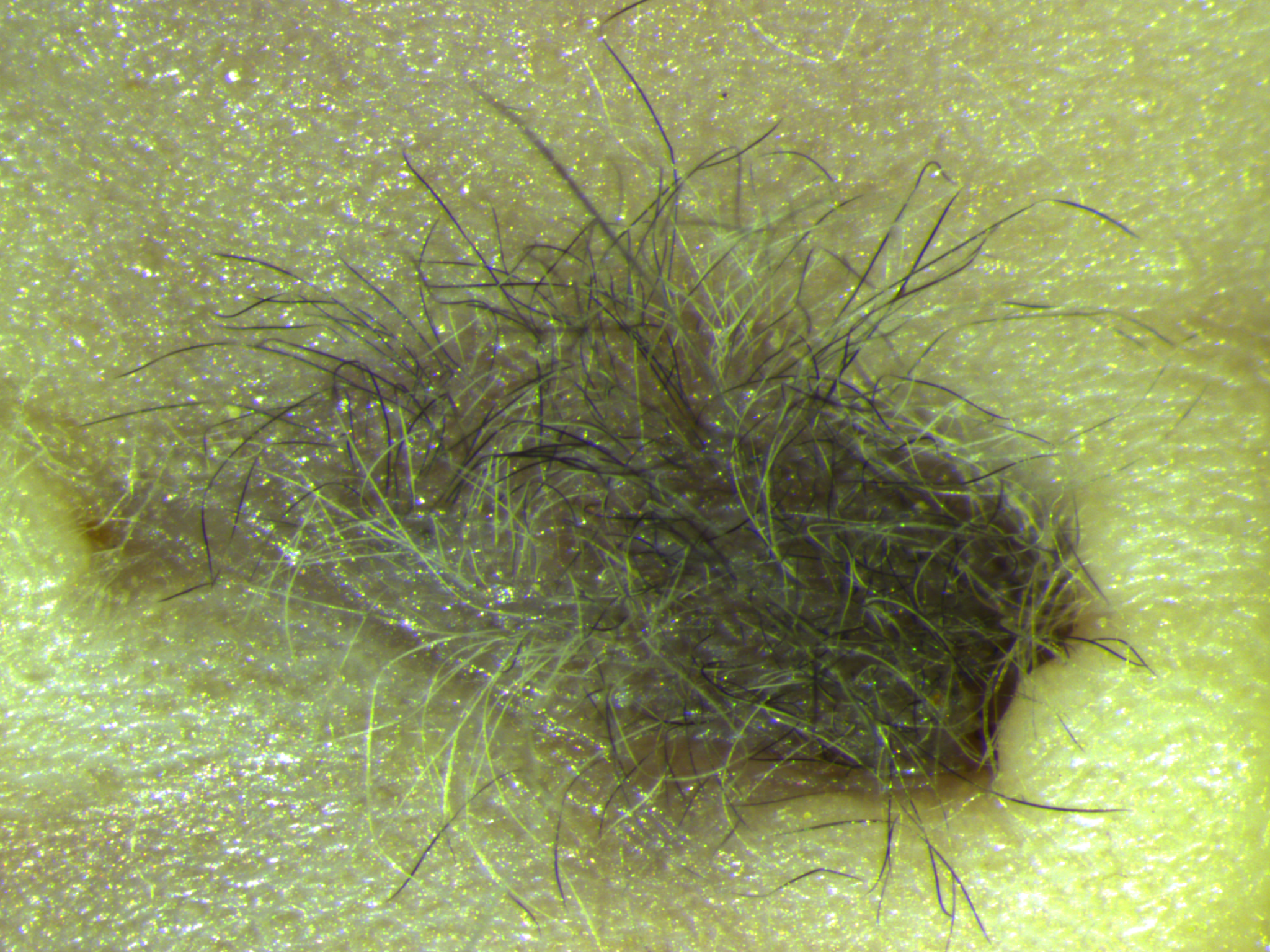

Supplement: Supplementary file 3 — Source Data for Figure 1 [file EMBJ-42-e113880-s009.zip › Fig1/1H/Project_P5 n1.tif]

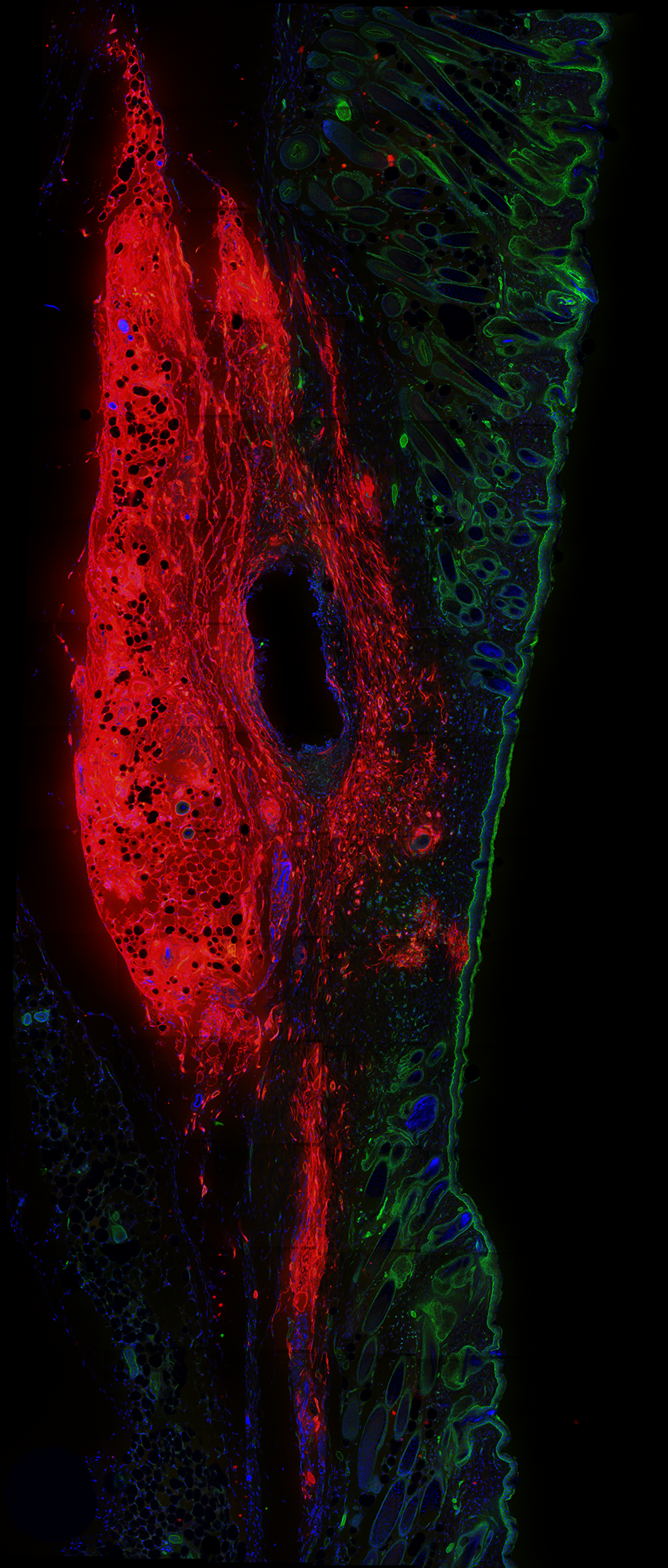

Supplement: Supplementary file 3 — Source Data for Figure 1 [file EMBJ-42-e113880-s009.zip › Fig1/1H/Fig1H_E14.tif]

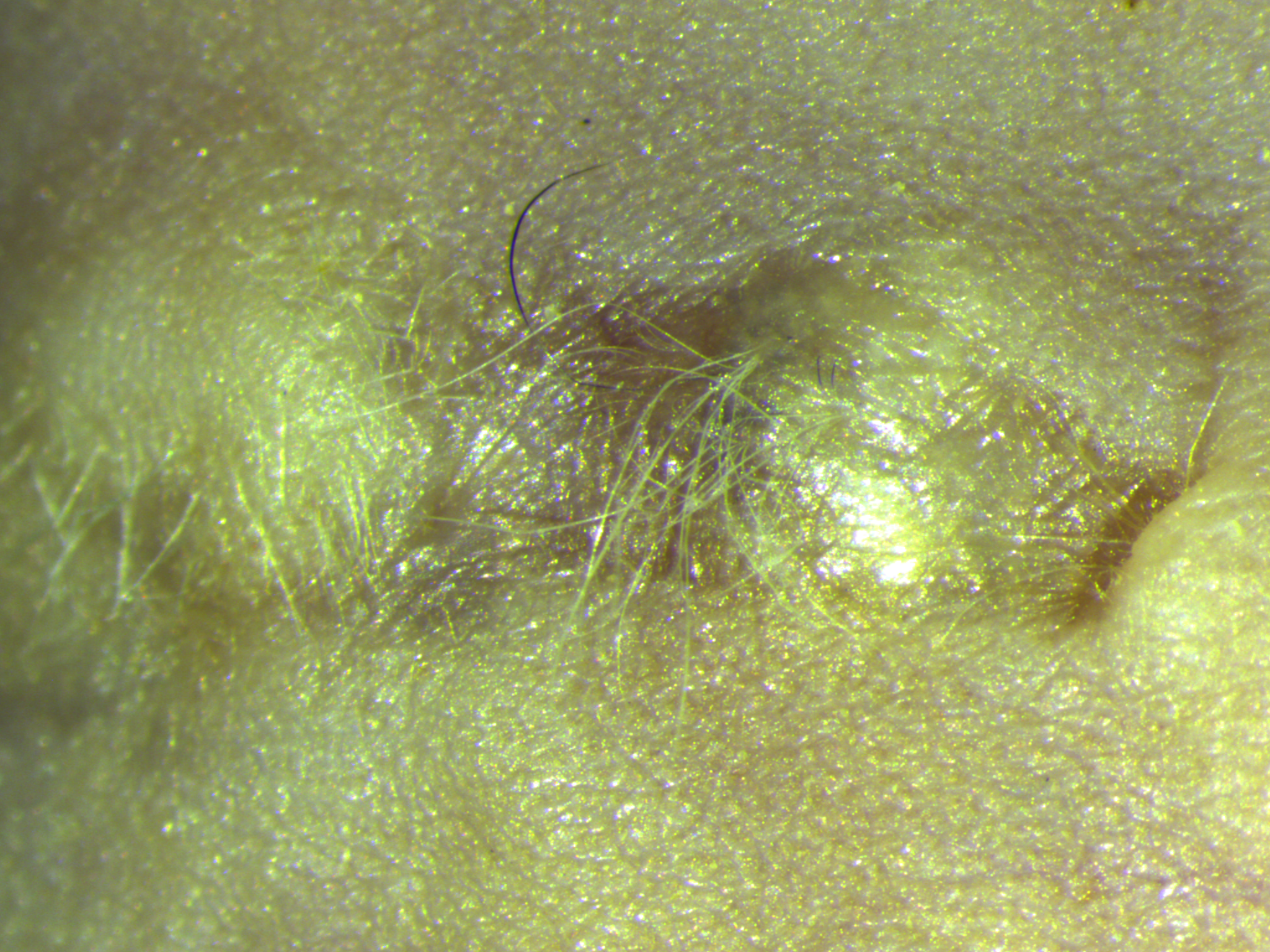

Supplement: Supplementary file 3 — Source Data for Figure 1 [file EMBJ-42-e113880-s009.zip › Fig1/1H/Project_E18.5 n4.tif]

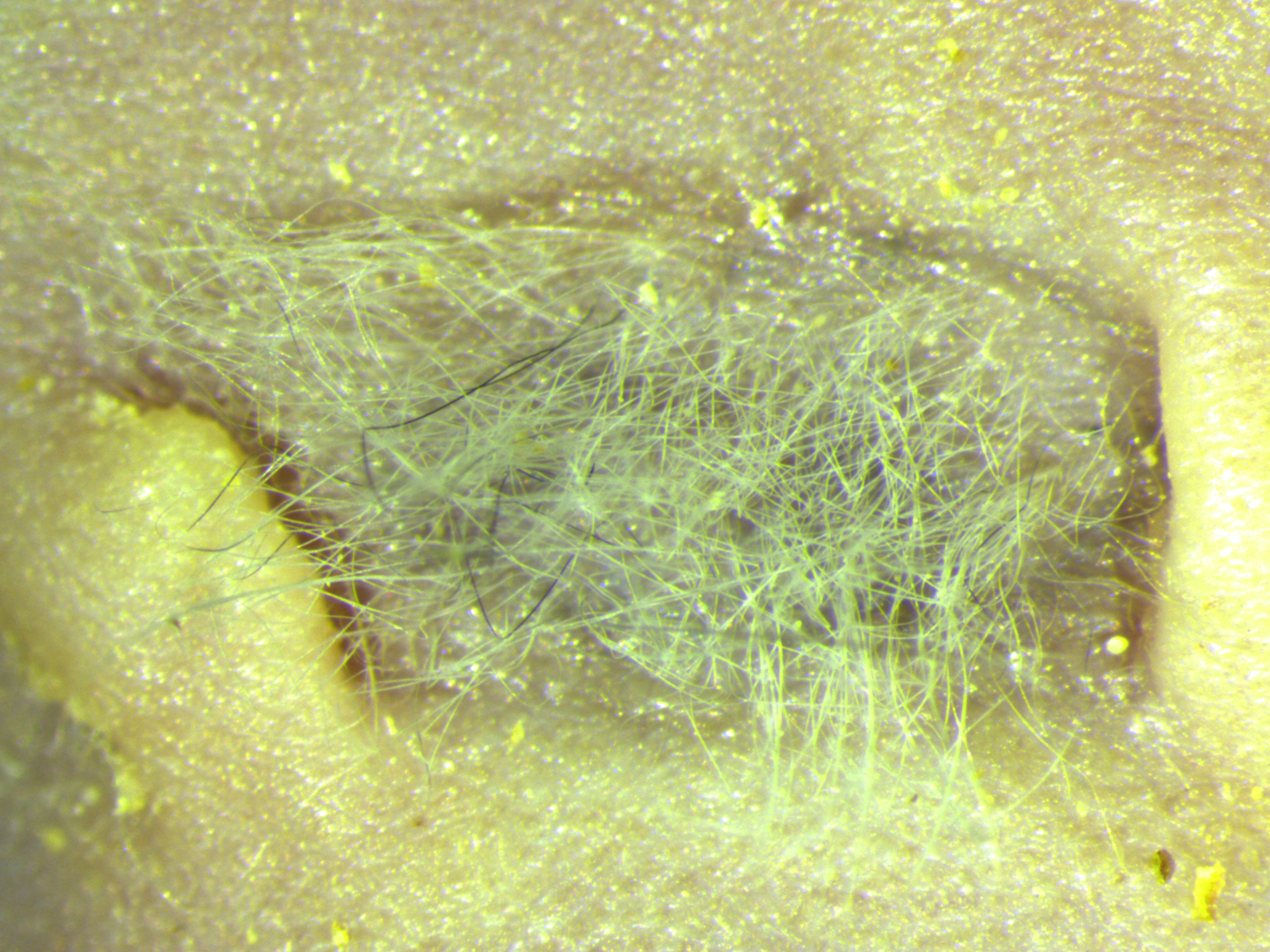

Supplement: Supplementary file 3 — Source Data for Figure 1 [file EMBJ-42-e113880-s009.zip › Fig1/1H/Project_E18.5 n1.tif]

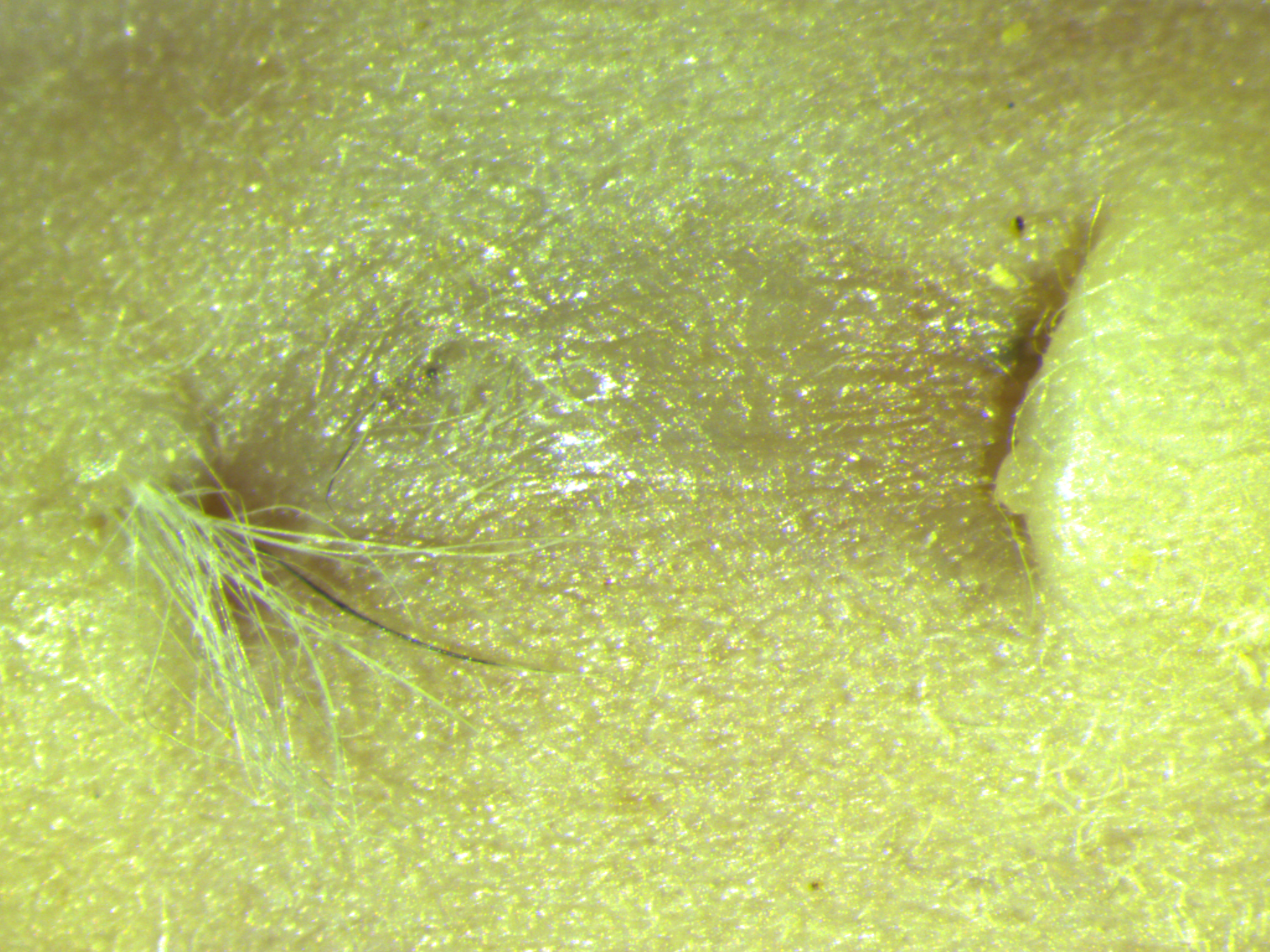

Supplement: Supplementary file 3 — Source Data for Figure 1 [file EMBJ-42-e113880-s009.zip › Fig1/1H/Project_E18.5 n3.tif]

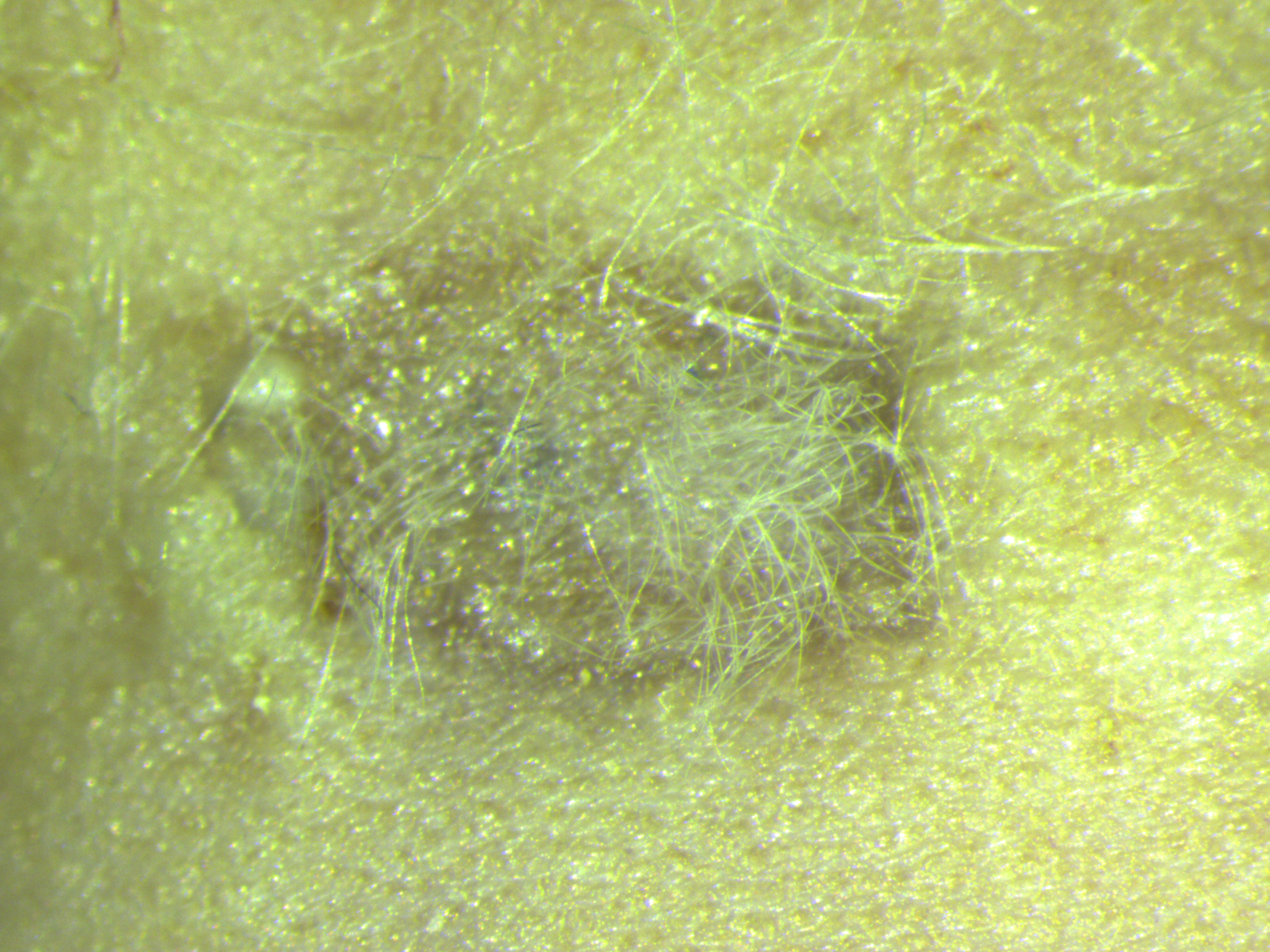

Supplement: Supplementary file 3 — Source Data for Figure 1 [file EMBJ-42-e113880-s009.zip › Fig1/1H/Project_E18.5 n2.tif]

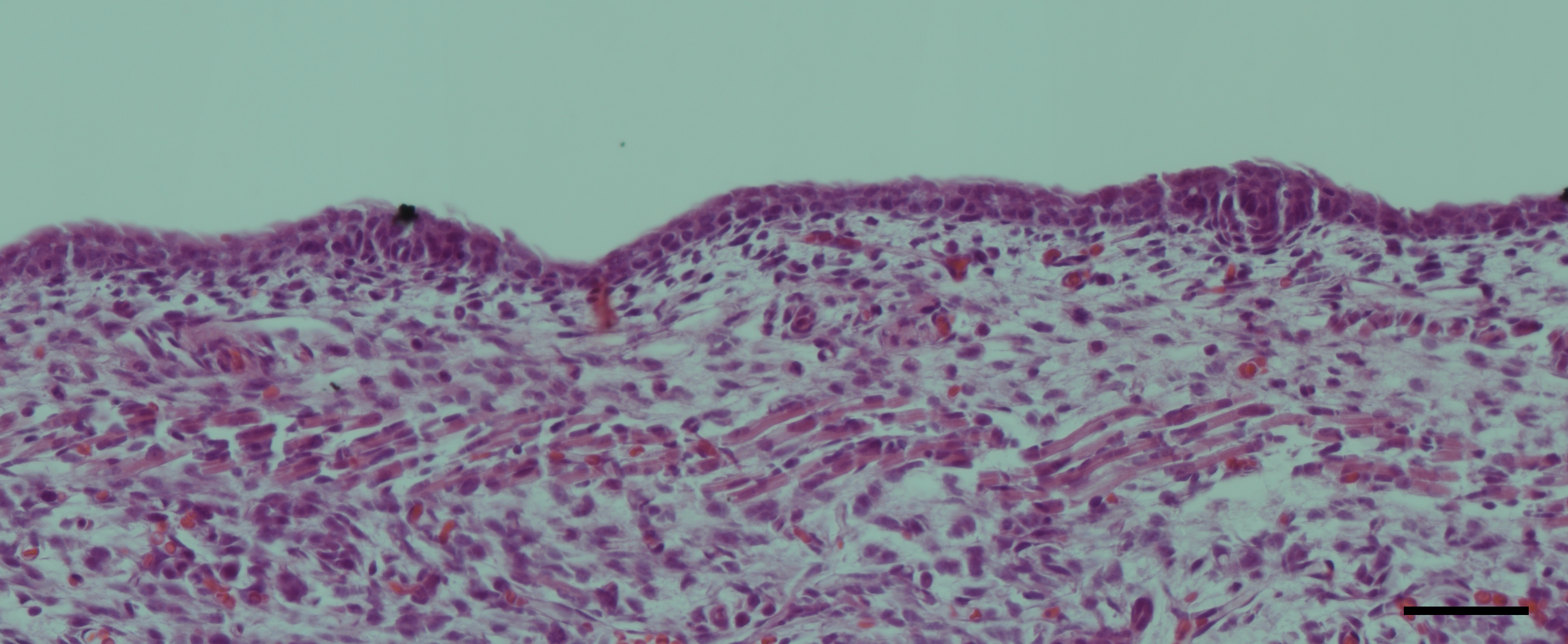

Supplement: Supplementary file 3 — Source Data for Figure 1 [file EMBJ-42-e113880-s009.zip › Fig1/1A/Fig1A_E14.tif]

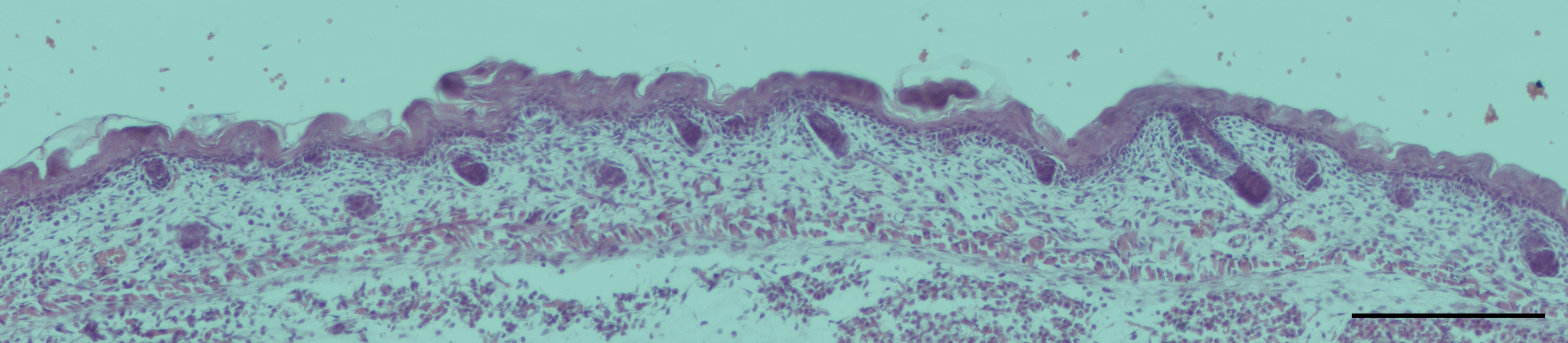

Supplement: Supplementary file 3 — Source Data for Figure 1 [file EMBJ-42-e113880-s009.zip › Fig1/1B/Fig1B_E17.tif]

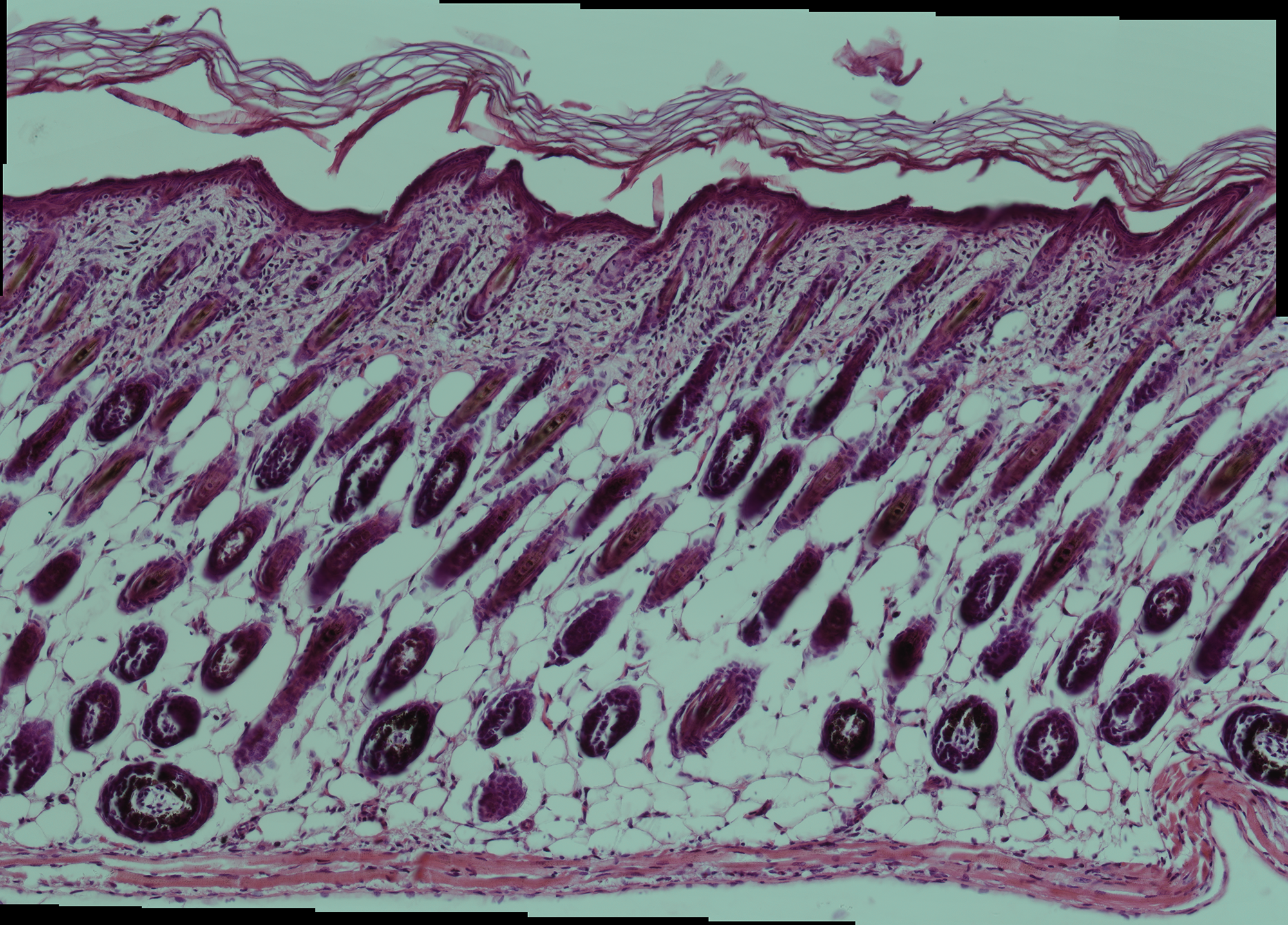

Supplement: Supplementary file 3 — Source Data for Figure 1 [file EMBJ-42-e113880-s009.zip › Fig1/1C/Fig1C_P5.tif]

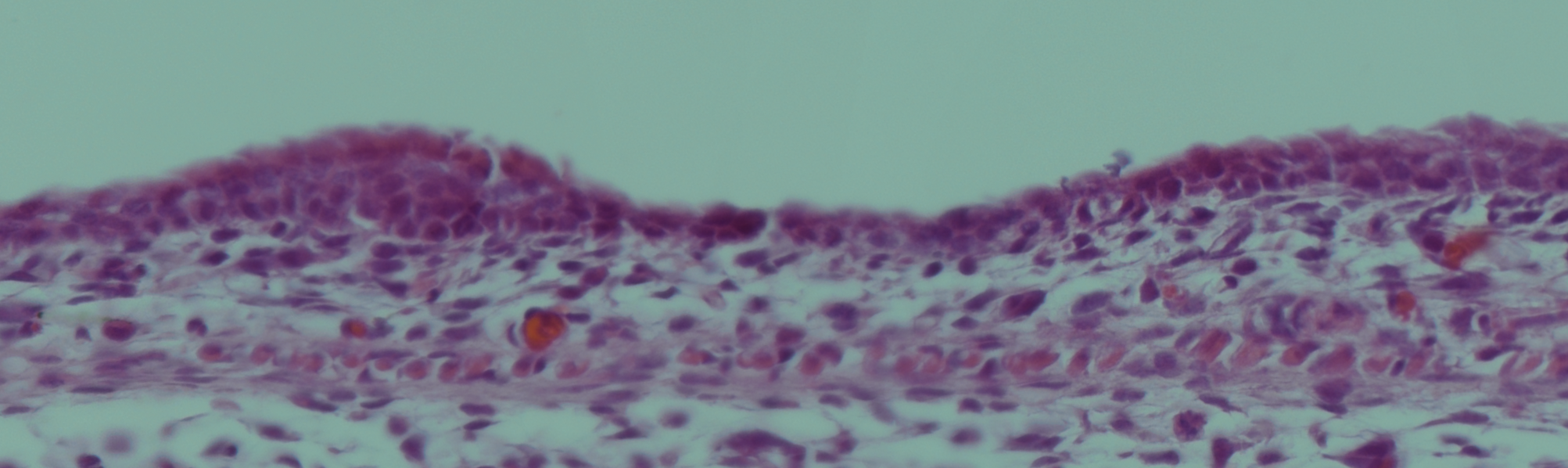

Supplement: Supplementary file 6 — Source Data for Figure 4 [file EMBJ-42-e113880-s003.zip › Fig4/4B/WT.tif]

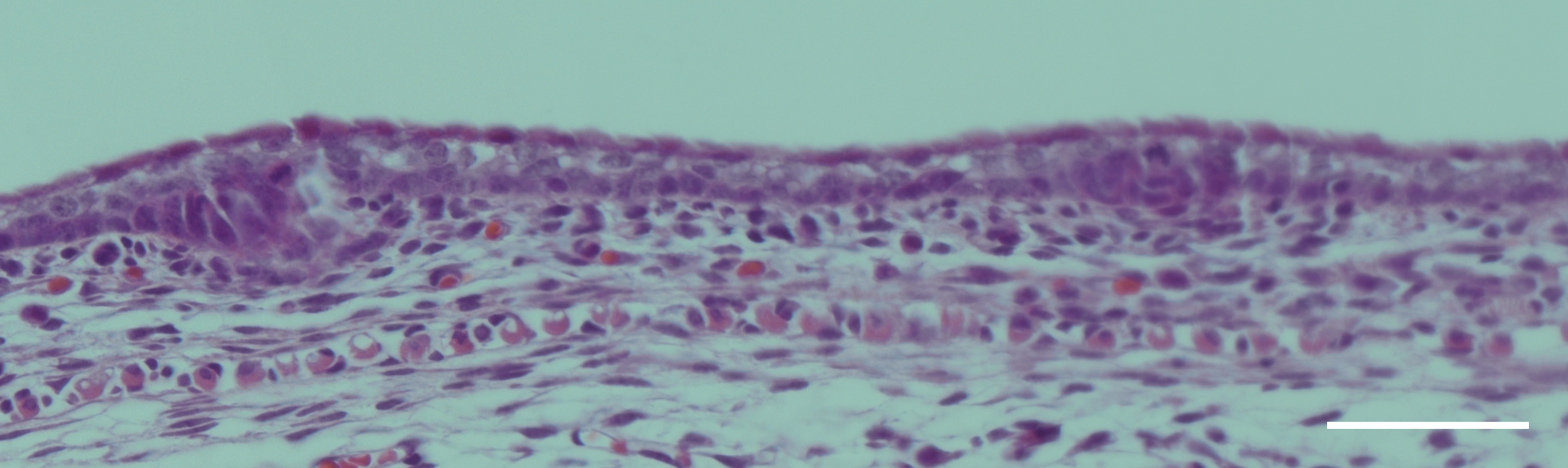

Supplement: Supplementary file 6 — Source Data for Figure 4 [file EMBJ-42-e113880-s003.zip › Fig4/4B/KO.tif]

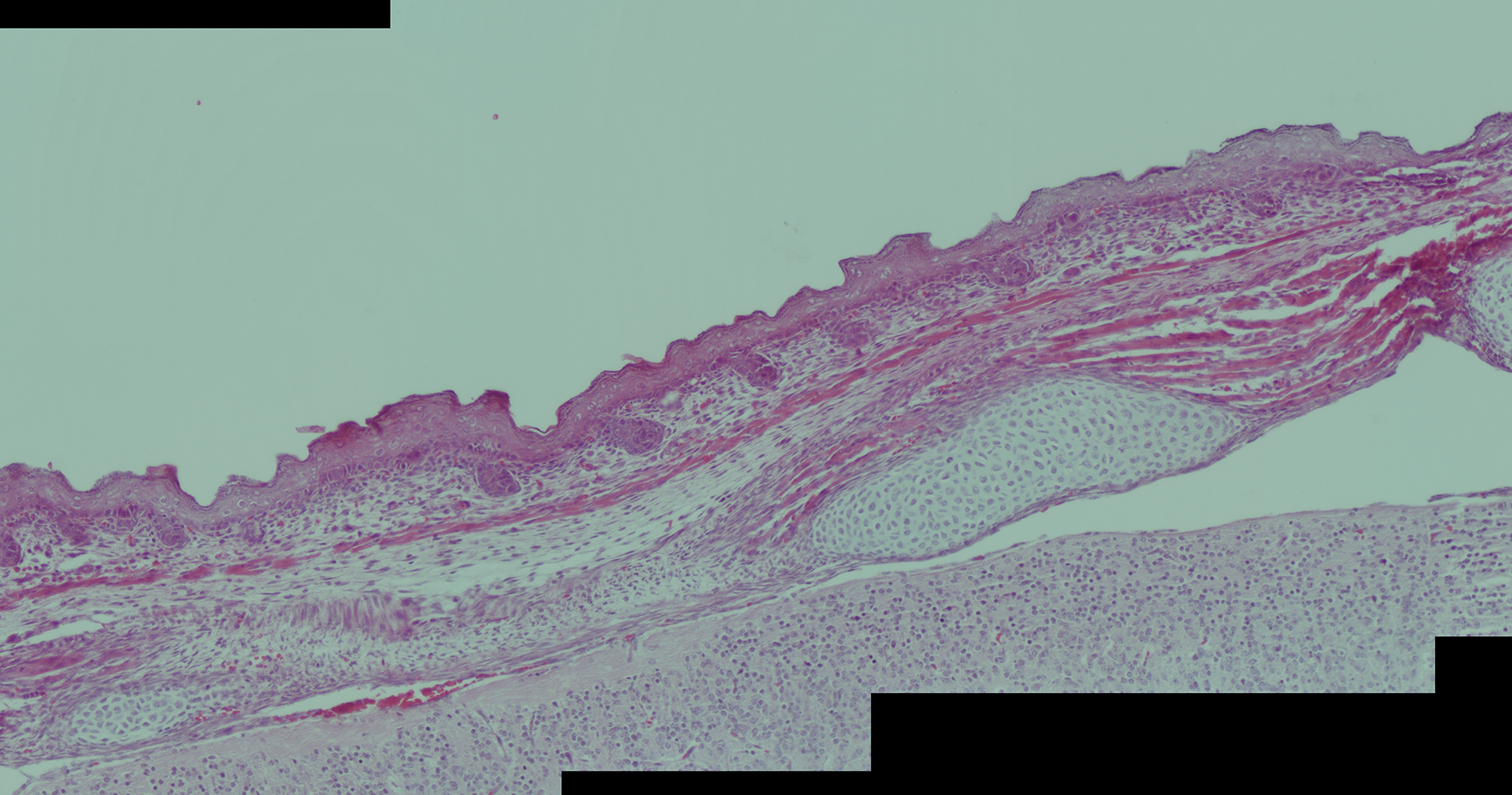

Supplement: Supplementary file 6 — Source Data for Figure 4 [file EMBJ-42-e113880-s003.zip › Fig4/4C/Fig4C_KO.tif]

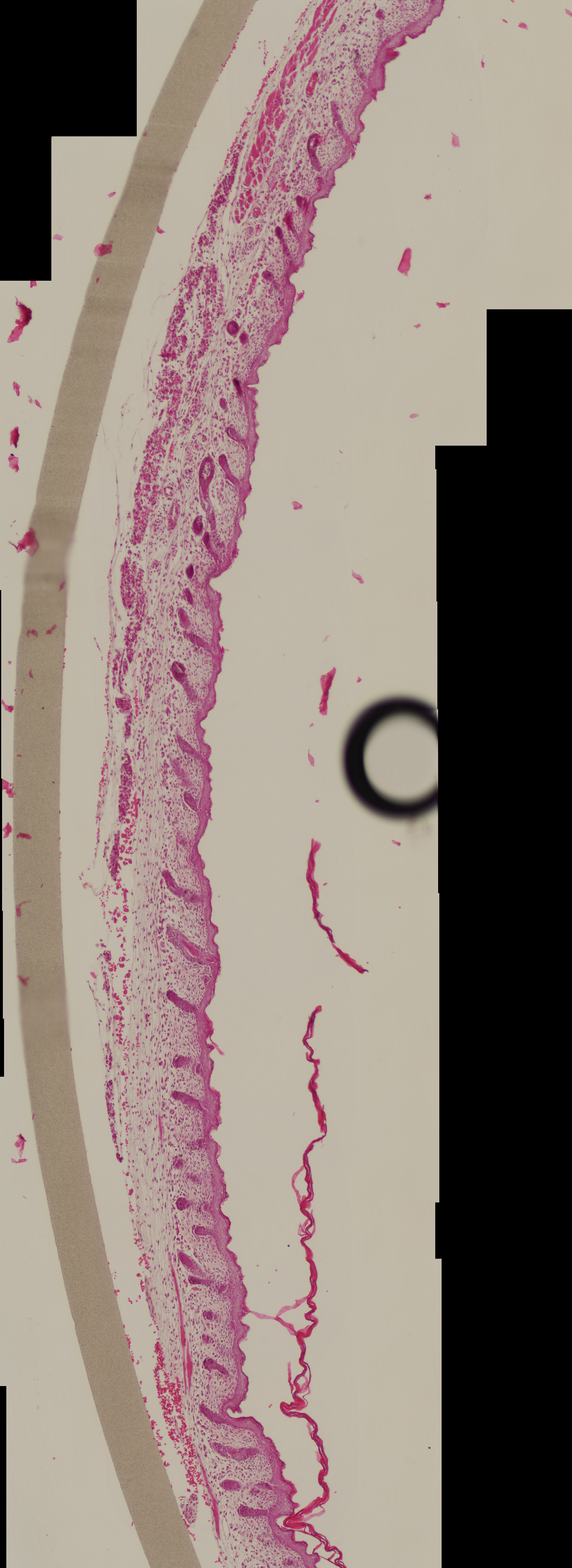

Supplement: Supplementary file 6 — Source Data for Figure 4 [file EMBJ-42-e113880-s003.zip › Fig4/4C/Fig4C_WT.tif]

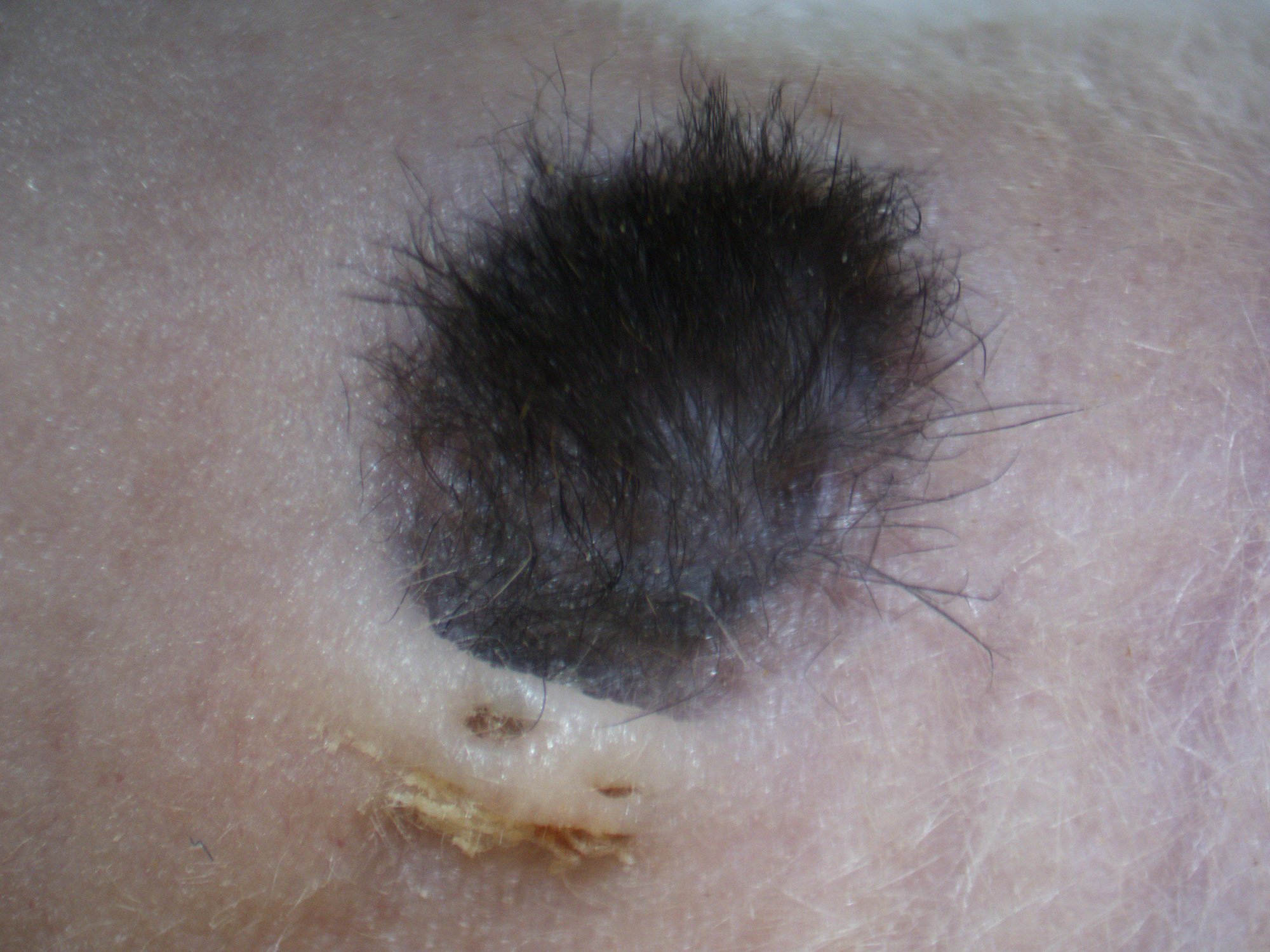

Supplement: Supplementary file 8 — Source Data for Figure 6 [file EMBJ-42-e113880-s006.zip › Fig6/6D/WT2.jpg]

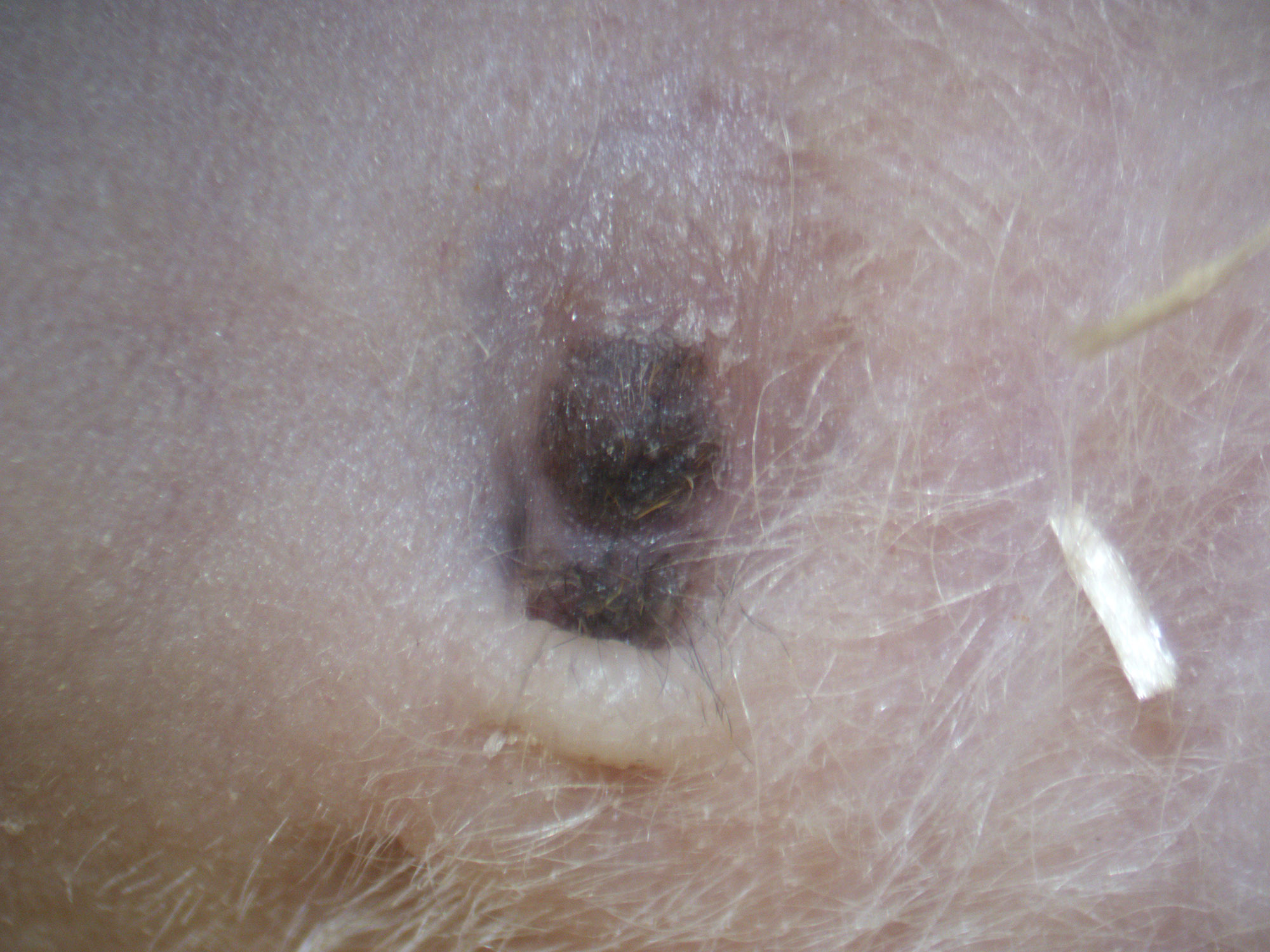

Supplement: Supplementary file 8 — Source Data for Figure 6 [file EMBJ-42-e113880-s006.zip › Fig6/6D/WT3.jpg]

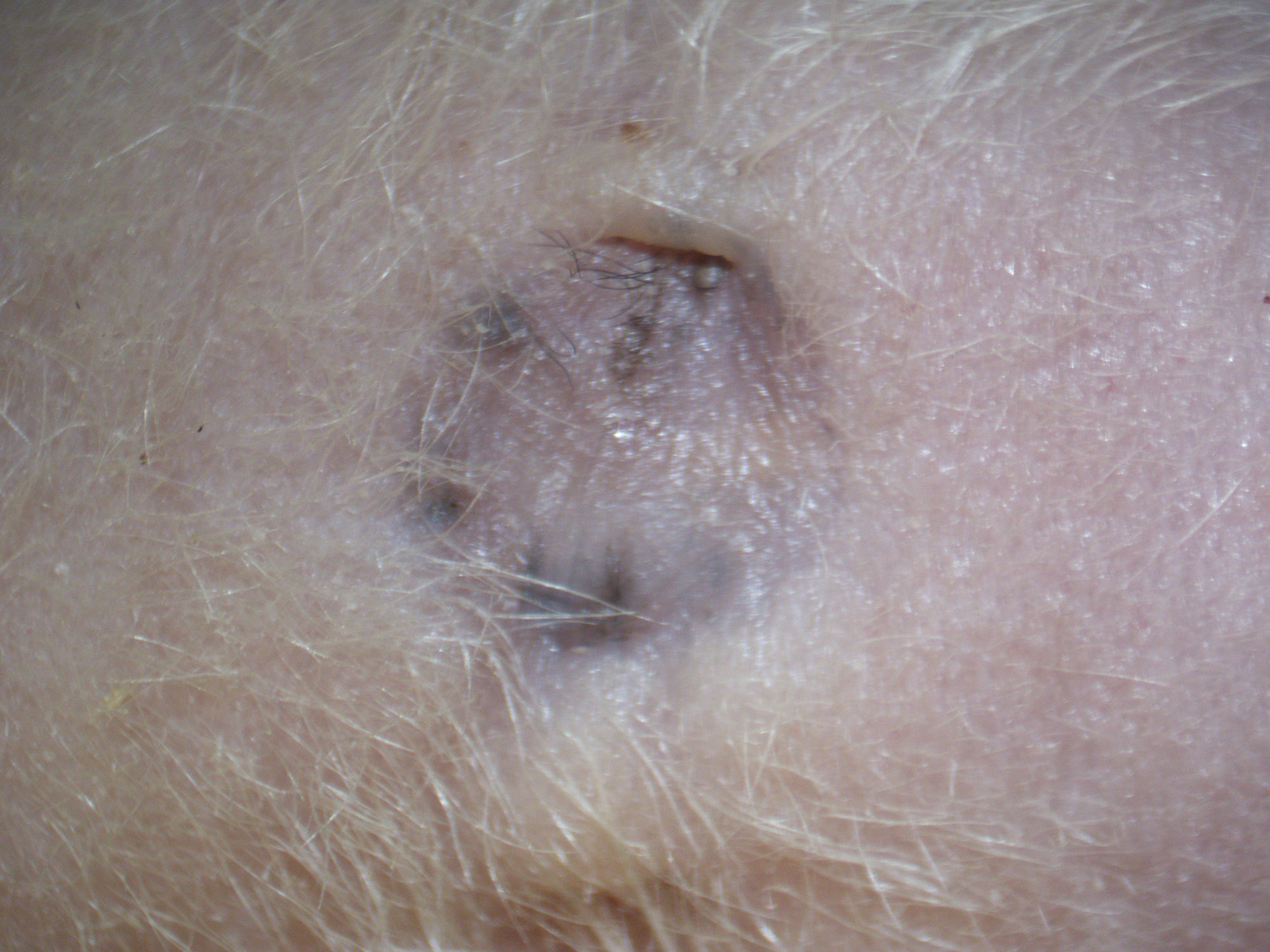

Supplement: Supplementary file 8 — Source Data for Figure 6 [file EMBJ-42-e113880-s006.zip › Fig6/6D/WT1.jpg]

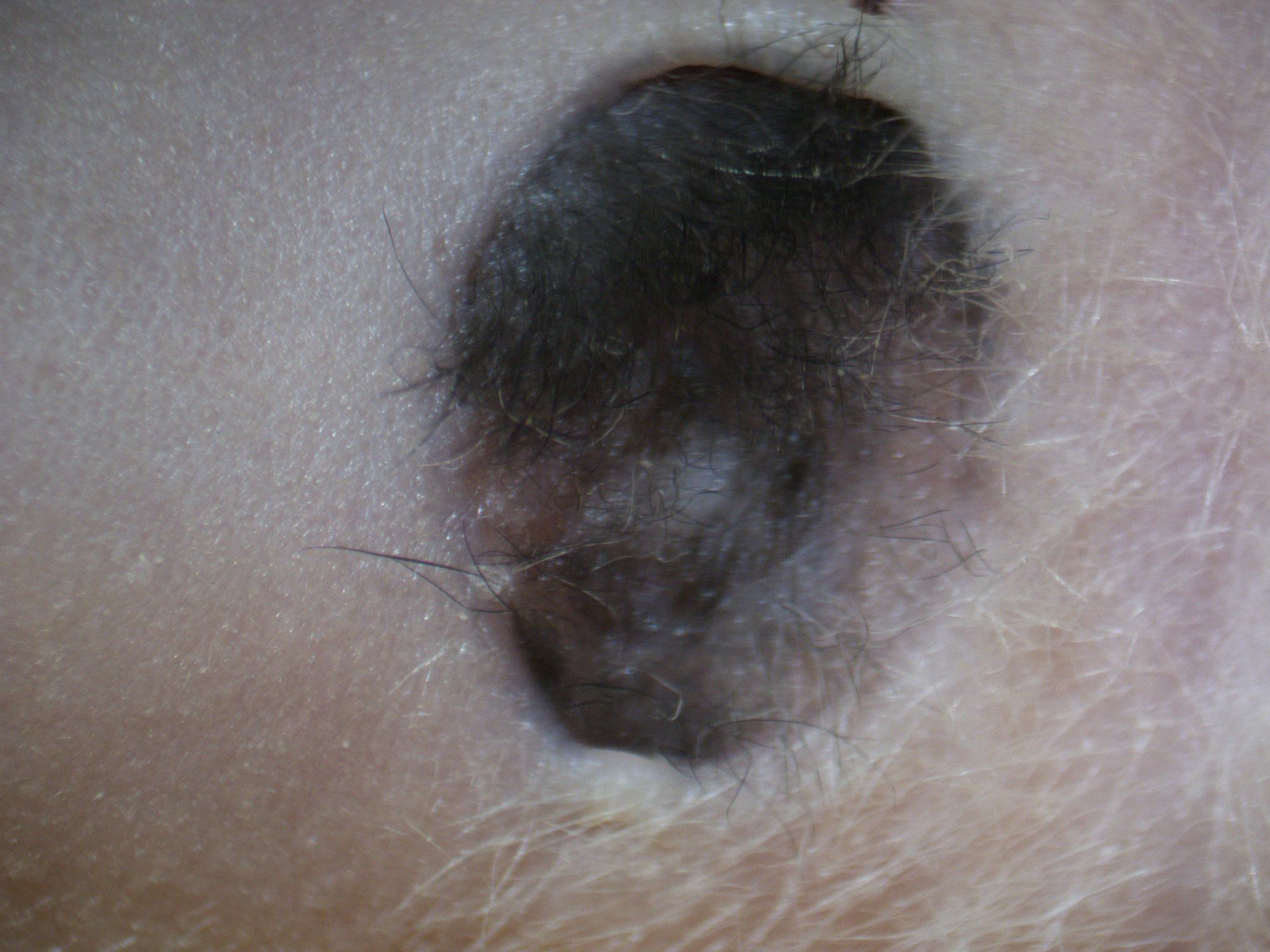

Supplement: Supplementary file 8 — Source Data for Figure 6 [file EMBJ-42-e113880-s006.zip › Fig6/6D/WT4.jpg]

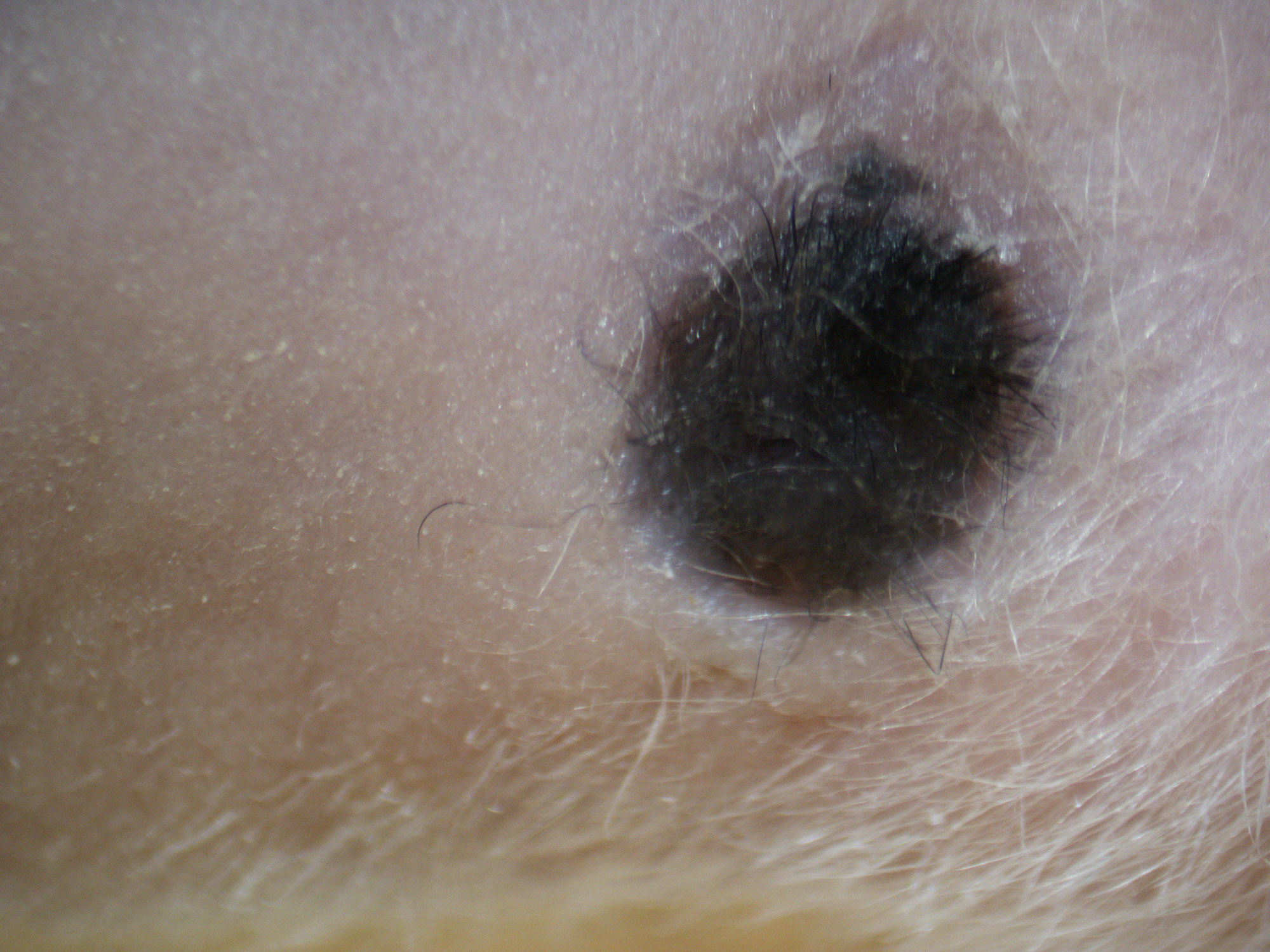

Supplement: Supplementary file 8 — Source Data for Figure 6 [file EMBJ-42-e113880-s006.zip › Fig6/6D/WT5.jpg]

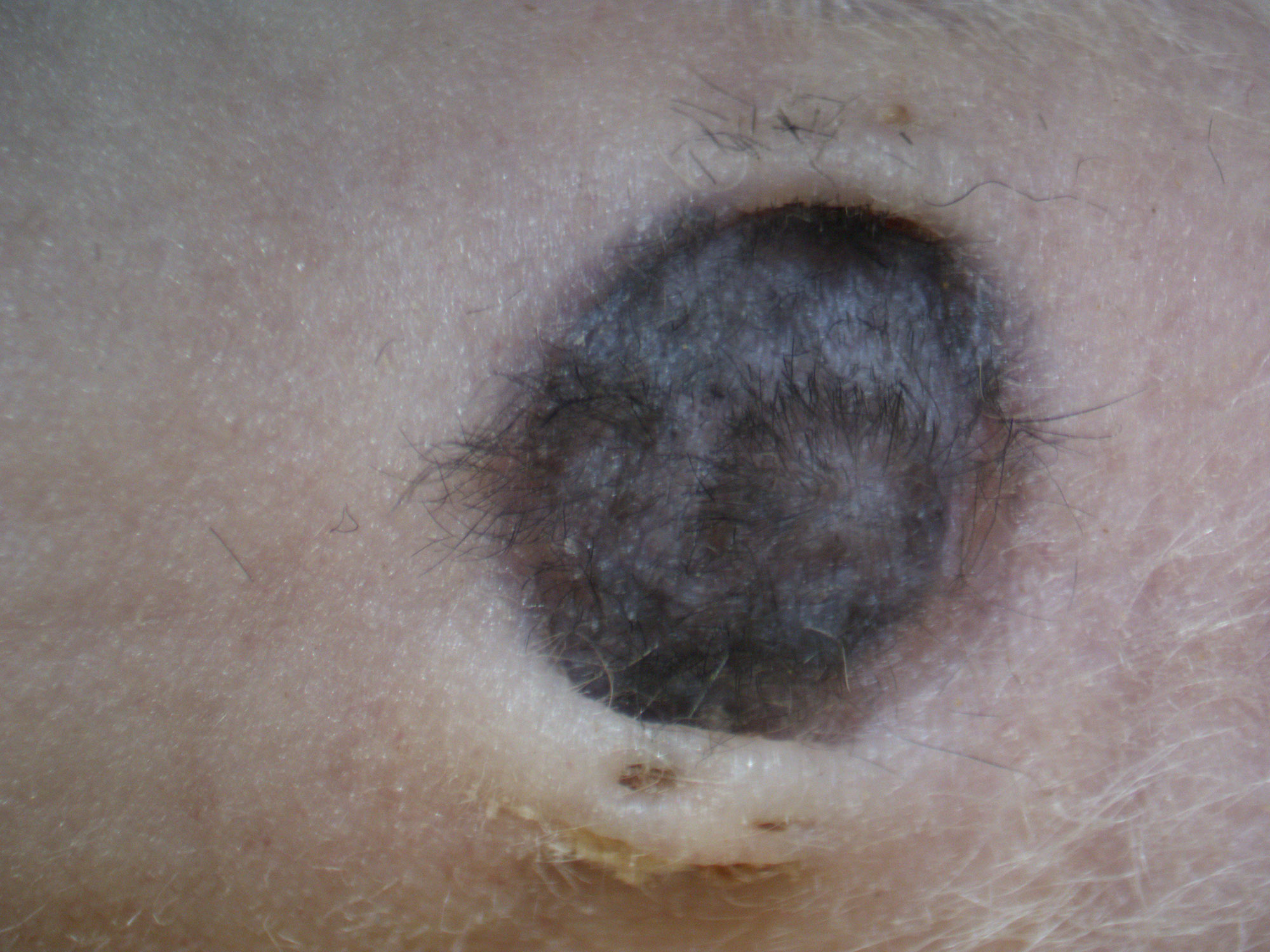

Supplement: Supplementary file 8 — Source Data for Figure 6 [file EMBJ-42-e113880-s006.zip › Fig6/6D/WT2_1.jpg]

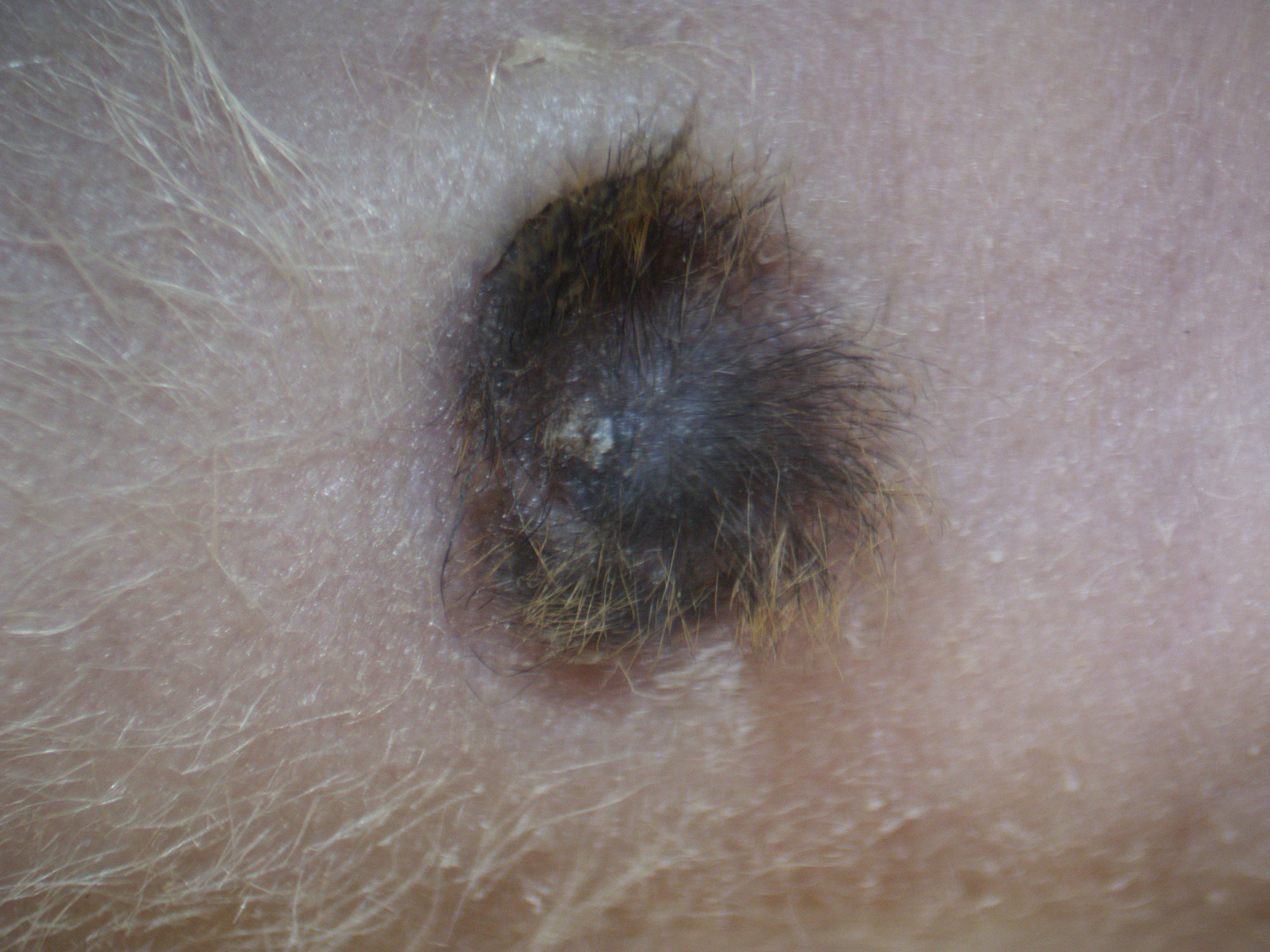

Supplement: Supplementary file 8 — Source Data for Figure 6 [file EMBJ-42-e113880-s006.zip › Fig6/6D/KO4.jpg]

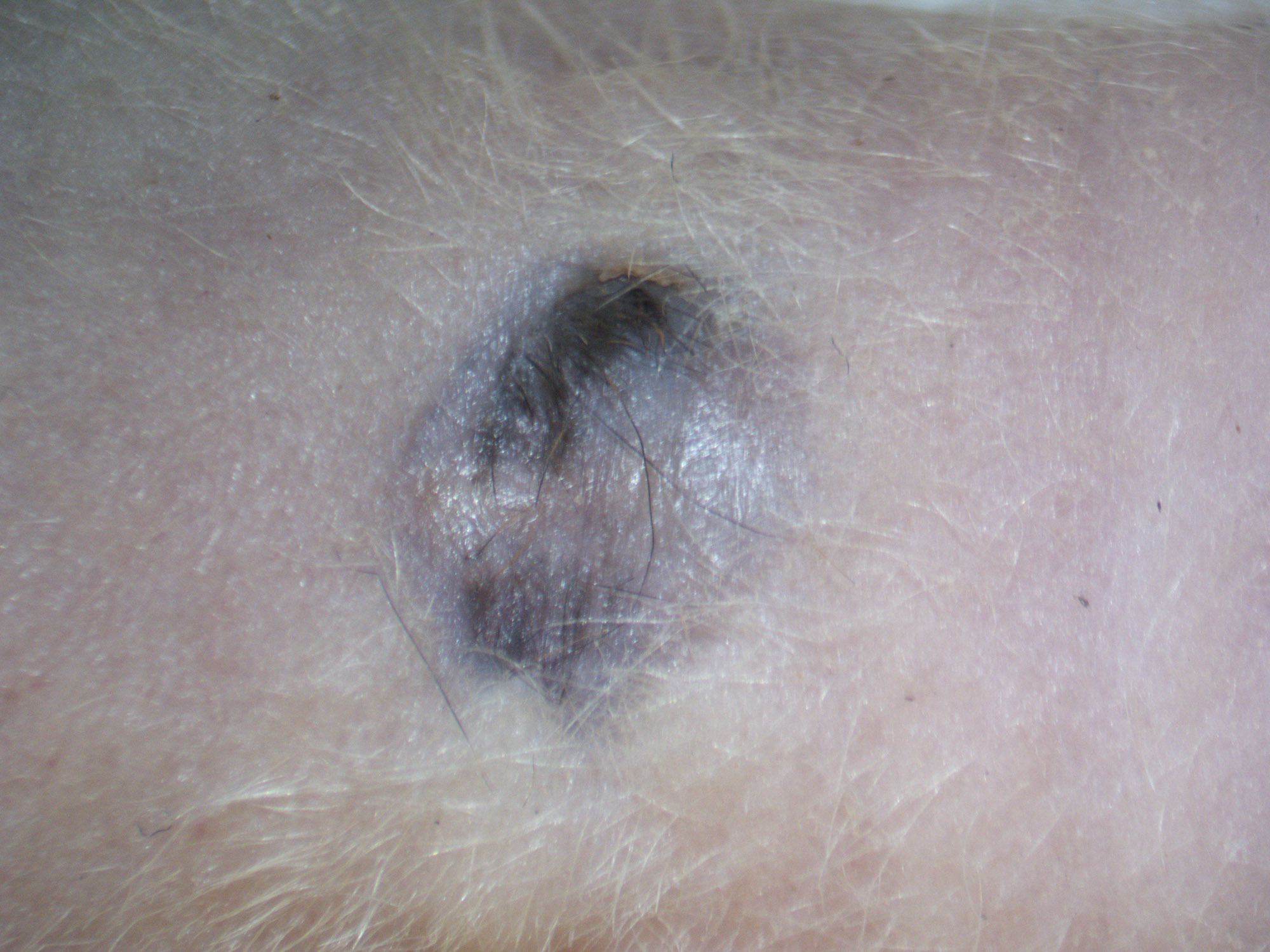

Supplement: Supplementary file 8 — Source Data for Figure 6 [file EMBJ-42-e113880-s006.zip › Fig6/6D/KO2_1.jpg]

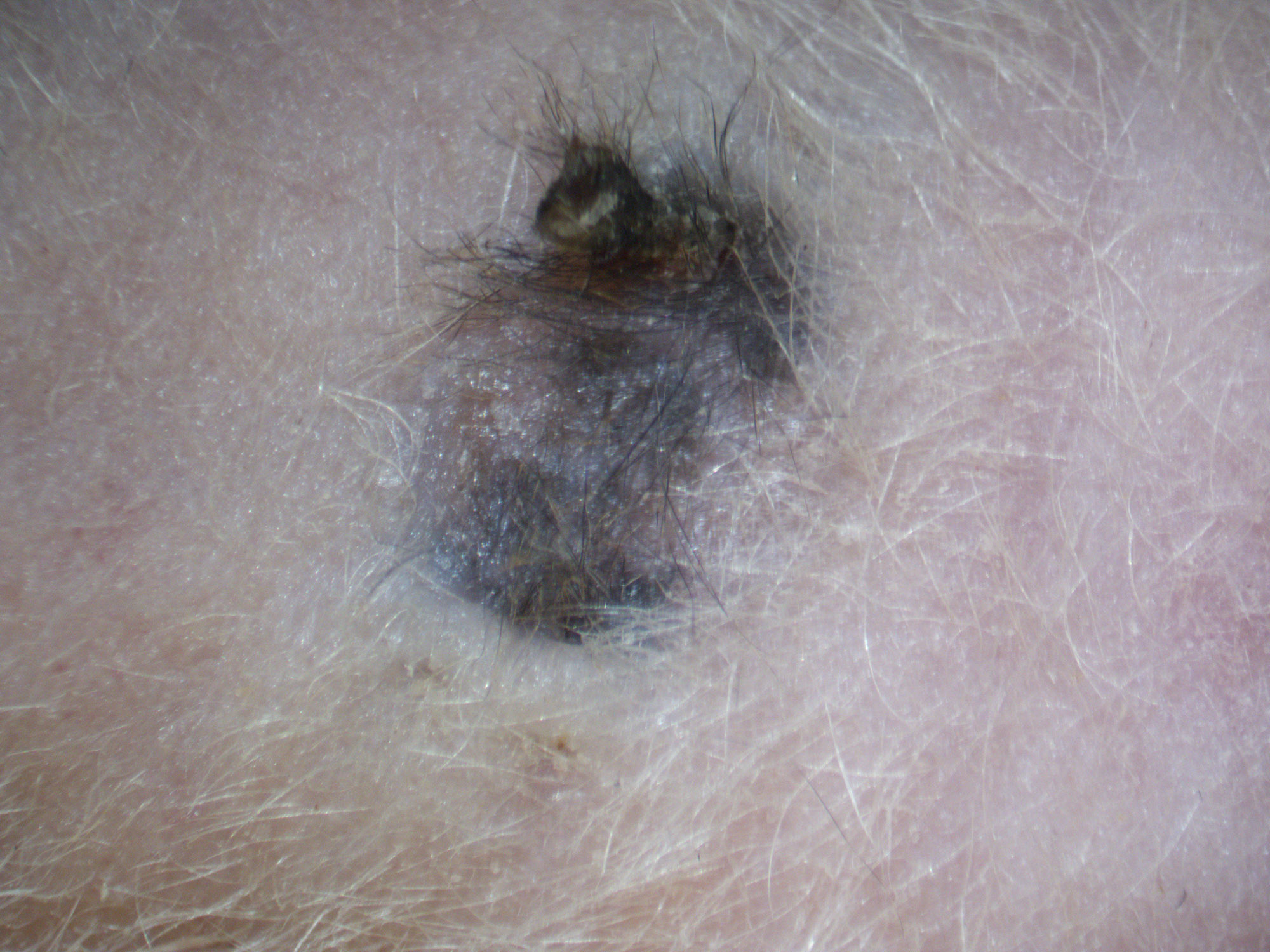

Supplement: Supplementary file 8 — Source Data for Figure 6 [file EMBJ-42-e113880-s006.zip › Fig6/6D/KO5.jpg]

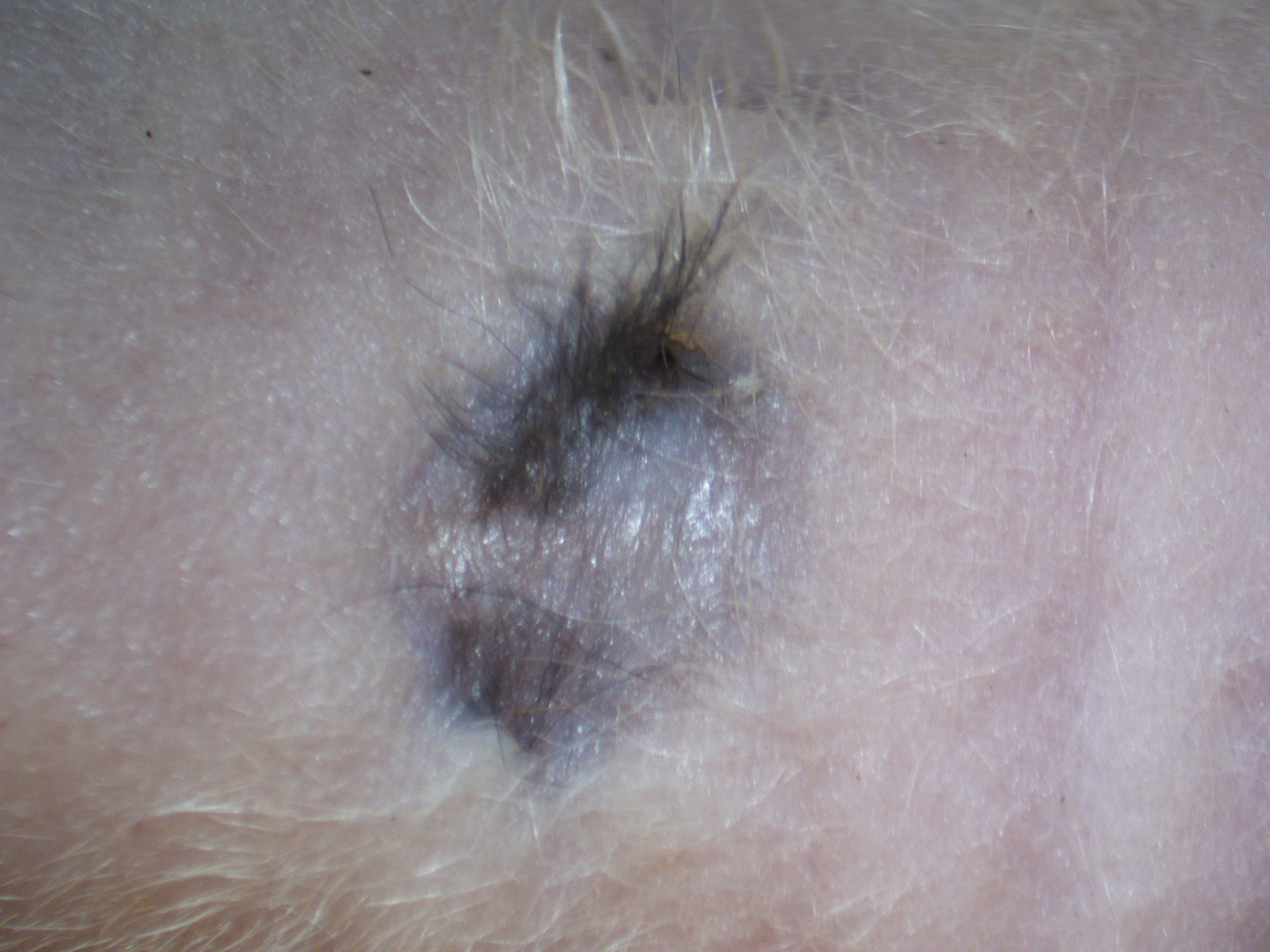

Supplement: Supplementary file 8 — Source Data for Figure 6 [file EMBJ-42-e113880-s006.zip › Fig6/6D/Ko2.jpg]

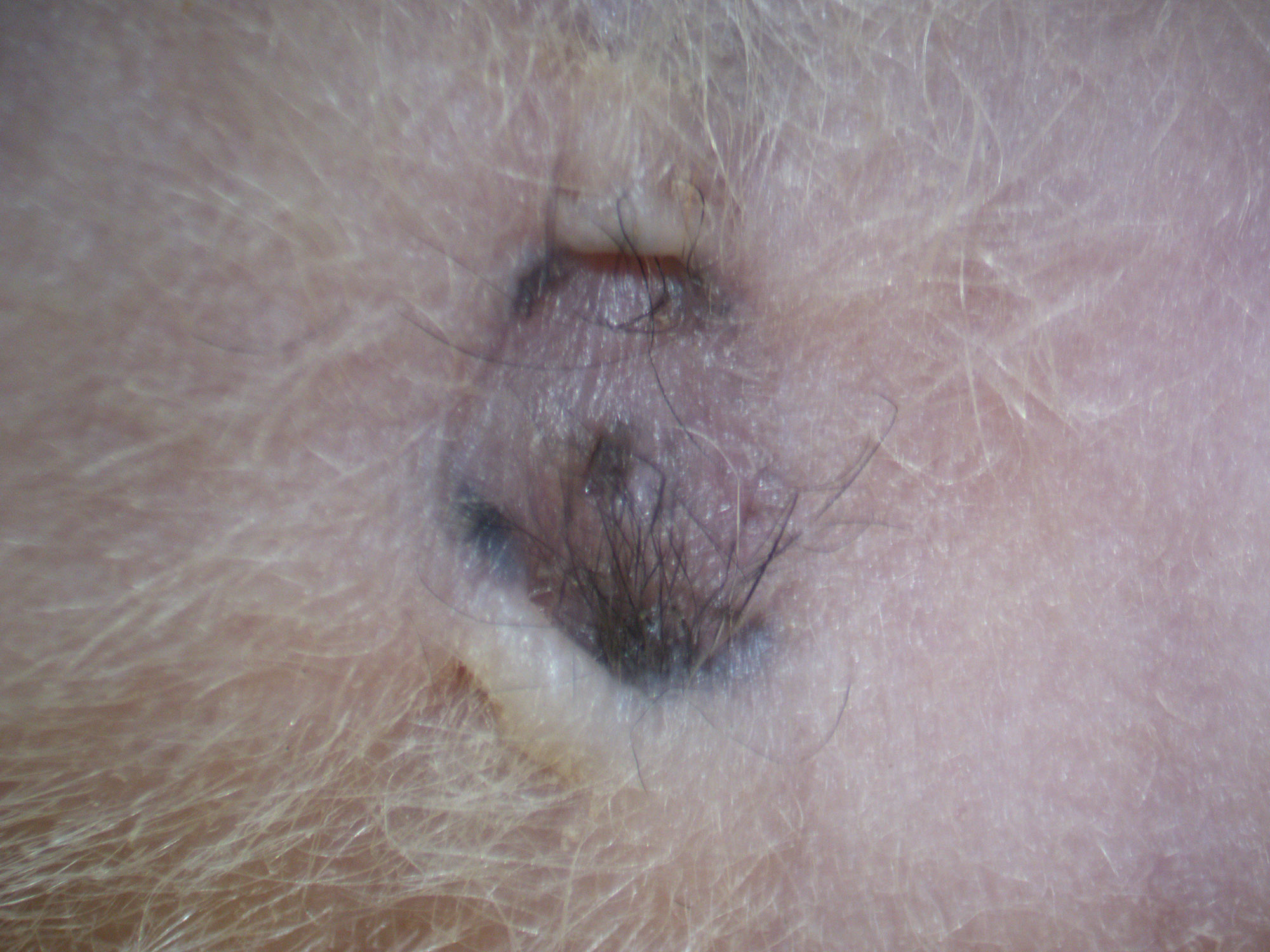

Supplement: Supplementary file 8 — Source Data for Figure 6 [file EMBJ-42-e113880-s006.zip › Fig6/6D/KO3.jpg]

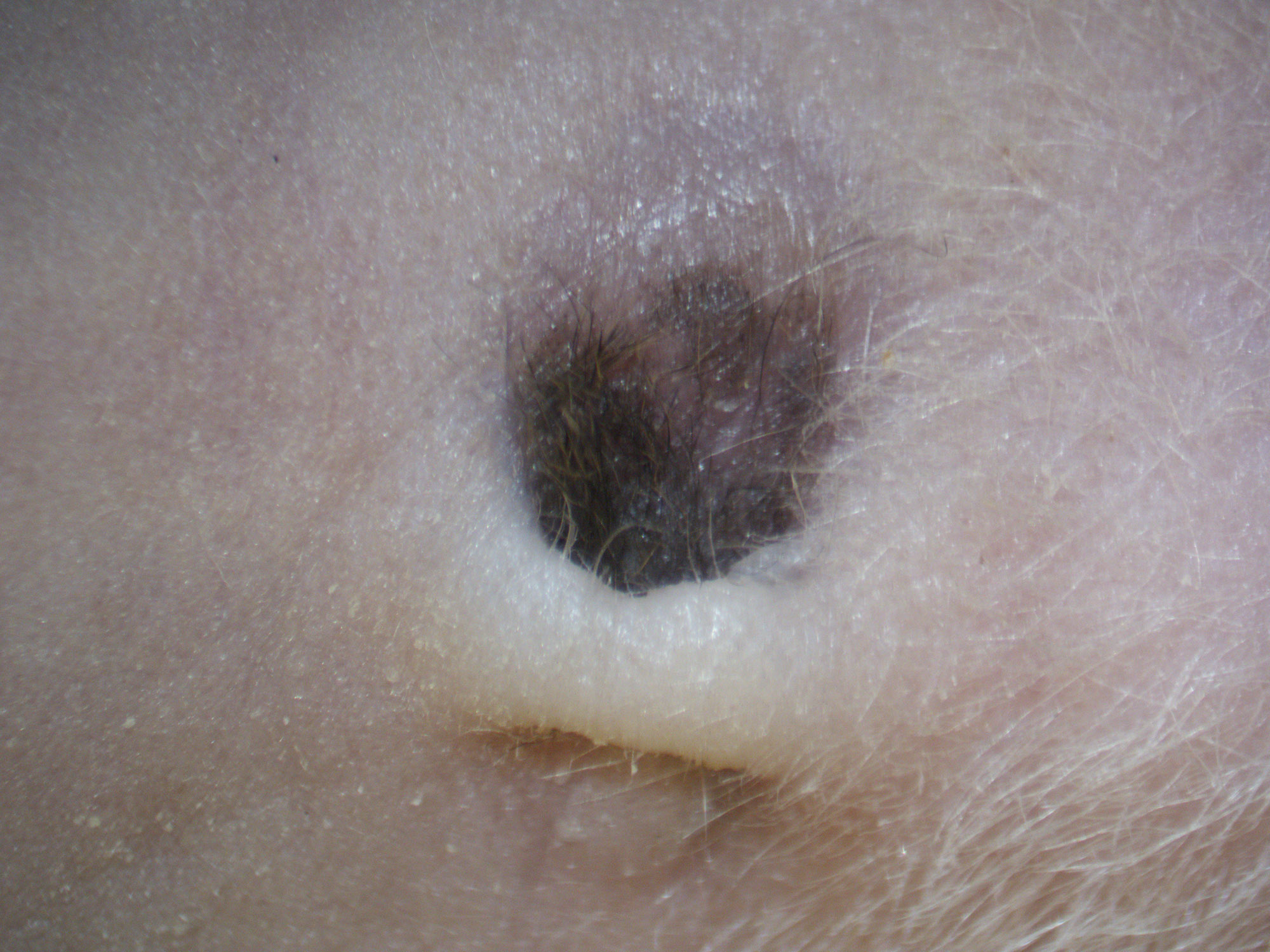

Supplement: Supplementary file 8 — Source Data for Figure 6 [file EMBJ-42-e113880-s006.zip › Fig6/6D/KO1.jpg]

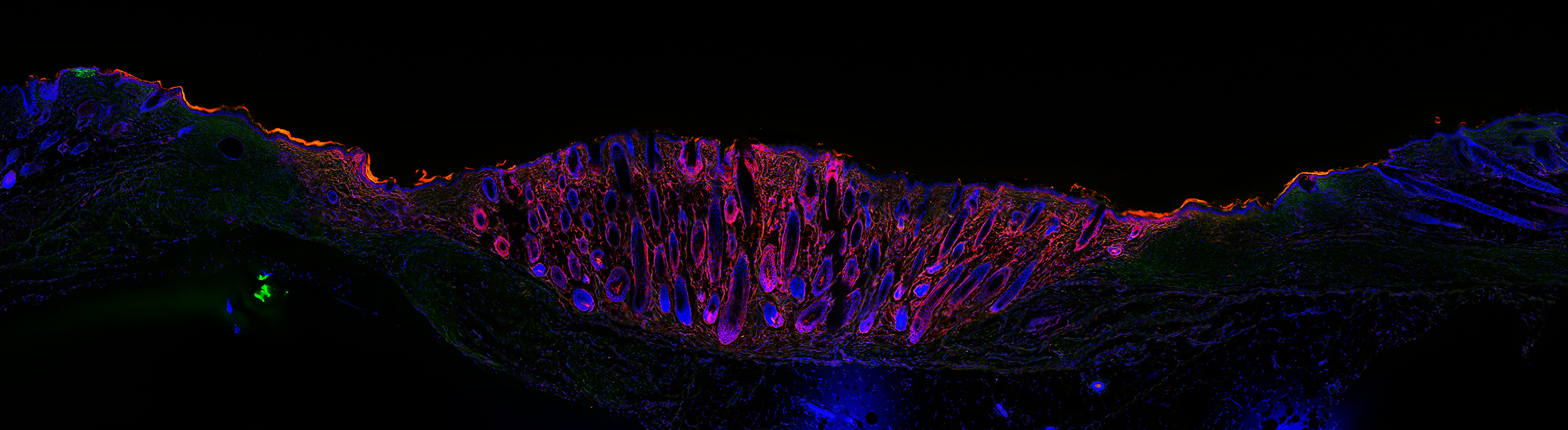

Supplement: Supplementary file 9 — Source Data for Figure 7 [file EMBJ-42-e113880-s008.zip › Fig7/7E/WT.tif]

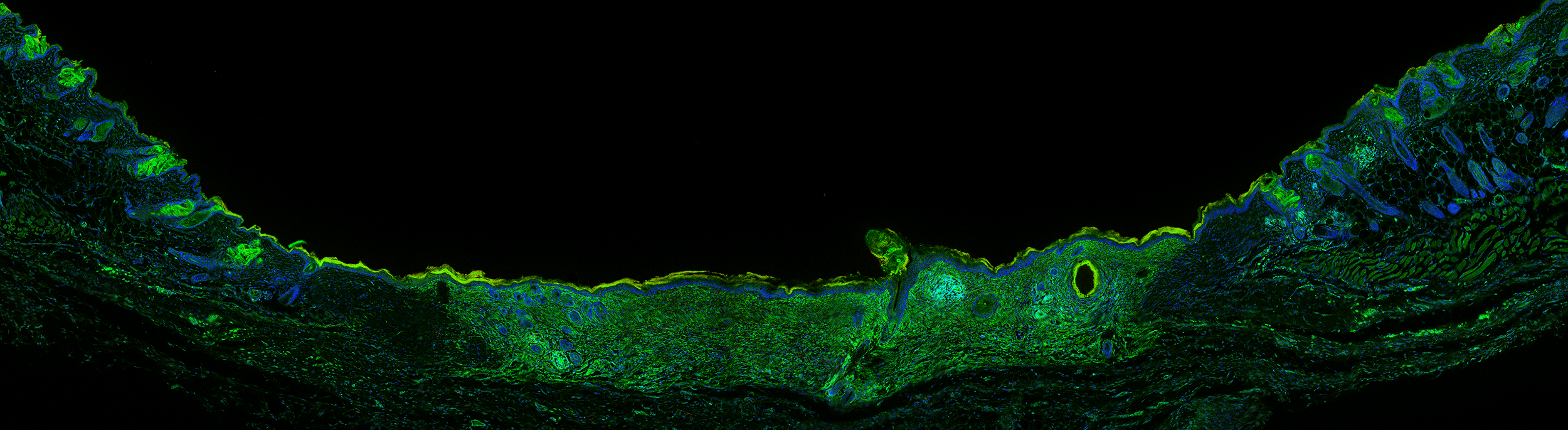

Supplement: Supplementary file 9 — Source Data for Figure 7 [file EMBJ-42-e113880-s008.zip › Fig7/7F/KO.tif]
