# Supplementary material for: Lineage commitment of dermal fibroblast progenitors is controlled by Kdm6b‐mediated chromatin demethylation
Source: EMBO J. 2023 Aug 21;42(19):e113880. doi: 10.15252/embj.2023113880 (PMC10548174; doi:10.15252/embj.2023113880)
Supplement: Supplementary file 10 [file EMBJ-42-e113880-s005.pdf]

THE EMBO JOURNAL

Lineage Commitment of Dermal Fibroblast Progenitors is Controlled by Kdm6b-mediated Chromatin Demethylation

Quan Phan, Lucia Salz, Sam Kindl, Jayden Lopez, Sean Thompson, Jasson Makkar, Iwona Driskell, and Ryan Driskell
DOI: 10.15252/embj.2023113880

Corresponding author(s): Ryan Driskell (ryan.driskell@wsu.edu)

Review Timeline:

Submission Date:	2nd Mar 23
Editorial Decision:	25th Apr 23
Revision Received:	20th Jun 23
Editorial Decision:	17th Jul 23
Revision Received:	26th Jul 23
Accepted:	1st Aug 23

Editor: Kelly Anderson

Transaction Report:

(Note: With the exception of the correction of typographical or spelling errors that could be a source of ambiguity, letters and reports are not edited. Depending on transfer agreements, referee reports obtained elsewhere may or may not be included in this compilation. Referee reports are anonymous unless the Referee chooses to sign their reports.)

Dear Dr. Driskell,

Thank you for submitting your manuscript for consideration by the EMBO Journal. It has now been seen by three referees whose comments are shown below.

Given the referees' recommendations, I would like to invite you to submit a revised version of the manuscript, addressing the comments of all three reviewers. I should add that it is EMBO Journal policy to allow only a single round of revision, and acceptance of your manuscript will therefore depend on the completeness of your responses in this revised version. I think it would be good to discuss your plan to address the referee concerns and I am available to do so by zoom or email in the coming weeks. I have attached a guide for revisions for your convenience.

When preparing your letter of response to the referees' comments, please bear in mind that this will form part of the Review Process File, and will therefore be available online to the community. For more details on our Transparent Editorial Process, please visit our website: https://www.embopress.org/page/journal/14602075/authorguide#transparentprocess

We generally allow three months as standard revision time. As a matter of policy, competing manuscripts published during this period will not negatively impact on our assessment of the conceptual advance presented by your study. However, we request that you contact the editor as soon as possible upon publication of any related work, to discuss how to proceed. Should you foresee a problem in meeting this three-month deadline, please let us know in advance and we may be able to grant an extension.

Thank you for the opportunity to consider your work for publication. I look forward to your revision.

Yours sincerely,

Kelly

Kelly M Anderson, PhD
Editor
The EMBO Journal
k.anderson@embojournal.org

Further information is available in our Guide For Authors: https://www.embopress.org/page/journal/14602075/authorguide

We realize that it is difficult to revise to a specific deadline. In the interest of protecting the conceptual advance provided by the work, we recommend a revision within 3 months (24th Jul 2023). Please discuss the revision progress ahead of this time with the editor if you require more time to complete the revisions.

Referee #1:

The study of Phan et al. is very timely and certainly of high interest to a wide audience. The authors focus on fibroblast development in the skin, from the indication of lineage commitment (transcriptional traits) to their maturation that endows them with their biological function - such as the ability to induce hair follicles. The authors first set the stage where they convincingly show that E14.5 dermal fibroblast progenitors (DFP) are not capable to support hair follicle induction in vivo (via an elegant transplantation approach), whereas E18.5 fibroblasts do. To interrogate the mechanism, they investigate the chromatin accessibility during fibroblast lineage commitment (utilizing scATAC-seq), concluding that chromatin de-repression is key for gaining a hair follicle inductive function. Diving further into the mechanism, they show that this chromatin programming of DFPs associates with the removal of repressive H3K27me3 marks, as well as that the Kdm6b/Jmjd3 (a histone demethylase) is critically important for the DFPs' differentiation.

Overall, this an exemplary study making the step-wise exploration of the timely research question 'how DFPs differentiate into distinct fibroblast lineages' accessible to a wide audience. The authors answer this question by combining a multitude of methods, such as scRNA-seq, scATAC-seq, CHIP-seq, in vivo tissue reconstitution, and targeted gene knockout models, and explain every step in an easy-to-follow way throughout the text. Moreover, their findings in skin and fibroblasts biology are novel, the study is of high quality (data and analysis), and the authors provide an intuitive open-access tool making the data available

to the research community. To further strengthen the manuscript, as the authors will see in the comments below, some parts need further attention and should be addressed.

Major comments:

(1) Overall, please carefully check all your plots and their respective legends in the figures, and the text throughout the manuscript. For example, in Fig1 D-E, the cluster numbers on the plot and in the legend don't match: dermal sheath is cluster 10 on the plot but cluster 12 in the legend. Or, for Fig 2E, the authors say in one sentence that DP cells were present in scATAC-seq data, but in the following sentence they say that DP were not detected. Do they mean fascia cells instead?

(2) Interpreting RNA-velocity results, the authors highlight fascia and pre-adipocyte differentiation as notable differentiation trajectories for lower DFPs. However, the RNA-velocity arrow streams show inverted trajectories for both of these populations. Similar inversion of differentiation trajectories in RNA-velocity analysis have been observed in cases where mRNA splicing and degradation kinetics change during developmental and differentiation processes (as discussed in Bergen et al., 2021, Barile et al., 2021). Thus, it would be important to corroborate these findings, by for example showing the changes in spliced and un-spliced genes involved in pre-adipocyte and fascia differentiation.

¬(3) In Fig 1G, the 'reticular differentiation arm' toward the pre-adipocyte lineage shows a nice gradient-pattern of gene expression changes from E14.5 - P5. However, a similarly convincing pattern is not apparent for the DP lineage (these lineage genes look like they are not expressed at E14.5) in contrast to what's stated in the text. Similarly, in Fig 2F, a continuous transition of upper DFPs to DP lineage in papillary differentiation is not obvious, which again is more convincing in the reticular differentiation arm. Please clarify this part either by down-toning/revising the text or updating the figure presentation.

(4) Fig 3I shows the expression levels of H3K27me3 modifiers in E14.5 and E18.5 timepoints. It could be more informative to have these timepoints subdivided into clusters, to see if these genes are active in specific cell types.

(5) When discussing Fig 4 latent time between WT and Kdm6b-cKO mice (Fig 4J), the authors claim that "the analysis revealed changes in the terminally differentiated cell types where Kdm6b-cKO fibroblast shifted differentiated cell types from pre-adipocytes to Replicating fibroblasts". Were these 2 latent time analyses performed independently of each other? Could it rather be that the lack of pre-adipocytes (the major endpoint in WT cells) in the Kdm6b-cKO dataset "shifts" the latent time values for the remaining cells up - ie. the replicating fibroblasts with high latent time in the KO dataset could be at the same differentiation state as their counterparts in the WT dataset, just that in the KO dataset they are relatively far along because pre-adipocytes are not present? As the WT and Kdm6b-cKO datasets are already integrated (Fig 4H), could the authors perform latent time analysis on the combined data, thus showing a more aligned representation of the differentiation status for these cells?

(6) In Fig 4J, why do the WT and Kdm6b-cKO PAGA plots look different? It's not obvious why the representation in 4J KO changes even though it does not change in L and M.

(7) In Fig. 4, the authors show that Kdm6b-KO mice develop stunted HFs with smaller DPs and describe how the chromatin in these cells is closed, inhibiting terminal differentiation. This is an interesting finding and could be further explored. For example, do these E18.5 KO DPs resemble E14.5 dermal condensate cells that have been incorporated into a DP-like structure? Is the dermal sheet development also affected in these KO mice? As the presented H&E images of the KO mice show HF-structures that look rather similar in size and shape, is it that there is only 1 wave of HF development (ie. guard hairs), or do all hair follicle types start to develop "normally" but then they stop all at the same stage? Answers to these questions can be shown and/or discussed.

(8) In association with the GO analyses (eg. Figs 3, 4, 6), please provide volcanoplots highlighting the genes on which the corresponding GO analysis was based on.

(9) Overall, how many biological replicates were used for each of the different experiments? As presented, it looks like most sequencing samples come from single replicates, though, several sampled embryonic timepoints per experimental question. Given the relatively high number of cells, were samples pooled from different mice? Please clearly state the number of biological replicates, sample pooling (if), etc. at least in the methods part. If several replicates/timepoint and experiment type are available, highlighting the independent sample contributions on the UMAP is recommended.

Minor:

- In Fig 1A, the H&E images appear quite blue, recommended to white-balance them.
- Fig 1: On several panels it's written that E18.5 timepoint was used, according to text it should be E17.5? Please update to the correct timepoint everywhere.
- Fig 4E: Panel E shows quantification of "Number of DP per HF", which should supposedly be number of DP cells per HF?
- Fig 4 H. It's very difficult to see WT cells.
- Fig 5B. Tick labels and tick marks are not aligned/off.

Referee #2:

This study by Phan et al. aims to elucidate the mechanism of chromatin organization and lineage-specification potential of dermal fibroblast progenitors (DFP). Using a HF reconstitution assay, the show that E14.5 dermal fibroblast progenitors (DFP) do not form skin with appendages (e.g. hair follicles) whereas E18.5 and P5 DFP can form appendages. To explain this phenomenon, they use H3K27-me3 ChIP-seq to show that both global chromatin profile and lineage specific genes (e.g. DP-specific, adipocyte-specific) are more enriched for H3K27-me3 and repressed at E14.5 compared to E18.5. They hypothesize that loss of H3K27-me3 over time in DFP is mediated by the demethylase, Kdm6b. Constitutive dermal Kdm6b-KO yields a reduction of DP formation (20% less CD133+ cells) and leads to less HF formation in HF reconstitution with E18.5 cells. With this data, they suggest that the de-repression of DP-lineage-specific genes by H3K27-me3 demethylase, Kdm6b/Jmjd3, explains the hair-inducing ability of DFP seen in later embryonic stage. The epigenetic assessment of DFPs across time is interesting; however, the knockout of a globally important demethylase does not yield specific biological insight into how lineage specification occurs. Additionally, throughout the manuscript, there is a lack of necessary explanations regarding both experimental and analytical methodology, which made it interpretation of figures challenging.

Major:
1. It is not surprising that if at E14.5 there are not many differentiated DC/DP cells, the DP-lineage-specific genes may be repressed and methylated. The novel point that the authors seem to make is the specific demethylase that governs the lineage specification of DFPs. However, H3k27-me3 along with the TF it governs seem ubiquitous (Fig. 5G), and broadly employed by different lineages such as DP, adipocytes etc. Therefore, the mechanistic insight into lineage specification is lacking.
2. Overall, the rationale and procedure of methods used needs to be better explained and justified. For example, in Fig 1H, it is not clear what the tdTomato is a reporter is labeling. In Fig 2F, it is not clear what the meaning of the peak analysis is and what genes they correspond to. Additionally, much of the computational analysis are not clearly well-based. For example, the legitimacy of batch-correcting and running PAGA and RNA velocity analysis on scRNA-seq data from discrete time points was not clearly justified or explained.
3. The authors conducted ChIP-seq after culturing, but used freshly isolated cells for hair follicle reconstitution assay according to the experiment schematic. It is not clear if the epigenetic profile of those cultured cells will be similar to that of the freshly isolated cells and thus it's unclear if the profiles can explain the HF reconstitution results.
4. It appears that the constitutive knockout of Kdm6b produces a global effect and probably affected many more genes beyond just lineage-specific genes. Also, could the authors do experiments such as adding demethylase drug to E14.5 cells and see if that improves HF reconstitution results?

Minor:
1. There are a couple of annotation errors throughout, e.g. E17.5 in Figure 1 and E18.5 in Figure 2, and E17.5 and E18.5 are referred to interchangeably in text.
2. In Figure 5F, the p-values for Homer motifs seem relatively large. It would be helpful if a ranking is provided.

Referee #3:

The study is interesting because it reveals some mechanisms involved in the differentiation process of dermal fibroblasts in the period between E14.5 to E18.5 using a murine model. The modification of the chromatin structure, the activity of the demethylase Kdm6b/ Jmjd3 and the evaluation of lineage-specific genes and pluripotency are shown. The research group has experience in the field as evidenced by the information contained on the website https://skinregeneration.org/. The figures are very illustrative and the results are shown clearly and forcefully.
The use of multimodal single-cell approaches, epigenetic assays, and allografting techniques is fine, but I believe that confirmation of the gene expression by real-time rt-pcr assayis necessary, as well as the solid-phase immunodetection assays to validate their results at protein level.
My specific comments focus on the following:
Abstract: I suggest that it should be written in the ordinary format where the objective is clearly mentioned, as well as the methods used (the method for tissue-specific deletion of Kdm6b/Jmjd3and the xenotransplant assay are not mentioned) and the most relevant results. At the end, include a conclusion.
A rationale about the reference of day E14.5 as a starting point for comparison with late embryonic fibroblasts could be included in the introduction section.
There are discrepancies between the information contained in the text body, figure legends and the schemes of the figures regards the days E17.5 or E18.5. Please, homogenize the correct day.
There is no figure 1J, as mentioned in the figure caption.
There are some figures that contain very small text that makes it difficult to read.I suggest increasing the size or placing figures as supplementary material.

We would like to thank the reviewers for their time in critiquing our manuscript. Overall, addressing the reviewer’s criticism the manuscript has improved in rigor and clarity.

Referee #1:

Major comments:

(1) Overall, please carefully check all your plots and their respective legends in the figures, and the text throughout the manuscript. For example, in Fig1 D-E, the cluster numbers on the plot and in the legend don't match: dermal sheath is cluster 10 on the plot but cluster 12 in the legend. Or, for Fig 2E, the authors say in one sentence that DP cells were present in scATAC-seq data, but in the following sentence they say that DP were not detected. Do they mean fascia cells instead?

-We thank the reviewer for identifying mistakes in the text. To address this, we have checked and corrected the mistakes in figures legends and text throughout the manuscript as requested.

(2) Interpreting RNA-velocity results, the authors highlight fascia and pre-adipocyte differentiation as notable differentiation trajectories for lower DFPs. However, the RNA-velocity arrow streams show inverted trajectories for both of these populations. Similar inversion of differentiation trajectories in RNA-velocity analysis have been observed in cases where mRNA splicing and degradation kinetics change during developmental and differentiation processes (as discussed in Bergen et al., 2021, Barile et al., 2021). Thus, it would be important to corroborate these findings, by for example showing the changes in spliced and un-spliced genes involved in pre-adipocyte and fascia differentiation.

This is an excellent point brought up by the reviewer. To address this concern for pre-adipocytes populations, we acknowledge that our data can only capture pre-adipocytes from E18.5 and not the mature P5 adipocytes due to the limitation of droplet-based microfluidic cell capture. This complication is most likely responsible for the inverted trajectory observed by pre-adipocytes at E18.5. We also provide additional analysis and interpretation below:

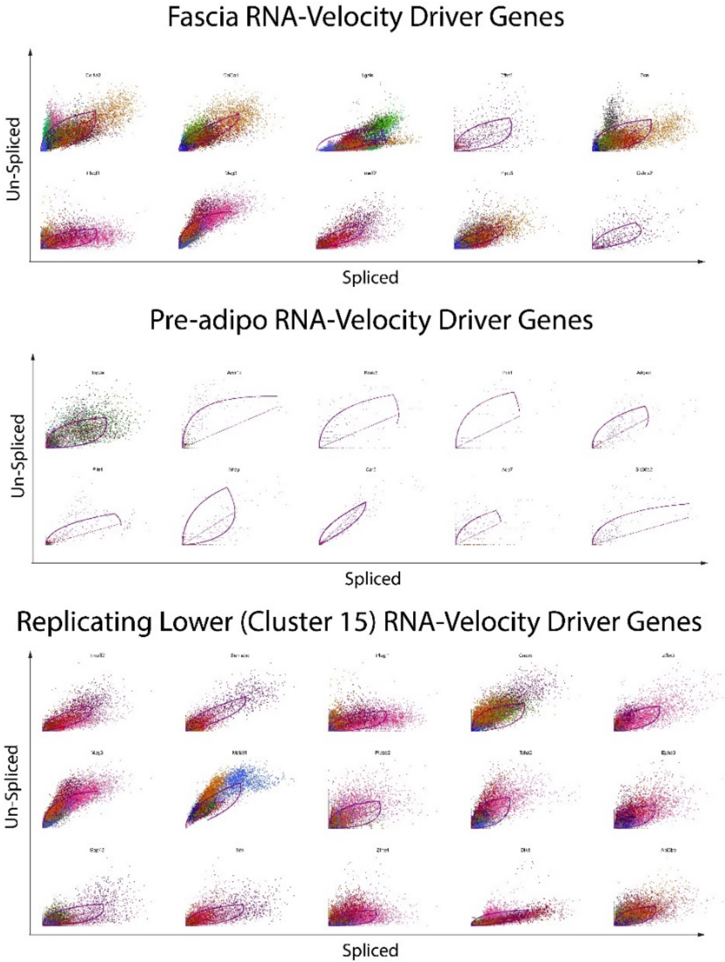

As mentioned by the reviewer, Barile et al. and Bergen et al. have also suggested that the variable kinetics rates of certain groups of genes could contribute to the velocity-inferred trajectory opposing the true differentiation

path. We have shown that the fascia RNA velocity driver genes contained a few Multiple Rate Kinetics genes (MURK) that likely turned the velocity trajectory from fascia toward replicating reticular and reticular fibroblasts.

Additionally, we have noted that a large portion of fascia is actively replicating and clustered together with replicating lower reticular (Cluster 15). This would impact the RNA velocity trajectory as well since the newly generated unspliced mRNA are much higher in this population, causing the RNA trajectory to move from fascia to replicating lower.

(3) In Fig 1G, the 'reticular differentiation arm' toward the pre-adipocyte lineage shows a nice gradient-pattern of gene expression changes from E14.5 - P5. However, a similarly convincing pattern is not apparent for the DP lineage (these lineage genes look like they are not expressed at E14.5) in contrast to what's stated in the text. Similarly, in Fig 2F, a continuous transition of upper DFPs to DP lineage in papillary differentiation is not obvious, which again is more convincing in the reticular differentiation arm. Please clarify this part either by down-toning/revising the text or updating the figure presentation.

We thank the reviewer for bringing up this concern. To address this, we have revised the heatmap in Figure 1 to reflect only the dermal condensate at E14.5 and moved it to Figure 1S. We have also revised the heatmap in Figure 2F by re-order the list of peaks to better reflect the gradient of opening chromatin regions.

(4) Fig 3I shows the expression levels of H3K27me3 modifiers in E14.5 and E18.5 timepoints. It could be more informative to have these timepoints subdivided into clusters, to see if these genes are active in specific cell types.

We thank the reviewer for this suggestion and have changed the plots to UMAPs to incorporate the different clusters, which revealed the non-specific expression levels of these genes. This is now Figure S3.

(5) When discussing Fig 4 latent time between WT and Kdm6b-cKO mice (Fig 4J), the authors claim that "the analysis revealed changes in the terminally differentiated cell types where Kdm6b-cKO fibroblast shifted differentiated cell types from pre-adipocytes to Replicating fibroblasts". Were these 2 latent time analyses performed independently of each other? Could it rather be that the lack of pre-adipocytes (the major endpoint in WT cells) in the Kdm6b-cKO dataset "shifts" the latent time values for the remaining cells up - ie. the replicating fibroblasts with high latent time in the KO dataset could be at the same differentiation state as their counterparts in the WT dataset, just that in the KO dataset they are relatively far along because pre-adipocytes are not present? As the WT and Kdm6b-cKO datasets are already integrated (Fig 4H), could the authors perform latent time analysis on the combined data, thus showing a more aligned representation of the differentiation status for these cells?

The reviewer is correct, the lack of pre-adipocytes in Kdm6b-cKO lead to the shift in latent time and predicted end points. We decided to remove the latent time analysis from the main figure but included the data here.

Combined latent time

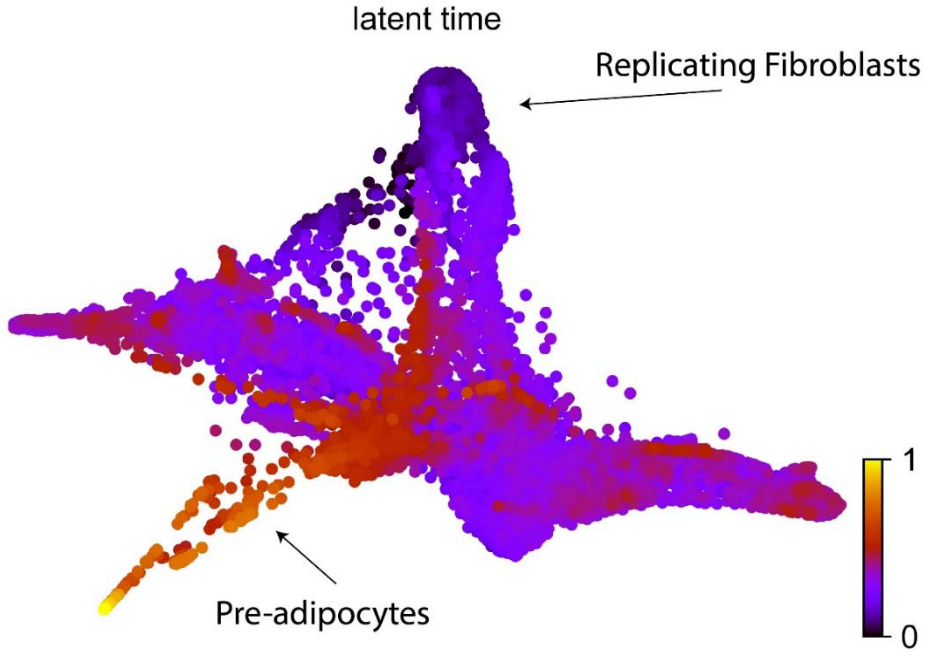

(6) In Fig 4J, why do the WT and Kdm6b-cKO PAGA plots look different? It's not obvious why the representation in 4J KO changes even though it does not change in L and M.

We thank the reviewer for bringing up this concern. 4J requires the re-analysis of subset cKO vs WT, which requires the re-cluster/calculate for latent time. 4L and M showed the same integrated analysis as 4H but presenting only cells from WT or cKO. To address this concern, we adjusted the figure to only include the integrated subset of WT and KO, instead of the individually analyzed data.

(7) In Fig. 4, the authors show that Kdm6b-KO mice develop stunted HFs with smaller DPs and describe how the chromatin in these cells is closed, inhibiting terminal differentiation. This is an interesting finding and could be further explored. For example, do these E18.5 KO DPs resemble E14.5 dermal condensate cells that have been incorporated into a DP-like structure? Is the dermal sheet development also affected in these KO mice? As the presented H&E images of the KO mice show HF-structures that look rather similar in size and shape, is it that there is only 1 wave of HF development (ie. guard hairs), or do all hair follicle types start to develop "normally" but then they stop all at the same stage? Answers to these questions can be shown and/or discussed.

The approach to study the regulation of hair follicle heterogeneity in our Kdm6b-KO mouse model is interesting but may be outside of the scope of this manuscript. However, we have addressed this comment by performing an additional chamber grafting assay and analysis to determine the types of hair follicles that could be reformed by Kdm6b-KO dermis. We used the chamber grafting assay because our Kdm6b-KO model is neonatal lethal. The results reveal that Kdm6b-KO dermal cells do not have the capacity to support zigzag hair follicle formation compared to Wild Type control dermis (graphs below). Although Kdm6b-KO grafts had hair follicles, the reformed were guard/awl/auchene hair follicles (second graph below). These new chamber grafting results suggest that hair follicle heterogeneity utilizes different epigenetic mechanisms and is potentially an exciting area of research in hair follicle biology. We found it difficult to incorporate these findings into what is already an extensive manuscript and hope that we can utilize these ideas in future studies. However, we do utilize the new data in a simplified manner for an entirely new Figure 7(A-F) and provided immunostaining results that support the

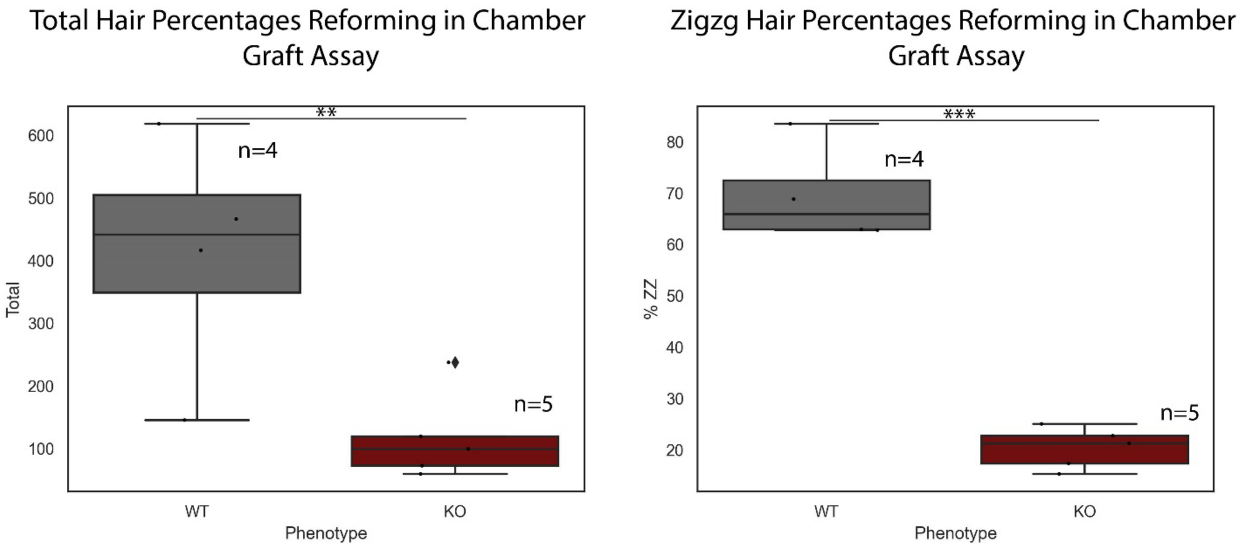

contribution of Kdm6b-KO fibroblasts to the graft.

(8) In association with the GO analyses (eg. Figs 3, 4, 6), please provide volcano plots highlighting the genes on which the corresponding GO analysis was based on.

This is an excellent suggestion by the reviewer, and we agree that genes within each GO analysis of transcriptomic data should be visualized. However, when we generated volcano plots showing all the genes within each group the plots were crowded and unreadable. Consequently, we now provide the genes associated with the GO terms in figure 3 as matrix plots in figure S4 in order to address the concern.

(9) Overall, how many biological replicates were used for each of the different experiments? As presented, it looks like most sequencing samples come from single replicates, though, several sampled embryonic timepoints per experimental question. Given the relatively high number of cells, were samples pooled from different mice? Please clearly state the number of biological replicates, sample pooling (if), etc. at least in the methods part. If several replicates/timepoint

and experiment type are available, highlighting the independent sample contributions on the UMAP is recommended.

We apologize for not stating this as clearly as possible in the original submission. We now have provided a more detailed description in the updated material and methods section with the information requested.

Minor:

- In Fig 1A, the H&E images appear quite blue, recommended to white-balance them.

We adjusted the white-balance on Figure 1A H&E images.

- Fig 1: On several panels it's written that E18.5 timepoint was used, according to text it should be E17.5? Please update to the correct timepoint everywhere.

The time point is E18.5. We have updated the timepoints to be correct.

- Fig 4E: Panel E shows quantification of "Number of DP per HF", which should supposedly be number of DP cells per HF?

We have added ‘Cells’ into the figure as requested.

- Fig 4 H. It's very difficult to see WT cells.

We have generated a new panel that separates the WT and KO umaps to make the WT cells more visible.

- Fig 5B. Tick labels and tick marks are not aligned/off.

We have aligned the numbers and tick marks.

Referee #2:

This study by Phan et al. aims to elucidate the mechanism of chromatin organization and lineage-specification potential of dermal fibroblast progenitors (DFP). Using a HF reconstitution assay, the show that E14.5 dermal fibroblast progenitors (DFP) do not form skin with appendages (e.g. hair follicles) whereas E18.5 and P5 DFP can form appendages. To explain this phenomenon, they use H3K27-me3 ChIP-seq to show that both global chromatin profile and lineage specific genes (e.g. DP-specific, adipocyte-specific) are more enriched for H3K27-me3 and repressed at E14.5 compared to E18.5. They hypothesize that loss of H3K27-me3 over time in DFP is mediated by the demethylase, Kdm6b. Constitutive dermal Kdm6b-KO yields a reduction of DP formation (20% less CD133+ cells) and leads to less HF formation in HF reconstitution with E18.5 cells. With this data, they suggest that the de-repression of DP-lineage-specific genes by H3K27-me3 demethylase, Kdm6b/Jmjd3, explains the hair-inducing ability of DFP seen in later embryonic stage. The epigenetic assessment of DFPs across time is interesting; however, the knockout of a globally important demethylase does not yield specific biological insight into how lineage specification occurs. Additionally, throughout the manuscript, there is a lack of necessary explanations regarding both experimental and analytical methodology, which made it interpretation of figures challenging.

We have now made an effort to make the figures clearer and more approachable. For example, we have moved some of the complicated concepts to supplemental figures (i.e. dual function transcription factors). We have also expanded the number of figures to simplify the interpretations of our datasets. In addition, the comments from all reviewers have helped us improve the manuscript to streamline our narrative around the novelty of our work.

Major:

1. It is not surprising that if at E14.5 there are not many differentiated DC/DP cells, the DP-lineage-specific genes may be repressed and methylated. The novel point that the authors seem to make is the specific demethylase that governs the lineage specification of DFPs. However, H3k27-me3 along with the TF it governs seem ubiquitous (Fig. 5G), and broadly employed by different lineages such as DP, adipocytes etc. Therefore, the mechanistic insight into lineage specification is lacking.

Although it might not be surprising that E14.5 dermal cells cannot differentiate into DP cells, we hope that our manuscript provides an epigenetic explanation as to why the early embryonic fibroblasts cannot differentiate into DP cells, which until now was currently unknown.

Furthermore, the large amount of genomic data and mechanistic understanding of chromatin remodeling that is in our manuscript provides foundational data that can be utilized by many laboratories that study epithelial mesenchymal interactions during embryogenesis. Consequently, we have converted the manuscript to be a resource article in EMBO.

2. Overall, the rationale and procedure of methods used needs to be better explained and justified. For example, in Fig 1H, it is not clear what the tdTomato is a reporter is labeling. In Fig 2F, it is not clear what the meaning of the peak analysis is and what genes they correspond to. Additionally, much of the computational analysis are not clearly well-based. For example, the

legitimacy of batch-correcting and running PAGA and RNA velocity analysis on scRNA-seq data from discrete time points was not clearly justified or explained.

We apologize for not being clear in explanation of our computational approaches and hope that changing the format to a Resource Article addresses this concern since detailed explanations are required in the Methods and Protocols section and Reagents and Tools table, which we now provide.

In Figure 1H the tdTomato was a reporter driven by the ROSA-CMV promoter. Consequently, tdTomato was constitutively active in the dermal cells seeded into the chamber graft. We utilized these transgenic reporter cells as a control to verify that E14.5 dermis populated the chamber graft even though hair follicles were not visible. We have adjusted the figure to address this concern.

For the time points scRNA-seq data in figure 1, we set out to investigate the differentiation trajectories of dermal progenitor fibroblasts across three different time points. Without the integration using Scanorama, the batch effect could be observed where E14.5, E18.5, and P5 fibroblasts all clustered separately. We performed Scanorama integration to establish the continuous transition states of fibroblasts during embryonic development (Hie et al., 2019)

Partition based graph abstraction provides an interpretable graph-like map based on estimating connectivity of manifold partitions, where it preserves the global topology of data and results in much higher computational efficiency of the continuous cell transitions (Wolf et al., 2019).

3. The authors conducted ChIP-seq after culturing, but used freshly isolated cells for hair follicle reconstitution assay according to the experiment schematic. It is not clear if the epigenetic profile of those cultured cells will be similar to that of the freshly isolated cells and thus it's unclear if the profiles can explain the HF reconstitution results.

We agree with the reviewer and acknowledge that the culturing of ChIP-seq will influence the epigenetic of the isolated fibroblasts. Hence, after the chamber grafting assay, we performed single-cell ATAC-seq assay testing the chromatin profiles of freshly isolated single cells, similar to the skin reconstitution assay, which supported our ChIPseq studies. We believe that it is outside the scope of our study, but with the emergence of scCUT&TAG, it is now possible to profile the epigenetic changes at the single cell level using freshly isolated cells, and we will consider this assay for our future study.

4. It appears that the constitutive knockout of Kdm6b produces a global effect and probably affected many more genes beyond just lineage-specific genes. Also, could the authors do experiments such as adding demethylase drug to E14.5 cells and see if that improves HF reconstitution results?

The reviewer is correct in that our Kdm6b-KO model there is a global effect of genes beyond the lineage-specific genes, we have addressed the upregulated genes in cKO-Kdm6b analysis, which showed an increase in nucleus-protein transport and other chromatin organization genes in Figure S4D-E.

We have also tried to perform demethylase inhibitor GSKJ4 during embryonic development. However, the results of this experimental approach are inconsistent with the predicted function of GSKJ4 where we see a significant decrease in H3K27me3 levels in GSKJ4 treated mice. Therefore, we believed that this experimental approach, even though of interest, is outside the scope of this study.

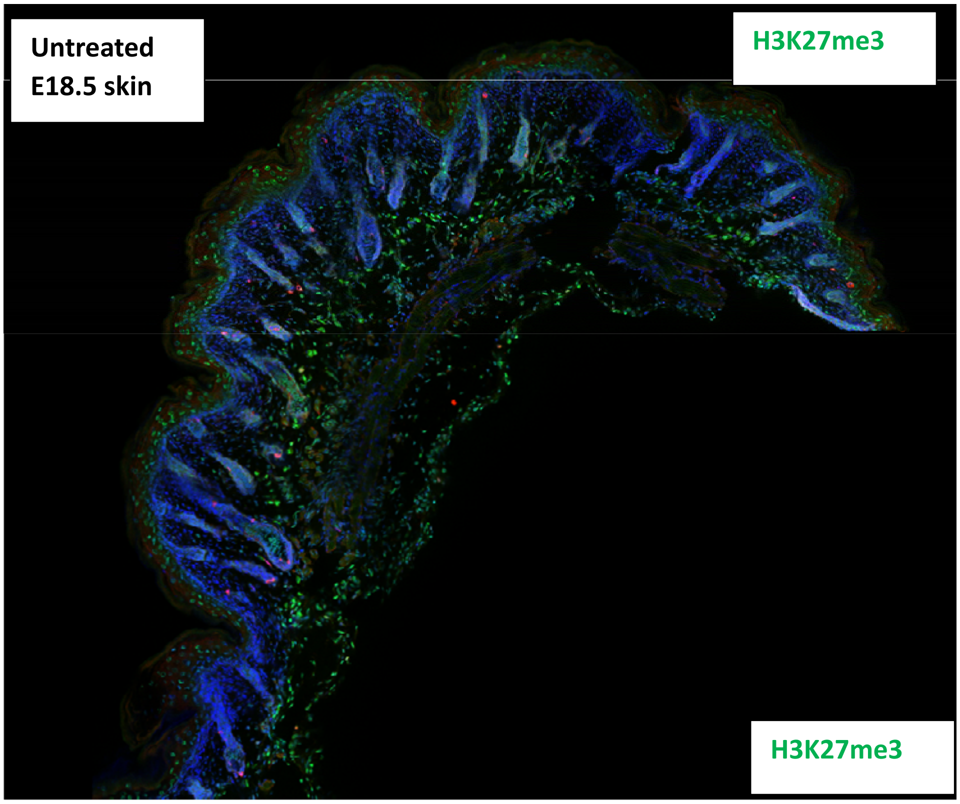

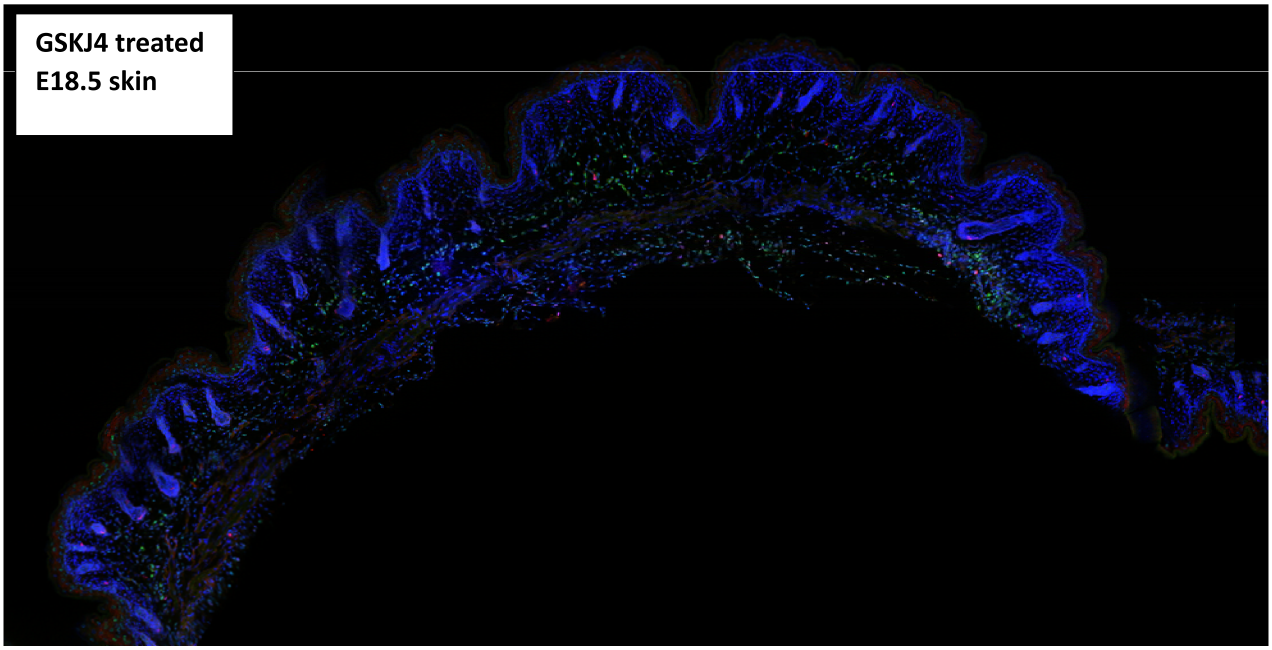

Minor:

1. There are a couple of annotation errors throughout, e.g. E17.5 in Figure 1 and E18.5 in Figure 2, and E17.5 and E18.5 are referred to interchangeably in text.

We have changed the annotation errors and all are labeled E18.5.

2. In Figure 5F, the p-values for Homer motifs seem relatively large. It would be helpful if a ranking is provided.

TCFL2 and EBF1 are ranked 9 and 10 respectively. The higher ranked motifs included Ptf1a, Ascl1, Eomes, Hoxd13 were not expressed in the scRNA-seq data at this timepoint (skinregeneration.org), and we decided to focus on biologically relevant transcription factors.

Referee #3:

The study is interesting because it reveals some mechanisms involved in the differentiation process of dermal fibroblasts in the period between E14.5 to E18.5 using a murine model. The modification of the chromatin structure, the activity of the demethylase Kdm6b/ Jmjd3 and the evaluation of lineage-specific genes and pluripotency are shown. The research group has experience in the field as evidenced by the information contained on the website https://skinregeneration.org/. The figures are very illustrative and the results are shown clearly and forcefully.

The use of multimodal single-cell approaches, epigenetic assays, and allografting techniques is fine, but I believe that confirmation of the gene expression by real-time rt-pcr assays necessary, as well as the solid-phase immunodetection assays to validate their results at protein level.

We thank the reviewer for this comment. However, our approach was to investigate chromatin dynamics during skin development, which might not always correlate with gene and protein expression in vivo. We hope that this explanation and the new chamber grafting assays focusing on functional outcomes will address this concern.

My specific comments focus on the following:

Abstract: I suggest that it should be written in the ordinary format where the objective is clearly mentioned, as well as the methods used (the method for tissue-specific deletion of Kdm6b/Jmjd3and the xenotransplant assay are not mentioned) and the most relevant results. At the end, include a conclusion.

A great suggestion by this reviewer. We have modified the abstract as suggested.

A rationale about the reference of day E14.5 as a starting point for comparison with late embryonic fibroblasts could be included in the introduction section.

We have added the reference in our introduction.

There are discrepancies between the information contained in the text body, figure legends and the schemes of the figures regards the days E17.5 or E18.5. Please, homogenize the correct day.

We are sorry for the error. We have aligned the time points throughout the text.

There is no figure 1J, as mentioned in the figure caption.

We have edited this part of the figure so that there is no error.

There are some figures that contain very small text that makes it difficult to read. I suggest increasing the size or placing figures as supplementary material.

The reviewer is correct. We have enlarged text in figures, rearranged the figures, and moved different panels to generate new figures to increase clarity of the details in the manuscript. This has resulted in having 7 figures with 6 supplemental figures.

Dear Ryan,

Congratulations on a great revision! Overall, the referees have been positive. However the referees bring up a few concerns that I ask you to address (non-experimentally) in a revised version.

When you submit your revised version, please also take care of the following editorial items and add this also to your point-by-point response:

1. Please add the following funding information to our online eJP system: National Institutes of Health SIG grant (#S10 OD026929) and Poncin Fellowship

2. Up to five keywords, which may or may not appear in the title, should be given in alphabetical order, below the abstract, each separated by a slash (/).

3. Please review our new policy on conflict of interests on the EMBO author guide website and update the title of this section to: Disclosure and competing interests statement

4. Please remove the author contributions section from the main manuscript

5. Each figure needs to be referred to in the main manuscript, and in chronological order. Please include a reference to figure 1H and figure 7C.

6. Please rename Figure S1-S7 to Figure EV1-EV7 (or EV1-EV5, and the 2 remaining figures include in the Appendix PDF with the nomenclature Appendix Figure S1-S2, with the appropriate names corrected also in the main manuscript.

7. We require the publication of source data, particularly for electrophoretic gels and blots and graphs, with the aim of making primary data more accessible and transparent to the reader. Please provide a PDF file per figure that contains the original, uncropped and unprocessed scans of all or key gels used in the figure or for graphs, an Excel spreadsheet with the original data used to generate the graphs. The PDF files should be labeled with the appropriate figure/panel number, and should have molecular weight marker; further annotation could be useful but is not essential. The PDF files will be published online with the article as supplementary "Source Data" files.

8. The synopsis image is too large, it should be 550 pixels wide by 200-400 pixels high.

Thank you for the opportunity to consider your work for publication. I look forward to your revision.

Kind regards,
Kelly

Kelly M Anderson, PhD
Editor, The EMBO Journal
k.anderson@embojournal.org

Further information is available in our Guide For Authors: https://www.embopress.org/page/journal/14602075/authorguide

----

Referee #1:

The authors have done a great job in addressing my concerns and I have no further requests. I particularly appreciated the additional experiments presented in figure 7 (WT vs Kdm6b-KO chamber grafts) which certainly answered my question if all HF types start to develop normally and are stalled in the same stage, or if the development of only certain HF types is supported. The result is highly interesting, and I do agree that detailed investigation of this differential hair-type support is out of scope for this manuscript. Overall, the study is very timely, well conducted and well written (i.e. easy to follow given the complexity of methods and analyses used).

Referee #2:

This study by Phan et al. provides a resource to study chromatin organization and lineage-specification potential of dermal fibroblast progenitors (DFP). They use H3K27-me3 ChIP-seq to show that both global chromatin profile and lineage specific genes (e.g. DP-specific, adipocyte-specific) are more enriched for H3K27-me3 and repressed at E14.5 compared to E18.5 and thus repressed for lineage-specific fates. They hypothesize that loss of H3K27-me3 over time in DFP is mediated by the demethylase, Kdm6b. Constitutive dermal Kdm6b-KO yields a reduction of DP formation (less CD133+ cells) and leads to less hair follicle (HF) formation in HF reconstitution assays with E18.5 cells. They suggest that the de-repression of DP-lineage-specific genes by H3K27-me3 demethylase, Kdm6b/Jmjd3, explains the hair-inducing ability of DFP seen in later embryonic stage. The authors reasonably addressed most of the issues brought up in the initial review. Overall, the epigenetic assessment of DFPs across time (especially at single-cell level) is interesting and would be useful to the skin field.

Comments and suggestions:
1. Overall, the figures are dense and some figure panels are complex to interpret. For example, the information covered in Figure 3C, 6D seem to be reasonably illustrated by the heatmaps in Figure 3B and 6C. Figure 3C and 6D don't necessarily show what is written in the text referring to these figures (e.g. increased H3K27me3 in E18.5 Kdm6b-cKO), so it may be a bit confusing for readers. The manuscript could be a more accessible resource article if irrelevant figures can be cut down.
2. The manuscript may benefit from including more discussion of the assumptions and caveats of data analysis to help readers interpret the data. For example, in Figure 1E, the assumptions and caveats underlying the RNA velocity analysis of batch-corrected time-series scRNA-seq data are not discussed, given that evidence to use available methods to properly remove batch effects for RNA velocity of time-series data is currently rather limited.
3. The authors should make sure the annotations on figures are free of errors. For example, coverage plots in Figure 3H can be rescaled to show full range of signals and the gene names shouldn't be duplicated. In Figure 6A, the sorted WT cells should be tdTomato+ instead of GFP+ etc.

When you submit your revised version, please also take care of the following editorial items and add this also to your point-by-point response:

1. Please add the following funding information to our online eJP system: National Institutes of Health SIG grant (#S10 OD026929) and Poncin Fellowship.

This has been done.

2. Up to five keywords, which may or may not appear in the title, should be given in alphabetical order, below the abstract, each separated by a slash (/).

We have added the following key words :

dermal papilla / epigenetics / fibroblast progenitor / Kdm6b / multiomics

3. Please review our new policy on conflict of interests on the EMBO author guide website and update the title of this section to: Disclosure and competing interests statement.

This has been done. We have added the following to the Disclosures and competing interests statement:

The authors have no disclosures or competing interests to declare.

4. Please remove the author contributions section from the main manuscript.

We have removed the author contributions section from the main manuscript.

5. Each figure needs to be referred to in the main manuscript, and in chronological order. Please include a reference to figure 1H and figure 7C.

Figure 1H and Figure7C are now referenced.

6. Please rename Figure S1-S7 to Figure EV1-EV7 (or EV1-EV5, and the 2 remaining figures include in the Appendix PDF with the nomenclature Appendix Figure S1-S2, with the appropriate names corrected also in the main manuscript.

Figure S1-S7 have been changed to EV1-EV7. We appreciate the 2 additional EV figures.

7. We require the publication of source data, particularly for electrophoretic gels and blots and graphs, with the aim of making primary data more accessible and transparent to the reader. Please provide a PDF file per figure that contains the original, uncropped and unprocessed scans of all or key gels used in the figure or for graphs, an Excel spreadsheet

with the original data used to generate the graphs. The PDF files should be labeled with the appropriate figure/panel number, and should have molecular weight marker; further annotation could be useful but is not essential. The PDF files will be published online with the article as supplementary "Source Data" files.

The requested “Source Data” has been uploaded.

8. The synopsis image is too large, it should be 550 pixels wide by 200-400 pixels high.

The synopsis image has been resized.

Referee #1:

The authors have done a great job in addressing my concerns and I have no further requests. I particularly appreciated the additional experiments presented in figure 7 (WT vs Kdm6b-KO chamber grafts) which certainly answered my question if all HF types start to develop normally and are stalled in the same stage, or if the development of only certain HF types is supported. The result is highly interesting, and I do agree that detailed investigation of this differential hair-type support is out of scope for this manuscript. Overall, the study is very timely, well conducted and well written (i.e. easy to follow given the complexity of methods and analyses used).

Thank you. We hope that the manuscript is understood and appreciated by the scientific community.

Referee #2:

This study by Phan et al. provides a resource to study chromatin organization and lineage-specification potential of dermal fibroblast progenitors (DFP). They use H3K27-me3 ChIP-seq to show that both global chromatin profile and lineage specific genes (e.g. DP-specific, adipocyte-specific) are more enriched for H3K27-me3 and repressed at E14.5 compared to E18.5 and thus repressed for lineage-specific fates. They hypothesize that loss of H3K27-me3 over time in DFP is mediated by the demethylase, Kdm6b. Constitutive dermal Kdm6b-KO yields a reduction of DP formation (less CD133+ cells) and leads to less hair follicle (HF) formation in HF reconstitution assays with E18.5 cells. They suggest that the de-repression of DP-lineage-specific genes by H3K27-me3 demethylase, Kdm6b/Jmjd3, explains the hair-inducing ability of DFP seen in later embryonic stage. The authors reasonably addressed most of the issues brought up in the initial review. Overall, the epigenetic assessment of DFPs across time (especially at single-cell level) is interesting and would be useful to the

skin field.

Thank you for the very nice summary of the manuscript.

Comments and suggestions:

1. Overall, the figures are dense and some figure panels are complex to interpret. For example, the information covered in Figure 3C, 6D seem to be reasonably illustrated by the heatmaps in Figure 3B and 6C. Figure 3C and 6D don't necessarily show what is written in the text referring to these figures (e.g. increased H3K27me3 in E18.5 Kdm6b-cKO), so it may be a bit confusing for readers. The manuscript could be a more accessible resource article if irrelevant figures can be cut down.

We appreciate the reviewers' suggestions. Figure 3C and 6D have now been moved to Extended View Figure 3 and 6.

2. The manuscript may benefit from including more discussion of the assumptions and caveats of data analysis to help readers interpret the data. For example, in Figure 1E, the assumptions and caveats underlying the RNA velocity analysis of batch-corrected time-series scRNA-seq data are not discussed, given that evidence to use available methods to properly remove batch effects for RNA velocity of time-series data is currently rather limited.

We agree that more discussion of the limitations of transcriptomic analysis with velocity would benefit the manuscript. We have added the following discussion points in the results section discussing Figure 1E of the manuscript:

Unexpectedly, fascia and pre-adipocytes showed inverted RNA velocity trajectories toward replicating reticular fibroblasts. This observation could be due to the multiple rate kinetics genes (MURK) (Barile et al. 2021), which are associated with assumptions and caveats for Velocity analysis in scRNA-seq. For example, dynamical MURK genes can violate the assumptions that Velocity analysis is based on (Barile et al. 2021). In addition, it has been proposed that current batch correction implementations may not be accurate (Bergen et al. 2021). Consequently, it is important to test in-silico transcriptome-based results through robust independent systems.

3. The authors should make sure the annotations on figures are free of errors. For example, coverage plots in Figure 3H can be rescaled to show full range of signals and the gene names shouldn't be duplicated. In Figure 6A, the sorted WT cells should be tdTomato+ instead of GFP+ etc.

Figure 3H is now rescaled and the duplication of gene names in the coverage plots have been removed.

In Figure 6A, specifically, we were able to utilize Dermo1Cre/mtmg mice. Consequently, the sorted cells are GFP+ because they have been recombined with Cre.

Dear Ryan,

Congratulations on an excellent manuscript, I am pleased to inform you that your manuscript has been accepted for publication in the EMBO Journal. Thank you for your comprehensive response to the referee concerns and for providing detailed source data. It has been a pleasure to work with you to get this to the acceptance stage.

I will begin the final checks on your manuscript before submitting to the publisher next week. Once at the publisher, it will take about 3 weeks for your manuscript to be published online. As a reminder, the entire review process, including referee concerns and your point-by-point response, will be available to readers.

I will be in touch throughout the final editorial process until publication. In the meantime, I hope you find time to celebrate!

Kind regards,
Kelly

Kelly M Anderson, PhD
Editor, The EMBO Journal
k.anderson@embojournal.org

Please note that it is EMBO Journal policy for the transcript of the editorial process (containing referee reports and your response letter) to be published as an online supplement to each paper. If you do NOT want this, you will need to inform the Editorial Office via email immediately. More information is available here:
https://www.embopress.org/page/journal/14602075/authorguide#transparentprocess

Your manuscript will be processed for publication in the journal by EMBO Press. Manuscripts in the PDF and electronic editions of The EMBO Journal will be copy edited, and you will be provided with page proofs prior to publication. Please note that supplementary information is not included in the proofs.

You will be contacted by Wiley Author Services to complete licensing and payment information. The required 'Page Charges Authorization Form' is available here: https://www.embopress.org/pb-assets/embo-site/tej_apc.pdf - please download and complete the form and return to embopressproduction@wiley.com

EMBO Press participates in many Publish and Read agreements that allow authors to publish Open Access with reduced/no publication charges. Check your eligibility: https://authorservices.wiley.com/author-resources/Journal-Authors/open-access/affiliation-policies-payments/index.html

Should you be planning a Press Release on your article, please get in contact with embojournal@wiley.com as early as possible, in order to coordinate publication and release dates.

If you have any questions, please do not hesitate to call or email the Editorial Office. Thank you for your contribution to The EMBO Journal.

EMBO Press Author Checklist

Corresponding Author Name: Ryan R. Driskell
Journal Submitted to: The EMBO Journal
Manuscript Number: EMBOJ-2023-113880

USEFUL LINKS FOR COMPLETING THIS FORM
The EMBO Journal - Author Guidelines
EMBO Reports - Author Guidelines
Molecular Systems Biology - Author Guidelines
EMBO Molecular Medicine - Author Guidelines

Reporting Checklist for Life Science Articles (updated January
This checklist is adapted from Materials Design Analysis Reporting (MDAR) Checklist for Authors. MDAR establishes a minimum set of requirements in transparent reporting in the life sciences (see Statement of Task: 10.31222/osf.io/9sm4x). Please follow the journal's guidelines in preparing your manuscript.
Please note that a copy of this checklist will be published alongside your article.

Abridged guidelines for figures
1. Data
The data shown in figures should satisfy the following conditions:
- the data were obtained and processed according to the field's best practice and are presented to reflect the results of the experiments in an accurate and unbiased manner.
- ideally, figure panels should include only measurements that are directly comparable to each other and obtained with the same assay.
- plots include clearly labeled error bars for independent experiments and sample sizes. Unless justified, error bars should not be shown for technical
- if n<5, the individual data points from each experiment should be plotted. Any statistical test employed should be justified.
- Source Data should be included to report the data underlying figures according to the guidelines set out in the authorship guidelines on Data

2. Captions
Each figure caption should contain the following information, for each panel where they are relevant:
- a specification of the experimental system investigated (eg cell line, species name).
- the assay(s) and method(s) used to carry out the reported observations and measurements.
- an explicit mention of the biological and chemical entity(ies) that are being measured.
- an explicit mention of the biological and chemical entity(ies) that are altered/varied/perturbed in a controlled manner.
- the exact sample size (n) for each experimental group/condition, given as a number, not a range;
- a description of the sample collection allowing the reader to understand whether the samples represent technical or biological replicates (including how many animals, litters, cultures, etc.).
- a statement of how many times the experiment shown was independently replicated in the laboratory.
- definitions of statistical methods and measures:
  - common tests, such as t-test (please specify whether paired vs. unpaired), simple χ2 tests, Wilcoxon and Mann-Whitney tests, can be unambiguously identified by name only, but more complex techniques should be described in the methods section;
  - are tests one-sided or two-sided?
  - are there adjustments for multiple comparisons?
  - exact statistical test results, e.g., P values = x but not P values < x;
  - definition of 'center values' as median or average;
  - definition of error bars as s.d. or s.e.m.

Please complete ALL of the questions below.
Select "Not Applicable" only when the requested information is not relevant for your study.

Materials

Newly Created Materials	Information included in the manuscript?	In which section is the information available? (Reagents and Tools Table, Materials and Methods, Figures, Data Availability Section)
New materials and reagents need to be available; do any restrictions apply?	Yes	Material and Methods

Antibodies	Information included in the manuscript?	In which section is the information available? (Reagents and Tools Table, Materials and Methods, Figures, Data Availability Section)
For **antibodies** provide the following information:<br>- Commercial antibodies: RRID (if possible) or supplier name, catalogue number and or/clone number<br>- Non-commercial: RRID or citation	Yes	Material and Methods

DNA and RNA sequences	Information included in the manuscript?	In which section is the information available? (Reagents and Tools Table, Materials and Methods, Figures, Data Availability Section)
**Short novel DNA or RNA including primers, probes**: provide the sequences.	Not Applicable	

Cell materials	Information included in the manuscript?	In which section is the information available? (Reagents and Tools Table, Materials and Methods, Figures, Data Availability Section)
**Cell lines:** Provide species information, strain. Provide accession number in repository **OR** supplier name, catalog number, clone number, and/**OR** RRID.	Not Applicable	
**Primary cultures:** Provide species, strain, sex of origin, genetic modification status.	Yes	Material and Methods and Results
Report if the cell lines were recently **authenticated** (e.g., by STR profiling) and tested for mycoplasma contamination.	Not Applicable	

Experimental animals	Information included in the manuscript?	In which section is the information available? (Reagents and Tools Table, Materials and Methods, Figures, Data Availability Section)
**Laboratory animals or Model organisms:** Provide species, strain, sex, age, genetic modification status. Provide accession number in repository **OR** supplier name, catalog number, clone number, **OR** RRID.	Yes	Material and Methods and Results
**Animal observed in or captured from the field:** Provide species, sex, and age where possible.	Not Applicable	
Please detail **housing and husbandry conditions**.	Yes	Material and Methods

Plants and microbes	Information included in the manuscript?	In which section is the information available? (Reagents and Tools Table, Materials and Methods, Figures, Data Availability Section)
**Plants:** provide species and strain, ecotype and cultivar where relevant, unique accession number if available, and source (including location for collected wild specimens).	Not Applicable	
**Microbes:** provide species and strain, unique accession number if available, and source.	Not Applicable	

Human research participants	Information included in the manuscript?	In which section is the information available? (Reagents and Tools Table, Materials and Methods, Figures, Data Availability Section)
If collected and within the bounds of privacy constraints report on age, sex and gender or ethnicity for all study participants.	Not Applicable	

Core facilities	Information included in the manuscript?	In which section is the information available? (Reagents and Tools Table, Materials and Methods, Figures, Data Availability Section)
If your work benefited from core facilities, was their service mentioned in the acknowledgments section?	Yes	Material and Methods and Acknowledgements

Design

Study protocol	Information included in the manuscript?	In which section is the information available? (Reagents and Tools Table, Materials and Methods, Figures, Data Availability Section)
If study protocol has been **pre-registered, provide DOI in the manuscript**. For clinical trials, provide the trial registration number **OR** cite DOI.	Not Applicable	
Report the **clinical trial registration number** (at ClinicalTrials.gov or equivalent), where applicable.	Not Applicable	

Laboratory protocol	Information included in the manuscript?	In which section is the information available? (Reagents and Tools Table, Materials and Methods, Figures, Data Availability Section)
Provide DOI OR other citation details if **external detailed step-by-step protocols** are available.	Yes	References

Experimental study design and statistics	Information included in the manuscript?	In which section is the information available? (Reagents and Tools Table, Materials and Methods, Figures, Data Availability Section)
Include a statement about **sample size** estimate even if no statistical methods were used.	Yes	Results and Materials and Methods
Were any steps taken to minimize the effects of subjective bias when allocating animals/samples to treatment (e.g. **randomization procedure**)? If yes, have they been described?	Yes	Materials and Methods
Include a statement about **blinding** even if no blinding was done.	Yes	Materials and Methods
Describe **inclusion/exclusion criteria** if samples or animals were excluded from the analysis. Were the criteria pre-established?<br><br>If sample or data points were omitted from analysis, report if this was due to attrition or intentional exclusion and provide justification.	Not Applicable	
For every figure, are **statistical tests** justified as appropriate? Do the data meet the assumptions of the tests (e.g., normal distribution)? Describe any methods used to assess it. Is there an estimate of variation within each group of data? Is the variance similar between the groups that are being statistically compared?	Yes	Materials and Methods

Sample definition and in-laboratory replication	Information included in the manuscript?	In which section is the information available? (Reagents and Tools Table, Materials and Methods, Figures, Data Availability Section)
In the figure legends: state number of times the experiment was **replicated** in laboratory.	Yes	Figure Legends and Text
In the figure legends: define whether data describe **technical or biological replicates**.	Yes	Figure Legends and Text

Ethics

Ethics	Information included in the manuscript?	In which section is the information available? (Reagents and Tools Table, Materials and Methods, Figures, Data Availability Section)
Studies involving **human participants**: State details of **authority granting ethics approval** (IRB or equivalent committee(s), provide reference number for approval.	Not Applicable	
Studies involving **human participants**: Include a statement confirming that **informed consent** was obtained from all subjects and that the experiments conformed to the principles set out in the WMA Declaration of Helsinki and the Department of Health and Human Services Belmont Report.	Not Applicable	
Studies involving **human participants:** For publication of **patient photos**, include a statement confirming that consent to publish was obtained.	Not Applicable	
Studies involving experimental **animals**: State details of **authority granting ethics approval** (IRB or equivalent committee(s), provide reference number for approval. Include a statement of compliance with ethical regulations.	Yes	Materials and Methods
Studies involving **specimen and field samples:** State if relevant **permits** obtained, provide details of authority approving study; if none were required, explain why.	Not Applicable	

Dual Use Research of Concern (DURC)	Information included in the manuscript?	In which section is the information available? (Reagents and Tools Table, Materials and Methods, Figures, Data Availability Section)
Could your study fall under dual use research restrictions? Please check biosecurity documents and list of **select agents and toxins** (CDC): https://www.selectagents.gov/sat/list.htm	Not Applicable	
If you used a select agent, is the security level of the lab appropriate and reported in the manuscript?	Not Applicable	
If a study is subject to dual use research of concern regulations, is the name of the **authority granting approval and reference number** for the regulatory approval provided in the manuscript?	Not Applicable	

Reporting

The MDAR framework recommends adoption of discipline-specific guidelines, established and endorsed through community initiatives. Journals have their own policy about requiring specific guidelines and recommendations to complement MDAR.

Adherence to community standards	Information included in the manuscript?	In which section is the information available? (Reagents and Tools Table, Materials and Methods, Figures, Data Availability Section)
State if relevant guidelines or checklists (e.g., **ICMJE, MIBBI, ARRIVE, PRISMA**) have been followed or provided.	Not Applicable	
For **tumor marker prognostic studies,** we recommend that you follow the **REMARK** reporting guidelines (see link list at top right). See author guidelines, under 'Reporting Guidelines'. Please confirm you have followed these guidelines.	Not Applicable	
For **phase II and III randomized controlled trials**, please refer to the **CONSORT** flow diagram (see link list at top right) and submit the CONSORT checklist (see link list at top right) with your submission. See author guidelines, under 'Reporting Guidelines'. Please confirm you have submitted this list.	Not Applicable	

Data Availability

Data availability	Information included in the manuscript?	In which section is the information available? (Reagents and Tools Table, Materials and Methods, Figures, Data Availability Section)
Have **primary datasets** been deposited according to the journal's guidelines (see 'Data Deposition' section) and the respective accession numbers provided in the Data Availability Section?	Yes	Temporary Reference Numbers are in Materials and Methods
Were **human clinical and genomic datasets** deposited in a public access-controlled repository in accordance to ethical obligations to the patients and to the applicable consent agreement?	Not Applicable	
Are **computational models** that are central and integral to a study available without restrictions in a machine-readable form? Were the relevant accession numbers or links provided?	Yes	Materials and Methods link to Driskell Lab Github Page
If publicly available data were reused, provide the respective **data citations in the reference list**.	Not Applicable	
